# Hypervalent Iodine (III) Catalyzed Regio- and Diastereoselective Aminochlorination of Tailored Electron Deficient Olefins via GAP Chemistry

**DOI:** 10.3389/fchem.2020.00523

**Published:** 2020-07-07

**Authors:** Anis Ur Rahman, Nighat Zarshad, Peng Zhou, Weitao Yang, Guigen Li, Asad Ali

**Affiliations:** ^1^School of Chemistry and Chemical Engineering, Institute of Chemistry and BioMedical Sciences, Nanjing University, Nanjing, China; ^2^School of Chemistry and Chemical Engineering, Southeast University, Nanjing, China; ^3^Department of Chemistry and Biochemistry, Texas Tech University, Lubbock, TX, United States; ^4^Department of Chemistry, Faculty of Chemical and Life Sciences, Abdul Wali Khan University, Mardan, Pakistan

**Keywords:** aziridinium, diastereoselectivity, iodobenzene diacetate, nitrogen/halogen source, protecting groups

## Abstract

Herein, we report a protocol for highly efficient hypervalent iodine (III) mediated, group-assisted purification (GAP) method for the regioselectivities and stereoselective aminochlorination of electron-deficient olefins. A series of vicinal chloramines with multifunctionalities were acquired in moderate to excellent yields (45–94%), by merely mixing the GAP auxiliary-anchored substrates with dichloramine T and tosylamide as chlorine/nitrogen sources and iodobenzene diacetate as a catalyst. The vicinal chloramines were obtained without any column chromatographic purification and recrystallization simply by washing the reaction mixture with a minimum amount of common inexpensive solvents and thus avoiding wastage of silica, solvents, time, and labor. The GAP auxiliary is recyclable and reusable. This strategy is easy to handle, cost-effective, greener, sustainable, environmentally benign, and mostly suitable for the syntheses of vicinal haloamines from various electron-deficient alkenes.

## Introduction

Aminohalogenation of olefins is one of the most effective approaches to produce functional α,β-haloamines. These vicinal haloamines are versatile components in organic synthesis as their halogen moieties are labile to cross-coupling and substitution reactions. For example, haloamines are direct precursors to vicinal diamines (Ghorai et al., 2011; Xiong et al., 2014), α,β-dehydroamino acids (Chen et al., 2005), and aziridines (Van and De Kimpe, 2000; Schröder et al., 2017; Thakur et al., 2017). While the aminohalogenation is established for almost five decades now, it still faces shortcomings and limitations related to controlling regioselectivities and stereoselectivities, forming side products, which sometimes makes purification more difficult (Thakur et al., 2003; Bovino and Chemler, 2012).

Several research groups have made great contributions in developing both intermolecular and intramolecular aminohalogenation (Minakata et al., 2006; Li et al., 2007; Michael et al., 2008; Chen et al., 2010; Denmark et al., 2012; Yin et al., 2012; Chemler and Bovino, 2013; Song et al., 2013, 2016; Martínez and Muñiz, 2014; Broggini et al., 2015; Qin et al., 2015; Zhu et al., 2015; Legnani et al., 2018; Cai et al., 2019), in which a series of halogen/nitrogen sources, such as CFBSA (Pu et al., 2016), NCS/MeCN (Tay et al., 2013), NCP (Zhu et al., 2018), NFSI/TMSCl (Arteaga et al., 2018), TsNCl_2_ (Han et al., 2007; Wu and Wang, 2008; Wei et al., 2009), TsNHCl (Cai et al., 2011), TsNNaCl (Martínez and Muñiz, 2014), 2-NsNNaCl (Li et al., 2001), or the combination of 2-NsNCl_2_ and 2-NsNHNa (Liu et al., 2006), and so on, were employed for many types of alkene substrates. Most of these aminohalogenation systems take advantage of metals or organic catalysts to give good yields and excellent regioselectivities and diastereoselectivities. The aforementioned synthesis usually involves the use of traditional methods of purification such as column chromatography and recrystallization. Herein, in this report, we would like to present the design of new group-assisted purification (GAP) group-attached olefin substrates and their potentials for aminochlorination enabling ste minimized, greener GAP workup, and purification.

The development of greener methodologies and technologies in chemical and pharmaceutical synthesis is of utmost importance to achieve safer, faster, and economical production outcomes (Trost, 1991; Lee and Robinson, 1995; Schreiber, 2000; Tietze et al., 2008; Anderson, 2012). Recently, our group has established a concept called GAP chemistry for greener synthesis where functionalities of special interest are incorporated into the substrates to facilitate the purification of crude products based on solubility without column chromatography or recrystallization. The pure products can be readily obtained simply by washing the crude mixture with common solvents or cosolvents (Wang et al., 2013; Chennapuram et al., 2014; Dommaraju and Prajapati, 2015; Seifert et al., 2016; Patel et al., 2019). Indeed, GAP chemistry would be the first concept consisting of both chemical aspects (reagent and reaction) and physical aspects (separation and purification). The study on GAP chemistry needs to consider solubility, stability, reactivity, and other properties of GAP reagents and products. More interestingly, we have found that, for some synthesis, GAP functional groups not only can make workup and purification easier but also can increase chemical yields, sometimes resulting in quantitative yields, which is defined as group-assisted synthesis chemistry (Seifert, 2017).

A ubiquitous requirement of GAP methodology is the sufficient solubility of products, which depends on the functional groups of their reactants. The GAP-anchored products have to dissolve in polar solvents such as THF, DCM for further reactions but remain insoluble in less polar solvents such as hexane, ether, and so on. The GAP compounds should exhibit adequate reactivity toward many reactants as well. Chiral GAP groups also control asymmetric addition efficiently. Herein, we report the GAP auxiliaries, which are labile to structural modifications to control the solubility, stability, and chemical reactivity of the products. Besides, these GAP auxiliaries can be easily deprotected via reduction or hydrolysis. Group-assisted purification chemistry has thus shown its potential to revolutionize the pharmaceutical industry as it saves time, manpower, and costs in safer manners.

## Results and Discussion

**Scheme 1:**
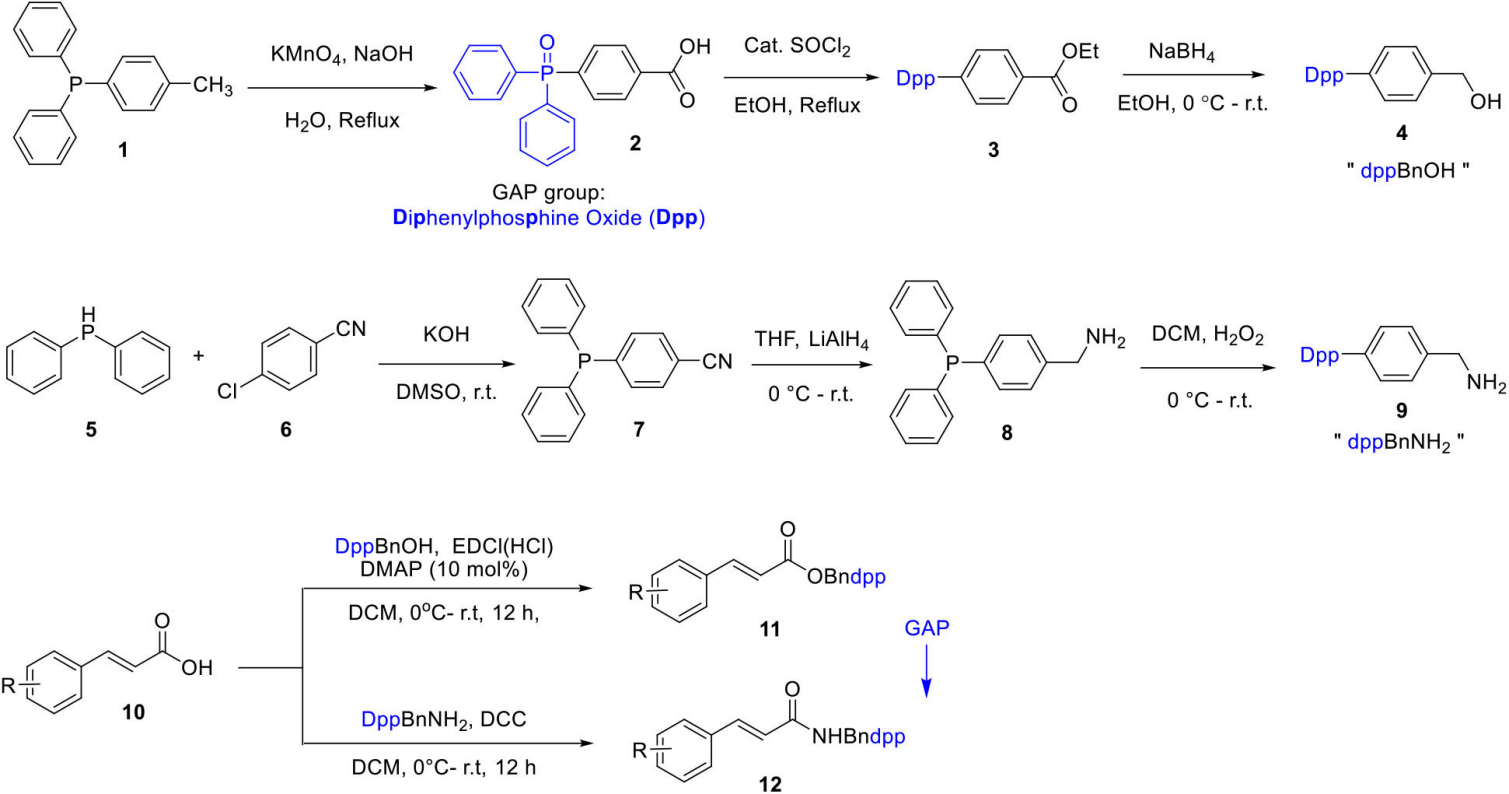
Synthesis of GAP auxiliaries for cinnamic acid protection.

We initiated the study by investigating GAP auxiliaries and their potential application in GAP chemistry. In our previous work, diphenylphosphine oxide (abbreviated as Dpp) was proven to be a stable GAP candidate. Its synthesis began with the benzylic and phosphine oxidation of commercially available, diphenyl(*p*-tolyl)phosphine **1** with potassium permanganate to benzoic acid **2**. (Scheme 1) Esterification followed by reduction with borohydride gave the first GAP-anchored benzyl alcohol **4**, or “dppBnOH,” in high yield (Seifert et al., 2016). Similarly, the condensation of diphenylphosphane **5** with 4-chlorobenzonitrile **6**, provided 4-(diphenylphosphanyl)benzonitrile **7**, which upon reduction with LiAlH_4_ resulted in amine **8**. Phosphine oxidation of the amine **8** with hydrogen peroxide afforded the second GAP-equipped benzylamine **9** or “dppBnNH_2_” (Scheme 1) (Hingst et al., 1998; Janssen et al., 2009). The protection of cinnamic acids with dppBnNH_2_ was both facile and quantitative compared to dppBnOH. The products **11** and **12** could be easily precipitated from an ethyl acetate/petroleum ether solvent mixture, thereby fulfilling the condition of GAP chemistry. The Bndpp group can be easily regained for reuse either by catalytic hydrogenation or by reduction with borohydride in 10% Pd/C.

We further investigated various reaction parameters by subjecting GAP substrates to aminohalogenation reaction. Initially, the GAP-anchored intermediate **11** was subjected to aminochlorination with dichloramine T (4-TsNCl_2_) (1.5 equiv.) in dichloromethane without any catalyst; however, no product was formed after stirring for 48 h. Then iodobenzene diacetate (PhI(OAc)_2_ and a series of transition metal catalysts were employed to no avail. Pleasingly, when 1.5 equiv. of tosylamide (4-TsNH_2_) was added along with 4-TsNCl_2_ (1.5 equiv.) in the presence of catalyst PhI(OAc)_2_ (20 mol%), the starting material **11** was consumed in 48 h at room temperature, and a single aminohalogenation product was isolated in a chemical yield of 71% (Table 1, entry 9) with a diastereoselective ratio of 8:1. To improve the yield, we again screened a variety of transition-metal compounds such as ZnCl_2_, Mn(OAc)_2_, Pd(OAc)_2_, FeCl_3_, and CuI as catalysts for this reaction. The results are depicted in Table 1, which specifies that PhI(OAc)_2_ (20 mol%) was the potent catalyst for this reaction. It was observed that Mg(OAc)_2_ gave no product; Pd(OAc)_2_, FeCl_3_, and ZnCl_2_ gave poor yields, whereas CuI and Cu(OTf)_2_ (Table 1, entry 14 and 15) gave moderately good yields. The next optimization was the addition of another 0.5 equiv. of each 4-TsNCl_2_ and 4-TsNH_2_, which increased the yield up to 76% (Table 1, entry 16). Refluxing this reaction mixture further enhanced the yield up to 83% (Table 1, entry 17). However, the yield decreased to 78% when the reaction was refluxed in the absence of 4 Å molecular sieves (Table 1, entry 18).

**Table 1:** Optimization of the reaction conditions^[a]^.

**Entry**	**Cat. (20 mol%)**	**4-TsNCl_**2**_**	**4-TsNH_**2**_**	**Yield (%)^[b]^**	**dr^[c]^**
1	–	1.5 eq	–	–	–
2	Cu(OTf)_2_	1.5 eq	–	–	–
3	FeCl_3_	1.5 eq	–	–	–
4	PhI(OAc)_2_	1.5 eq	–	–	–
5	Pd(OAc)_2_	1.5 eq	–	–	–
6	Mn(OAc)_2_	1.5 eq	–	–	–
7	CuI	1.5 eq	–	–	–
8	ZnCl_2_	1.5 eq	–	–	–
9	PhI(OAc)_2_	1.5 eq	1.5 eq	71	8:1
10	Pd(OAc)_2_	1.5 eq	1.5 eq	30	10:1
11	Mn(OAc)_2_	1.5 eq	1.5 eq	–	–
12	FeCl_3_	1.5 eq	1.5 eq	34	4:1
13	ZnCl_2_	1.5 eq	1.5 eq	24	6:1
14	CuI	1.5 eq	1.5 eq	63	10:1
15	Cu(OTf)_2_	1.5 eq	1.5 eq	61	8:1
16	PhI(OAc)_2_	2.0 eq	2.0 eq	76	8:1
17^[d]^	PhI(OAc)_2_	2.0 eq	2.0 eq	83	8:1
18^[e]^	PhI(OAc)_2_	2.0 eq	2.0 eq	78	8:1

[a] *Unless otherwise specified, all reactions were performed with 0.15 mmol of **11**, 20 mol% of the catalyst, 750 mg of MS 4 Å in 1.5 mL of CH_2_Cl_2_ at room temperature under Ar*.

[b] *Isolated yields with GAP washing (for 10, 12, and 13, GAP washing was not conducted)*.

[c] *The dr values were determined by the analysis of ^1^H and ^31^P NMR spectra*.

[d] *The reaction was performed at reflux*.

[e] *The reaction was performed in the absence of 4 Å molecular sieves at reflux*.

Using 20 mol% of PhI(OAc)_2_ as the catalyst with 2 equiv. of 4-TsNCl_2_ and 4-TsNH_2_ at reflux temperature, various solvents such as CHCl_3_, CH_2_Cl_2_, MeCN, THF, Et_2_O, and toluene were screened, which revealed that CH_2_Cl_2_ was found to be the best solvent to give **13** in 83% yield with good stereoselectivity (8:1, Table 2, entries 1 and 12). Moderate yields were obtained in MeCN and DCE solvents. The product **13** was obtained only with great difficulty in toluene; no reaction was observed in THF, Et_2_O, DMF, and MeOH, whereas only traces were formed in dioxane solvent. A decrease in the reaction time by 24 h led to slightly reduced yield (77%) of the product **13** (Table 2, entry 11); however, the yield did not change with the prolonged reaction time of 72 h (83%, Table 2, entry 12). Finally, we noticed that 20 mol% of PhI(OAc)_2_ was essential for the reaction as the yield decreased to 27% when 10 mol% of the catalyst was used in the system (Table 2, entry 13).

**Table 2:** Further optimization^[a]^.

**Entry**	**Solvent**	**Time (h)**	**x**	**Yield (%)^[b]^**	**dr^[c]^**
1	CH_2_Cl_2_	48	20	83	8:1
2	CHCl_3_	48	20	80	8:1
3	CH_3_CN	48	20	50	10:1
4	PhMe	48	20	24	7:1
5	Et_2_O	48	20	–	–
6	THF	48	20	–	–
7	DCE	48	20	48	4:1
8	DMF	48	20	–	–
9	MeOH	48	20	–	–
10	Dioxane	48	20	Traces	–
11	CH_2_Cl_2_	24	20	77	8:1
12	CH_2_Cl_2_	72	20	83	8:1
13	CH_2_Cl_2_	48	10	27%	8:1

[a] *Unless otherwise specified, all reactions were performed with 0.15 mmol of **11**, 0.3 mmol of 4-TsNCl_2_, 0.3 mmol of 4-TsNH_2_, 750 mg of MS 4Å in 1.5 mL of solvent at reflux under Ar*.

[b] *Isolated yields with GAP washing (for 4 and 13, GAP washing was not conducted)*.

[c] *The dr values were determined by the analysis of ^1^H and ^31^P NMR spectra*.

**Scheme 2:**
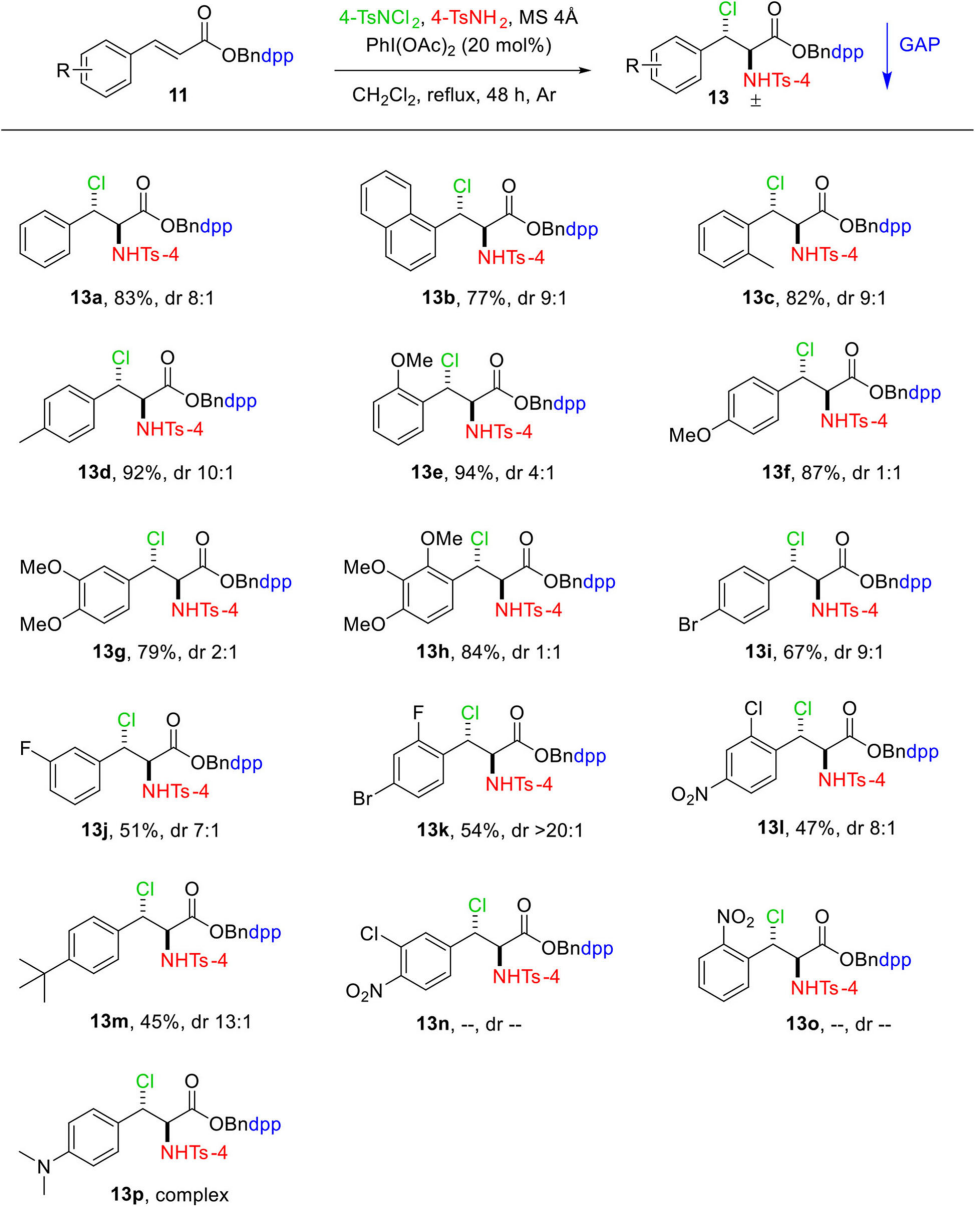
Substrate scope of aminochlorination of *N*-(4-(diphenylphosphoryl)benzyl) cinnamates. Unless otherwise specified, all reactions were performed with 0.15 mmol of **11**, 0.3 mmol of 4-TsNCl_2_, 0.3 mmol of 4-TsNH_2_, 750 mg of MS 4Å in 1.5 mL of DCM at reflux under Ar. The dr values were determined by the analysis of ^1^H and ^31^P NMR spectra. Isolated yields with GAP washing.

With the optimized reaction parameters, the substrate scope of this reaction was thereafter explored with a variety of GAP auxiliary **4** anchored substituted cinnamic acids **11**. The results are shown in Scheme 2. Pleasantly, a wide range of functional groups tolerance was observed on the aromatic ring of cinnamic acids including electron-donating groups **13c**–**13h** and electron-withdrawing groups **13i**–**13o**, which provided moderate to high yields (45–94%). As shown in Scheme 2, for those substrates **11** bearing electron-donating groups on the aromatic ring of cinnamic acid, the addition reaction went cleanly to produce the relevant adducts **13** in good yields (Scheme 2, **13c−13h**). The highest yield of 94% was obtained for **13e** where the aromatic ring has strong electron-donating group MeO at the *ortho*-position. On the other hand, substrates **11** bearing electron-withdrawing groups such as F, Cl, Br, or NO_2_ on the aromatic rings gave the resultant products in lower yields under the same conditions (Scheme 2, **13i−13o**). The lowest yield of 45% was obtained for **13m**, which has a tertiary butyl group at the *para-*position. Substrates **13n** and **13o** bearing strong electron-withdrawing groups such as NO_2_ and Cl did not undergo reaction, whereas substrate **13p** with *N,N*-dimethylamine group at *para-*position resulted in a complex mixture with traces of desired product.

A major objective of this project was the development of GAP auxiliaries, which could potentially simplify the purification of the haloamine products. Thus, the cinnamic acids **10** were protected with another GAP auxiliary DppBnNH_2_ **9** to afford *N*-(4-(diphenylphosphoryl)benzyl) cinnamamides **12**, (Scheme 1), which were then exposed to aminochlorination reaction. Conditions for this transformation were also optimized (Table 1S, Supporting Information). We found that 20 mol% of PhI(OAc)_2_, 2.0 equiv. of 4-TsNH_2_ and 4-TsNCl_2_, was essential to obtain haloamine product **14a** in 73% yield with diastereoselective ratio of 10:1 when refluxed in dichloromethane for 48 h.

**Scheme 3:**
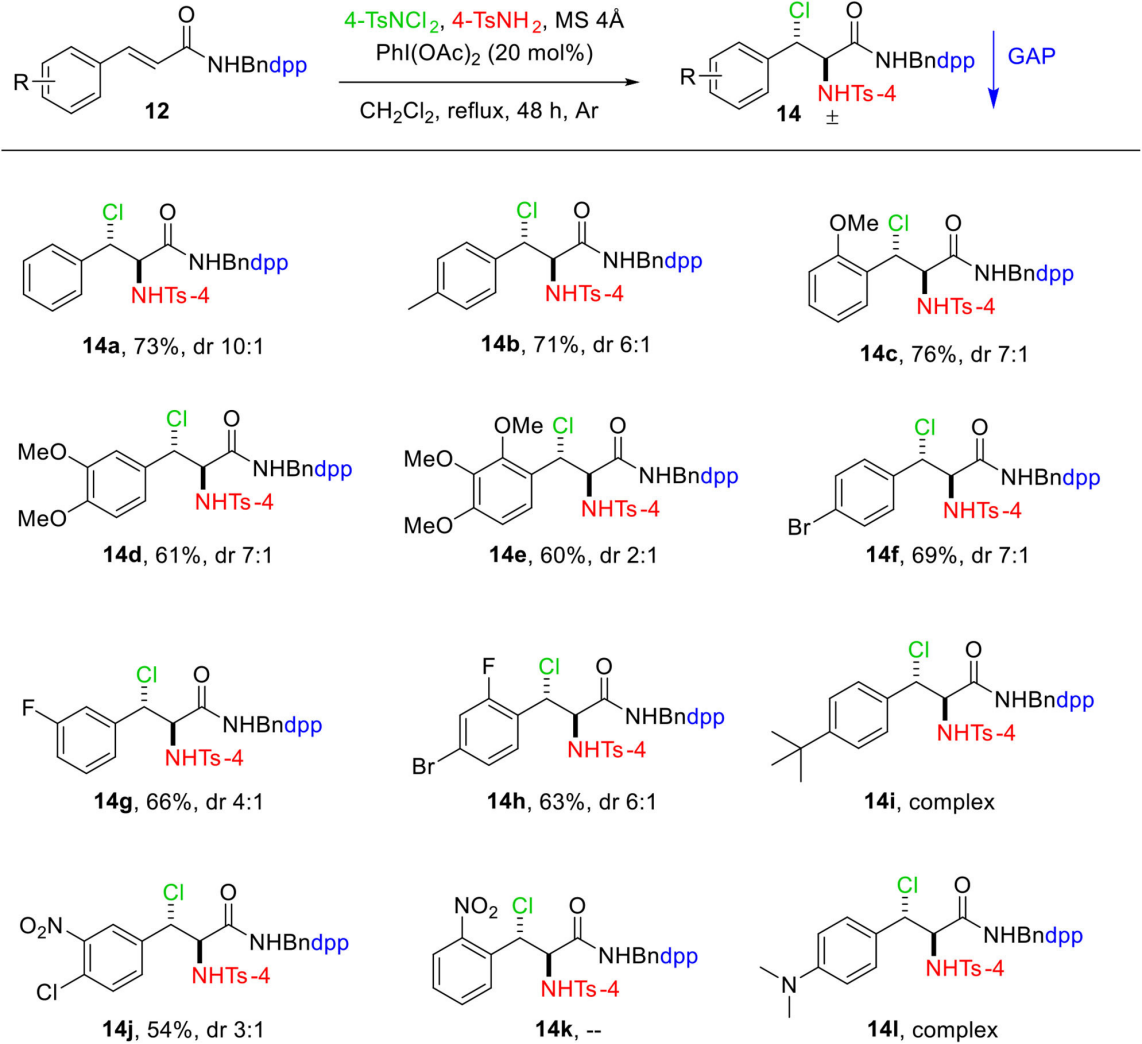
Substrate scope of aminochlorination of *N*-(4-(diphenylphosphoryl)benzyl) cinnamamides. Unless otherwise specified, all reactions were performed with 0.15 mmol of **12**, 0.3 mmol of 4-TsNCl_2_, 0.3 mmol of 4-TsNH_2_, 750 mg of MS 4Å in 1.5 mL of DCM at reflux under Ar. The dr values were determined by the analysis of ^1^H and ^31^P NMR spectra. Isolated yields with GAP washing.

We also investigated the scope of this transformation by using a variety of *N*-(4-(diphenylphosphoryl)benzyl) cinnamamides **12**. As illustrated in Scheme 3, the reaction tolerated a wide range of functional groups to provide moderate to high yields (54%−76%). For the substrate having methoxy group at *ortho*-position on aromatic ring, the highest yield of 76% was obtained (Scheme 3, **14c**). The nitro group, on the other hand, lowers the yield considerably under the same conditions (Scheme 3, **14j**). The substrate with NO_2_ group at *ortho*-position of aromatic ring **14k** did not undergo reaction even after 72 h, whereas the reactions for the products **14i** and **14l** were sluggish and resulted in a complex mixture.

**Scheme 4:**
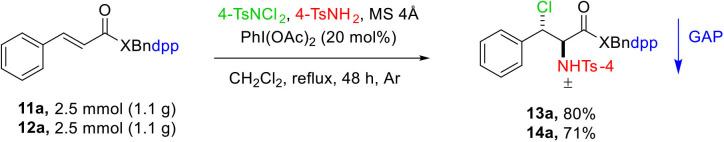
Gram scale reaction.

The practicality of this protocol was determined by performing the reaction on gram scale for the starting material **11a** and **12a**, which led to the formation of the products **13a** and **14a** in 80% and 71% yields, respectively (Scheme 4).

**Scheme 5:**
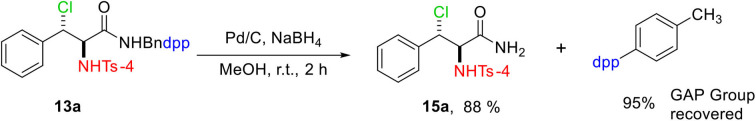
Group-assisted purification deprotection.

The GAP-tailored vicinal chloramine was deprotected in the presence of Pd/C and NaBH_4_ which afforded Bndpp in 95% yield (Scheme 5). For the purification of the products, the mixture is dissolved in minimal amount of a polar solvent such as ethyl acetate or DCM, and then petroleum ether is added. The GAP auxiliary precipitates in the form of a white solid, which is filtered and washed with petroleum ether. The filtrate is evaporated under vacuum to obtain the desired β-chloroamine as a white product.

## Mechanism

**Scheme 6:**
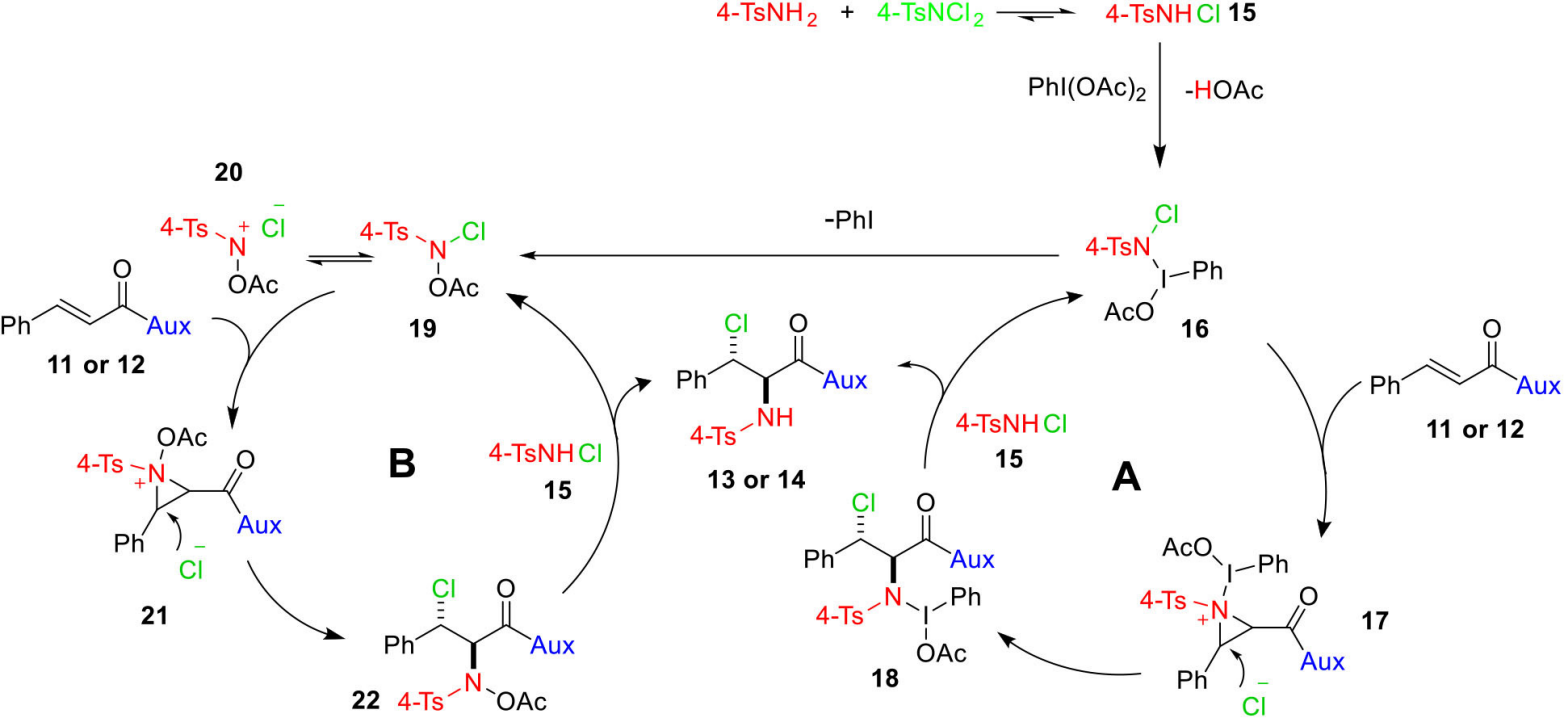
A plausible mechanism for the syntheses of vicinal chloramines.

Based on experimental observations and our as well as others' previous works (Li et al., 2001; Wei et al., 2001; Wang and Wu, 2007; Wu and Wang, 2008; Chen et al., 2009), a rational mechanism for the formation of **13** and **14** is illustrated in Scheme 6. 4-TsNCl_2_ on reaction with 4-TsNH_2_ may produce *N*-chloro-*p*-toluenesulfonamide (4-TsNHCl) **15**, which might be oxidized by PhI(OAc)_2_ to generate intermediate **16** that may either follow cycle A or cycle B. The intermediate **16**, in cycle A, could form aziridinium **17** with the double bond of **11** or **12**, which in turn is attacked by the dissociated halogen anion from the intermediate **16** at the more electrophilic carbon (beta to carbonyl carbon) to yield compound **18** stereoselectively. Eventually, intermediate **18** together with 4-TsNHCl **15** provides the uttermost haloamine product **13** or **14** and restores intermediate **16**. There is a possibility of formation of *N*-acetoxy-*N*-halo-*p*-toluenesulfonamide **19** when phenyl iodide is released as a result of dissociation of the unstable N–I bond of intermediate **16**, which could then be the active intermediate for cycle B. Species **19** that forms an equilibrium with nitrenium ion intermediate **20** (Kikugawa et al., 2003; Murata et al., 2008) could react with olefin **11** or **12** to afford aziridinium intermediate **21**, which would lead to intermediate **22** following an S_N_2 nucleophilic attack by the nearby halide anion. Finally, the reaction of the intermediate **22** with 4-TsNHCl gives the final product and regenerate intermediate **19**.

## Experimental Section

### Aminochlorination of 4-(Diphenylphosphoryl)Benzyl Cinnamates 13 and *N*-(4-(Diphenylphosphoryl)Benzyl) Cinnamamides 14

Typical procedure: Into a dry vial, 11 or 12 (1.5 mmol, 1 eq), 4-TsNCl_2_ (3 mmol, 2 eq) 4-TsNH_2_ (3 mmol, 2 eq), PhI(OAc)_2_ (20 mol%), and freshly activated 4 Å molecular sieves (150 mg) were added. The resulting mixture was capped under argon protection. Anhydrous DCM (3 mL) was injected through a syringe and was refluxed for 48 h. After completion (monitored by TLC), the reaction was quenched with saturated aqueous Na_2_SO_3_ solution (2 mL), and DCM (3 × 10 mL) was added to extract the product. The combined organic layers were washed with brine, dried over anhydrous sodium sulfate, and evaporated to dryness *in vacuo*. The mixture is dissolved in minimal amount of a polar solvent such as ethyl acetate or DCM, and then petroleum ether is added. The GAP auxiliary precipitates in the form of a white solid, which is filtered and washed with petroleum ether. The filtrate is evaporated under vacuum to obtain the desired β-chloroamine as a white product.

### General Procedure for Deprotection of GAP Auxiliary BnDpp

To a stirred solution of **13a** or **14a** (0.1 g, 0.16 mmol) and 10 wt% Pd/C (10 mg) in MeOH (1 mL), NaBH_4_ (7.6 mg, 2 equiv.) was added. The 10-mL flask was closed with a rubber septum with an empty (deflated) balloon to avoid the loss of generated hydrogen and overpressure in the flask. After 2 h, the reaction mixture was filtered through Celite, and filtrate was evaporated to dryness and redissolved in EtOAc. Then, the reaction mixture was neutralized with KHSO_4_. The organic layer was separated, dried over anhydrous Na_2_SO_4_, and evaporated to dryness to afford crude GAP auxiliary, which was easily purified using the GAP washing method.

## Conclusion

A highly efficient regioselective and stereoselective aminochlorination reaction of electron-deficient olefins anchored with GAP auxiliaries dppBnOH and dppBnNH_2_ catalyzed by PhI(OAc)_2_ in dichloromethane with 4-TsNH_2_ and 4-TsNCl_2_ as the nitrogen and chlorine sources has been developed. Moderate to good chemical yields and excellent regioselectivity and stereoselectivity have been obtained. The GAP approach has been effectively implemented, which bypasses column chromatography and recrystallization. Pure products have been obtained simply by washing the crude mixtures with inexpensive petroleum solvents or cosolvents to give good to high yields. Besides, the GAP auxiliary is recyclable and reusable.

## Data Availability Statement

The raw data supporting the conclusions of this article will be made available by the authors, without undue reservation.

## Author Contributions

GL and AR designed the project. AR, NZ, and WY performed the experiments. AA and PZ analyzed the data. AR, AA, and GL wrote the manuscript. All authors contributed to the article and approved the submitted version.

## Conflict of Interest

The authors declare that the research was conducted in the absence of any commercial or financial relationships that could be construed as a potential conflict of interest.

## References

[B1] Anderson N. G. (2012). Practical Process Research and Development: A Guide for Organic Chemists. Cambridge, UK: Academic Press.

[B2] Arteaga G. C. Saavedra-Olavarría J. Almendras S. Hermosilla-Ibáñez P. Almodovar I. Pérez E. G. (2018). Copper-catalyzed intermolecular aminochlorination of alkenes. Tetrahedron Lett. 59, 1091–1093. 10.1016/j.tetlet.2018.02.010

[B3] Bovino M. T. Chemler S. R. (2012). Catalytic enantioselective alkene aminohalogenation/cyclization involving atom transfer. Angew. Chem. Int. Ed. Engl. 51, 3923–3927. 10.1002/anie.201109044 22392873 PMC3324620

[B4] Broggini G. Beccalli E. M. Borelli T. Brusa F. Gazzola S. Mazza A. (2015). Intra-intermolecular palladium-catalyzed domino reactions of glycine allylamides for the synthesis of diversely functionalized piperazinones. Eur. J. Org. Chem. 2015, 4261–4268. 10.1002/ejoc.201500386

[B5] Cai Y. Liu X. Jiang J. Chen W. Lin L. Feng X. (2011). Catalytic asymmetric chloroamination reaction of α,β-unsaturated γ-keto esters and chalcones. J. Am. Chem. Soc. 133, 5636–5639. 10.1021/ja110668c 21443187

[B6] Cai Y. Liu X. Zhou P. Feng X. (2019). Asymmetric catalytic halofunctionalization of α,β-unsaturated carbonyl compounds. J. Org. Chem. 84, 1–13. 10.1021/acs.joc.8b01951 30339377

[B7] Chemler S. R. Bovino M. T. (2013). Catalytic aminohalogenation of alkenes and alkynes. ACS Catal 3, 1076–1091. 10.1021/cs400138b 23828735 PMC3697159

[B8] Chen D. Guo L. Liu J. Kirtane S. Cannon J. F. Li G. (2005). Functionalization of alpha,beta-unsaturated esters and ketones: a facile and highly stereoselective one-pot approach to *N*-protected alpha,beta-dehydroamino acid derivatives. Org. Lett. 7, 921–924. 10.1021/ol050002u 15727475

[B9] Chen Z.-G. Wang Y. Wei J.-F. Zhao P.-F. Shi X.-Y. (2010). K_3_PO_4_-catalyzed regiospecific aminobromination of β-nitrostyrene derivatives with *N*-bromoacetamide as aminobrominating agent. J. Org. Chem. 75, 2085–2088. 10.1021/jo9026879 20170090

[B10] Chen Z.-G. Wei J.-F. Wang M.-Z. Zhou L.-Y. Zhang C.-J. Shi X.-Y. (2009). Aluminium powder-catalyzed regio- and stereoselective aminobromination of α,β-unsaturated carbonyl compounds and simple olefins with the *p*-toluenesulfonamide/ *N*-bromosuccinimide (TsNH_2_-NBS) system. Adv. Synth. Catal. 351, 2358–2368. 10.1002/adsc.200900343

[B11] Chennapuram M. Emmadi N. R. Bingi C. Nanubolu J. B. Atmakur K. (2014). Group-assisted purification (GAP) chemistry for dihydrofurans: water as a medium for catalyst free synthesis in a one pot four component reaction. Green Chem. 16, 3237–3246. 10.1039/c4gc00388h

[B12] Denmark S. E. Kuester W. E. Burk M. T. (2012). Catalytic, asymmetric halofunctionalization of alkenes—a critical perspective. Angew. Chem. Int. Ed. Engl. 51, 10938–10953. 10.1002/anie.201204347 23011853 PMC3529098

[B13] Dommaraju Y. Prajapati D. (2015). A highly efficient group-assisted purification method for the synthesis of poly-functionalized pyrimidin-5-yl-pyrroles via one-pot four-component domino reaction. Mol. Divers. 19, 173–187. 10.1007/s11030-014-9547-1 25173493

[B14] Ghorai M. K. Sahoo A. K. Kumar S. (2011). Synthetic route to chiral tetrahydroquinoxalines via ring-opening of activated aziridines. Org. Lett. 13, 5972–5975. 10.1021/ol2023906 22004011

[B15] Han J.-L. Zhi S.-J. Wang L.-Y. Pan Y. Li G. (2007). CuCl-catalyzed regio- and stereoselective aminohalogenation of α,β-unsaturated nitriles. Eur. J. Org. Chem. 2007, 1332–1337. 10.1002/ejoc.200600902

[B16] Hingst M. Tepper M. Stelzer O. (1998). Nucleophilic phosphanylation of fluoroaromatic compounds with carboxyl, carboxymethyl, and aminomethyl functionalities – an efficient synthetic route to amphiphilic arylphosphanes. Eur. J. Org. Chem. 1998, 73–82.

[B17] Janssen M. Müller C. Vogt D. (2009). ‘Click' dendritic phosphines: design, synthesis, application in suzuki coupling, and recycling by nanofiltration. Adv. Synth. Catal. 351, 313–318. 10.1002/adsc.200900058

[B18] Kikugawa Y. Nagashima A. Sakamoto T. Miyazawa E. Shiiya M. (2003). Intramolecular cyclization with nitrenium ions generated by treatment of N-acylaminophthalimides with hypervalent iodine compounds: formation of lactams and spiro-fused lactams. J. Org. Chem. 68, 6739–6744. 10.1021/jo0347009 12919042

[B19] Lee S. A. Robinson G. (1995). Process Development: Fine Chemicals From Grams to Kilograms. Oxford, UK: Oxford University Press Oxford.

[B20] Legnani L. Prina-Cerai G. Delcaillau T. Willems S. Morandi B. (2018). Efficient access to unprotected primary amines by iron-catalyzed aminochlorination of alkenes. Science 362, 434–439. 10.1126/science.aat3863 30361368

[B21] Li G. Kotti S. R. S. S. Timmons C. (2007). Recent development of regio- and stereoselective aminohalogenation reaction of alkenes. Eur. J. Org. Chem. 2007, 2745–2758. 10.1002/ejoc.200600990

[B22] Li G. Wei H.-X. Kim S. H. (2001). Unexpected copper-catalyzed aminohalogenation reaction of olefins using *N*-halo-*N*-metallo-sulfonamide as the nitrogen and halogen sources. Tetrahedron 57, 8407–8411. 10.1016/S0040-4020(01)00847-X

[B23] Liu J. Wang Y. Li G. (2006). Regio- and stereoselective synthesis of anti-1,3-diaryl-3-chloro- 2-(o-nitrophenylsulfonylamino)-3-propan-1-ones through catalytic aminohalogenation reaction of α,β-unsaturated ketones. Eur. J. Org. Chem. 2006, 3112–3115. 10.1002/ejoc.200600020

[B24] Martínez C. Muñiz K. (2014). A versatile metal-free intermolecular aminochlorination of alkenes. Adv. Synth. Catal. 356, 205–211. 10.1002/adsc.201300880

[B25] Michael F. E. Sibbald P. A. Cochran B. M. (2008). Palladium-catalyzed intramolecular chloroamination of alkenes. Org. Lett. 10, 793–796. 10.1021/ol702922c 18247496

[B26] Minakata S. Yoneda Y. Oderaotoshi Y. Komatsu M. (2006). Unprecedented CO_2_-promoted aminochlorination of olefins with chloramine-T. Org. Lett. 8, 967–969. 10.1021/ol0600178 16494486

[B27] Murata K. Tsukamoto M. Sakamoto T. Saito S. Kikugawa Y. (2008). Hydrazidohydroxylation of styrenes with *N*-acetylaminophthalimide using phenyliodine(III) bis(trifluoroacetate) (PIFA). Synthesis 2008, 32–38. 10.1055/s-2007-1000819

[B28] Patel D. M. Vala R. M. Sharma M. G. Rajani D. P. Patel H. M. (2019). A practical green visit to the functionalized [1,2,4]Triazolo[5,1-*b*]quinazolin-8(4*H*)one scaffolds using the group-assisted purification (GAP) chemistry and their pharmacological testing. Chemistry Select 4, 1031–1041. 10.1002/slct.201803605

[B29] Pu X.-Q. Zhao H.-Y. Lu Z.-H. He X.-P. Yang X.-J. (2016). Aminochlorination of alkenes with CFBSA. Eur. J. Org. Chem. 2016, 4526–4533. 10.1002/ejoc.201600709

[B30] Qin Q. Ren D. Yu S. (2015). Visible-light-promoted chloramination of olefins with *N*-chlorosulfonamide as both nitrogen and chlorine sources. Org. Biomol. Chem. 13, 10295–10298. 10.1039/C5OB01725D 26416235

[B31] Schreiber S. L. (2000). Target-oriented and diversity-oriented organic synthesis in drug discovery. Science 287, 1964–1969. 10.1126/science.287.5460.1964 10720315

[B32] Schröder S. P. van de Sande J. W. Kallemeijn W. W. Kuo C.-L. Artola M. van Rooden E. J.. (2017). Towards broad spectrum activity-based glycosidase probes: synthesis and evaluation of deoxygenated cyclophellitol aziridines. Chem. Commun. 53, 12528–12531. 10.1039/C7CC07730K 29116266

[B33] Seifert C. W. (2017). New synthetic methodology for chiral amines and peptides via GAP technology (Ph.D. thesis). Texas Tech University.

[B34] Seifert C. W. Paniagua A. White G. A. Cai L. Li G. (2016). GAP Peptide Synthesis via Design of New GAP Protecting Group: An Fmoc/tBu Synthesis of Thymopentin Free from Polymers, Chromatography and Recrystallization. Eur. J. Org. Chem. 2016, 1714–1719. 10.1002/ejoc.201600026 28663711 PMC5486986

[B35] Song L. Luo S. Cheng J.-P. (2013). Catalytic intermolecular haloamidation of simple alkenes with *N*-halophthalimide as both nitrogen and halogen source. Org. Lett. 15, 5702–5705. 10.1021/ol402726d 24156477

[B36] Song L. Luo S. Cheng J.-P. (2016). Visible-light promoted intermolecular halofunctionalization of alkenes with *N*-halogen saccharins. Org. Chem. Front. 3, 447–452. 10.1039/C5QO00432B

[B37] Tay D. W. Tsoi I. T. Er J. C. Leung G. Y. C. Yeung Y.-Y. (2013). Lewis basic selenium catalyzed chloroamidation of olefins using nitriles as the nucleophiles. Org. Lett. 15, 1310–1313. 10.1021/ol400249x 23461531

[B38] Thakur R. Rawal G. K. Vankar Y. D. (2017). Synthesis of chiral aziridines from glycals: application in the synthesis of a piperidine–azepine fused derivative. Eur. J. Org. Chem. 2017, 4235–4241. 10.1002/ejoc.201700624

[B39] Thakur V. V. Talluri S. K. Sudalai A. (2003). Transition metal-catalyzed regio- and stereoselective aminobromination of olefins with TsNH_2_ and NBS as nitrogen and bromine sources. Org. Lett. 5, 861–864. 10.1021/ol027530f 12633091

[B40] Tietze L. Brasche G. Gericke K. (2008). Domino Reactions in Organic Synthesis 2006. Weinheim: Wiley-VCH 10.1002/9783527609925

[B41] Trost B. M. (1991). The atom economy–a search for synthetic efficiency. Science 254, 1471–1477. 10.1126/science.1962206 1962206

[B42] Van T. N. De Kimpe N. (2000). Synthesis of chiral *cis*-1,2,3-trisubstituted aziridines. Tetrahedron 56, 7299–7304. 10.1016/S0040-4020(00)00627-X

[B43] Wang G.-W. Wu X.-L. (2007). Mechanochemical aminochlorination of electron-deficient olefins with chloramine-T promoted by (Diacetoxyiodo)benzene. Adv. Synth. Catal. 349, 1977–1982. 10.1002/adsc.200700020

[B44] Wang H. Liu X. Feng X. Huang Z. Shi D. (2013). GAP chemistry for pyrrolyl coumarin derivatives: a highly efficient one-pot synthesis under catalyst-free conditions. Green Chem. 15, 3307–3311. 10.1039/c3gc41799a

[B45] Wei H.-X. Kim S. H. Li G. (2001). The first transition metal–ligand complex-catalyzed regioselective and stereoselective aminohalogenation of cinnamic esters. Tetrahedron 57, 3869–3873. 10.1016/S0040-4020(01)00228-9

[B46] Wei J.-F. Zhang L.-H. Chen Z.-G. Shi X.-Y. Cao J.-J. (2009). KI-catalyzed aminobromination of olefins with TsNH_2_-NBS combination. Org. Biomol. Chem. 7, 3280–3284. 10.1039/b904789a 19641786

[B47] Wu X.-L. Wang G.-W. (2008). Aminohalogenation of electron-deficient olefins promoted by hypervalent iodine compounds. Eur. J. Org. Chem. 2008, 6239–6246. 10.1002/ejoc.200800842

[B48] Xiong Y. Qian P. Cao C. Mei H. Han J. Li G.. (2014). One-pot stereoselective synthesis of α,β-differentiated diamino esters via the sequence of aminochlorination, aziridination and intermolecular S_N_2 reaction. Beilstein J. Org. Chem. 10, 1802–1807. 10.3762/bjoc.10.189 25161740 PMC4142878

[B49] Yin G. Wu T. Liu G. (2012). Highly selective palladium-catalyzed intramolecular chloroamination of unactivated alkenes by using hydrogen peroxide as an oxidant. Chem. A Euro. J. 18, 451–455. 10.1002/chem.201102776 22162296

[B50] Zhu C.-L. Tian J.-S. Gu Z.-Y. Xing G.-W. Xu H. (2015). Iron(II)-catalyzed asymmetric intramolecular olefin aminochlorination using chloride ion. Chem. Sci. 6, 3044–3050. 10.1039/C5SC00221D 26807211 PMC4719767

[B51] Zhu N. Li Y. Bao H. (2018). Metal-free intermolecular aminochlorination of unactivated alkenes. Org. Chem. Front. 5, 1303–1307. 10.1039/C8QO00001H

