# Supplementary material for: Hypervalent Iodine (III) Catalyzed Regio- and Diastereoselective Aminochlorination of Tailored Electron Deficient Olefins via GAP Chemistry
Source: Front Chem. 2020 Jul 7;8:523. doi: 10.3389/fchem.2020.00523 (PMC7358771; doi:10.3389/fchem.2020.00523)
Supplement: Supplementary file 1 [file Data_Sheet_1.docx]

***Supporting Information***

Hypervalent Iodine (III) Catalyzed Regio- and Diastereoselective Aminochlorination of Tailored Electron-Deficient Olefins *via* GAP Chemistry

Anis Ur Rahman,^1^ Nighat Zarshad,^2^ Peng Zhou,^1^ Weitao Yang,^1^ Guigen Li *^1,3^ and Asad Ali*^4^

^1^ Institute of Chemistry and BioMedical Sciences, School of Chemistry and Chemical Engineering, Nanjing University, Nanjing, 210093, China

^2^ School of Chemistry and Chemical Engineering, Southeast University, Nanjing, 211189, China

^3^ Department of Chemistry and Biochemistry, Texas Tech University, Lubbock, Texas 79409-1061, United States

^4^ Faculty of Chemical and Life Sciences, Department of Chemistry, Abdul Wali Khan University, Mardan, 23200, Pakistan.

**Table of contents**

[1. General Methods S2](#_Toc20436652)

2. General procedures for the preparation of starting materials S2

2.1. Synthesis and characterization of GAP auxiliary (4-(hydroxymethyl)phenyl) diphenylphosphine oxide (dppBnOH) (4) S2

2.2. Synthesis and characterization of GAP auxiliary (4-(aminomethyl)phenyl)diphenyl phosphine oxide (dppBnNH_2_) (9) S4

2.3. General procedure for the preparation of 4-(diphenylphosphoryl)benzyl cinnamates (11) S6

2.4. General procedure for the preparation of 4-(diphenylphosphoryl)benzyl cinnamides (12) S14

3. Aminochlorination of 4-(diphenylphosphoryl)benzyl cinnamates (13) S21

4. Aminochlorination of *N*-(4-(diphenylphosphoryl)benzyl)cinnamamides (14) S29

5. General procedure for deprotection of GAP auxiliary Bndpp S37

6. NMR Spectra S38

References S71

# General Methods

Unless otherwise stated, all commercial reagents and solvents were used without additional purification. Visualization on TLC was achieved using UV light (254 nm). ^1^H NMR was recorded on Bruker Avance 400 (400 MHz). ^13^C NMR was recorded on Bruker Avance 400 (100 MHz). ^31^P NMR was recorded on Bruker Avance 400 (162 MHz). Chemical shifts were quoted in parts per million (ppm) referenced to the appropriate solvent peak or 0.0 ppm for tetramethylsilane. The following abbreviations were used to describe peak splitting patterns when appropriate: s (singlet), d (doublet), t (triplet), q (quartet), dd (doublet of doublet), td (triplet of doublet), ddd (doublet of doublet of doublet), m (multiplet). Coupling constants, *J*, were reported in hertz unit (Hz). HRMS analyses were carried out using a TOF-MS instrument with an ESI source.

# General procedures for the preparation of starting materials

## Synthesis and characterization of GAP auxiliary (4-(hydroxymethyl)phenyl) diphenylphosphine oxide (dppBnOH) (4)

**Scheme S-1:** General procedure for the synthesis of dppBnOH **(4).**

*4-(diphenylphosphoryl)benzoic acid* ***(2)***

Following a procedure by Cole W. Seifert et al., ^1^ diphenyl(*p*-tolyl)phosphane **1** (10 g, 36 mmol) was placed in a 500 mL round-bottomed flask, followed by NaOH (aq) (0.43 M, 130 mL) and then KMnO_4_ (22.2 g, 140 mmol). The reaction was stirred and heated at reflux for 12 h, after which the reaction mixture was filtered through Celite while hot. The resulting solution was washed twice with diethyl ether, followed by the addition of 50 % H_2_SO_4_ to precipitate the product. After filtration, 4-(diphenylphosphoryl)benzoic acid **2** (10.8 g, 93 %) was collected as a white solid that was directly subjected to the next reaction.

*Ethyl 4-(diphenylphosphoryl)benzoate* ***(3)***^2^

4-(diphenylphosphoryl)benzoic acid **2** (10.6 g, 33 mmol) was placed in a 500 mL round-bottomed flask along with EtOH (300 mL) and SOCl_2_ (3 mL). The reaction was heated to reflux and stirred for 12 h. Upon completion, the reaction was cooled to room temperature, the solvent was evaporated and the residue obtained was purified by column chromatography (DCM/MeOH 3%) to give ethyl 4-(diphenylphosphoryl)benzoate **3** (9.0 g, 78 %) as a dense oil.

*(4-(hydroxymethyl)phenyl)diphenylphosphine oxide* ***(4)****^1^*

Ester **3** (9.0 g, 25.7mmol) was placed in a 500 mL round-bottomed flask along with ethanol (300 mL). The reaction was cooled to 0 °C, after which NaBH_4_ (2.9 g, 77 mmol) was added portion wise. The reaction was brought to room temperature and stirred for 12 h. The solvent was evacuated, followed by solvation of the crude material in DCM and washing three times with 2 M HCl (aq). The organic layer was then dried with Na_2_SO_4_, filtered, and evacuated to afford (4-(hydroxymethyl)phenyl)diphenylphosphine oxide (BndppOH) **4** as a white solid which was subsequently washed with ether to remove ester impurities. Yield 86% (6.8 g). ^1^H NMR (400 MHz, Chloroform-*d*) δ 7.60 – 7.51 (m, 4H), 7.51 – 7.29 (m, 10H), 5.08 (t, *J* = 6.0 Hz, 1H), 4.64 (d, *J* = 5.3 Hz, 2H). ^13^C NMR (101 MHz, CDCl_3_) δ 146.74, 146.71, 132.74, 132.05, 132.03, 132.00, 131.95, 131.94, 131.70, 130.59, 129.54, 128.58, 128.45, 126.65, 126.53, 63.89.

## Synthesis and characterization of GAP auxiliary (4-(aminomethyl)phenyl)diphenyl phosphine oxide (dppBnNH_2_) (9)

**Scheme S 2:** General procedure for the synthesis of dppBnNH_2_ **(9).**

*4-(diphenylphosphanyl)benzonitrile (****7****)* ***^3^***

Diphenylphosphane **5** (10.5 mL, 60 mmol) was added to a suspension of KOH (6 g, 108 mmol) in DMSO (300 mL) and the mixture were stirred for 2 h at RT. After addition of 4‑chlorobenzonitrile **6** (8.25 g, 60 mmol) the mixture was stirred for 1 h at 60 °C. Crude product was precipitated from the reaction mixture by addition of water and separated by filtration. Further purification was performed by recrystallization from MeOH to give white crystals 80% (13.8 g). ^1^H NMR (400 MHz, Chloroform-*d*) δ 7.57 (dd, *J* = 8.2, 1.5 Hz, 2H), 7.44 – 7.27 (m, 12H). ^31^P NMR (162 MHz, CDCl_3_) δ -4.28.

*(4-(diphenylphosphanyl)phenyl)methanamine (****8)***

Following the procedure of Janssen, M et al.,^4^ to a solution of phosphinobenzonitrile **7** (13.8 g, 48 mmol) in THF (300 mL) at 0°C was added LiAlH_4_ (7.3 g, 192 mmol) in small portions. The mixture was stirred for 2h at 50°C and after cooling to 0 °C, diluted with diethyl ether (150 mL). The reaction mixture was then quenched by careful addition of water and 15% aqueous sodium hydroxide (10 mL). The resultant slurry was filtered through a Celite pad, the phases in the filtrate were separated; the organic phase was dried over Na_2_SO_4_ and concentrated to dryness. The crude product was purified via column chromatography over silica using CHCl_3_/MeOH (9:1) as an eluent to afford yellowish oil 87% (12.2 g). ^1^H NMR (400 MHz, Chloroform-*d*) δ 7.35 – 7.28 (m, 12H), 3.87 (s, 2H), 1.71 (s, 2H). ^31^P NMR (162 MHz, CDCl_3_) δ -6.04. ^13^C NMR (101 MHz, CDCl_3_) δ 143.96, 137.45, 137.34, 135.53, 135.42, 134.23, 134.03, 133.80, 133.60, 128.71, 128.56, 128.52, 128.49, 127.36, 127.29, 46.21.

*(4-(aminomethyl)phenyl)diphenylphosphine oxide* ***(9)* ^4^**

The phosphinobenzylamine (12.2 g, 42 mmol) was dissolved in 250 mL of CH_2_Cl_2_ and H_2_O_2_ (17 mL, 35% solution in water) was added at 0°C. After 1h 40 mL of water was added, and the phases were separated. The organic layer was washed with saturated sodium bicarbonate solution, dried over Na_2_SO_4_ and concentrated to dryness. ^1^H NMR (400 MHz, Chloroform-*d*) δ 7.70 – 7.55 (m, 6H), 7.54 – 7.45 (m, 2H), 7.45 – 7.35 (m, 6H), 3.88 (s, 2H), 1.83 (s, 2H). ^31^P NMR (162 MHz, CDCl_3_) δ 29.01. ^13^C NMR (101 MHz, CDCl_3_) δ 147.41, 147.38, 133.17, 132.45, 132.35, 132.12, 132.02, 131.96, 131.93, 131.25, 130.20, 128.58, 128.46, 127.28, 127.15, 46.12.

## General procedure for the preparation of 4-(diphenylphosphoryl)benzyl cinnamates (11)

BndppOH **4** (1.85 g, 1.6 mmol, 1 eq), cinnamic acid **10** (1.76 mmol, 1.1 eq), and dry DCM (30 mL) were stirred at 0 °C in a 50 mL Schlenck flask. EDCI(HCl) (0.34 g, 1.76 mmol, 1.1 eq) was added, and the reaction was stirred for 10 min, at which point DMAP (20 mg, 10 mol%) was added, the reaction was brought to room temperature and stirred for 12 h. The reaction mixture was washed twice with satd. NH_4_Cl (aq), followed by two washes with satd. Na_2_CO_3_ (aq). The combined organic layers were dried with Na_2_SO_4_, filtered, and evacuated to afford the crude protected cinnamic acid. GAP purification was performed by dissolving the crude mixture in a minimal amount of ethyl acetate, followed by precipitation with petroleum ether and filtration of the resulting precipitate.

*4-(diphenylphosphoryl)benzyl cinnamate* ***(11a)***

White solid; 89% (624 mg); ^1^H NMR (400 MHz, Chloroform-*d*) δ 7.76 – 7.60 (m, 7H), 7.57 – 7.40 (m, 10H), 7.40 – 7.32 (m, 3H), 6.48 (d, *J* = 16.0 Hz, 1H), 5.29 (s, 2H). ^31^P NMR (162 MHz, CDCl_3_) δ 28.75. ^13^C NMR (101 MHz, CDCl_3_) δ 166.67, 145.73, 140.30, 140.27, 134.25, 133.01, 132.55, 132.45, 132.19, 132.09, 131.97, 130.60, 129.01, 128.68, 128.56, 128.23, 127.97, 127.85, 117.50, 65.57. HRMS (ESI) Calculated for C_28_H_23_O_3_P [M+Na]^+^: 461.1283 Found: 461.1285

*4-(diphenylphosphoryl)benzyl-3-(naphthalen-1-yl)acrylate* ***(11b)***

White solid; 72% (562 mg); ^1^H NMR (400 MHz, Chloroform-*d*) δ 8.58 (dd, *J* = 15.7, 3.0 Hz, 1H), 8.15 (d, *J* = 8.3 Hz, 1H), 7.86 (t, *J* = 9.4 Hz, 2H), 7.78 – 7.60 (m, 7H), 7.60 – 7.37 (m, 11H), 6.57 (dd, *J* = 15.7, 3.0 Hz, 1H), 5.34 (s, 2H). ^31^P NMR (162 MHz, CDCl_3_) δ 28.85. ^13^C NMR (101 MHz, CDCl_3_) δ 166.49, 142.61, 140.25, 140.22, 133.64, 132.95, 132.87, 132.49, 132.39, 132.11, 132.06, 132.01, 131.92, 131.84, 131.44, 131.35, 130.79, 128.77, 128.62, 128.50, 127.96, 127.84, 126.98, 126.29, 125.45, 125.13, 123.24, 119.96, 65.58. HRMS (ESI) Calculated for C_32_H_25_O_3_P [M+Na]^+^: 511.1439 Found: 511.1447

*4-(diphenylphosphoryl)benzyl-2-methylcinnamate* ***(11c)***

White solid; 65% (470 mg); ^1^H NMR (400 MHz, Chloroform-*d*) δ 8.00 (d, *J* = 15.8 Hz, 1H), 7.72 – 7.59 (m, 6H), 7.53 – 7.36 (m, 9H), 7.24 – 7.09 (m, 3H), 6.38 (d, *J* = 15.9 Hz, 1H), 5.26 (s, 2H), 2.36 (s, 3H). ^31^P NMR (162 MHz, CDCl_3_) δ 28.54. ^13^C NMR (101 MHz, CDCl_3_) δ 166.17, 142.86, 139.87, 137.30, 132.68, 132.56, 132.02, 131.92, 131.67, 131.60, 131.57, 131.52, 130.42, 129.84, 128.18, 128.06, 127.46, 127.33, 126.01, 125.96, 117.97, 65.01, 19.34. HRMS (ESI) Calculated for C_29_H_25_O_3_P [M+Na]^+^: 475.1439 Found: 475.1444.

*4-(diphenylphosphoryl)benzyl-4-methylcinnamate* ***(11d)***

White solid; 66% (477 mg); ^1^H NMR (400 MHz, Chloroform-*d*) δ 7.74 – 7.60 (m, 7H), 7.55 – 7.35 (m, 10H), 7.15 (d, *J* = 7.9 Hz, 2H), 6.42 (d, *J* = 16.0 Hz, 1H), 5.26 (s, 2H), 2.32 (s, 3H). ^31^P NMR (162 MHz, CDCl_3_) δ 28.68. ^13^C NMR (101 MHz, CDCl_3_) δ 166.71, 145.61, 140.95, 140.35, 132.94, 132.89, 132.43, 132.32, 132.08, 132.05, 132.00, 131.98, 131.90, 131.85, 131.45, 129.64, 128.58, 128.46, 128.14, 127.85, 127.73, 116.30, 65.36, 21.46. HRMS (ESI) Calculated for C_29_H_25_O_3_P [M+Na]^+^: 475.1439 Found: 475.1444.

*4-(diphenylphosphoryl)benzyl-2-methoxycinnamate* ***(11e)***

White solid; 68% (509 mg); ^1^H NMR (400 MHz, Chloroform-*d*) δ 8.04 (d, *J* = 16.2 Hz, 1H), 7.67 (dt, *J* = 13.5, 7.8 Hz, 6H), 7.58 – 7.41 (m, 9H), 7.34 (t, *J* = 8.0 Hz, 1H), 6.99 – 6.86 (m, 2H), 6.58 (dd, *J* = 16.2, 2.7 Hz, 1H), 5.29 (s, 2H), 3.86 (d, *J* = 2.7 Hz, 3H).  ^31^P NMR (162 MHz, CDCl_3_) δ 28.95. ^13^C NMR (101 MHz, CDCl_3_) δ 167.22, 158.49, 141.22, 140.52, 132.96, 132.80, 132.49, 132.39, 132.18, 132.08, 131.92, 131.81, 129.19, 128.67, 128.55, 127.93, 127.81, 123.19, 120.78, 117.95, 111.22, 65.38, 55.55. HRMS (ESI) Calculated for C_29_H_25_O_4_P [M+Na]^+^: 491.1388 Found: 491.1393

*4-(diphenylphosphoryl)benzyl-4-methoxycinnamate* ***(11f)***

White solid; 77% (577 mg); ^1^H NMR (400 MHz, Chloroform-*d*) δ 7.74 – 7.61 (m, 7H), 7.57 – 7.44 (m, 10H), 6.90 (dd, *J* = 8.8, 2.5 Hz, 2H), 6.35 (dd, *J* = 15.8, 2.6 Hz, 1H), 5.28 (s, 2H), 3.83 (d, *J* = 2.7 Hz, 3H). ^31^P NMR (162 MHz, CDCl3) δ 28.89. ^13^C NMR (101 MHz, CDCl_3_) δ 167.07, 161.67, 145.45, 140.52, 133.02, 132.93, 132.57, 132.47, 132.24, 132.14, 131.99, 131.90, 129.98, 128.72, 128.60, 127.97, 127.85, 127.03, 114.92, 114.48, 65.46, 55.52. HRMS (ESI) Calculated for C_29_H_25_O_4_P [M+Na]^+^: 491.1388 Found: 491.1392

*4-(diphenylphosphoryl)benzyl-3,4-dimethoxycinnamate* ***(11g)***

White solid; 63 % (502 mg); ^1^H NMR (400 MHz, Chloroform-*d*) δ 7.72 – 7.58 (m, 7H), 7.55 – 7.37 (m, 8H), 7.07 (d, *J* = 8.4 Hz, 1H), 7.01 (s, 1H), 6.82 (d, *J* = 7.8 Hz, 1H), 6.33 (dd, *J* = 16.0, 2.7 Hz, 1H), 5.25 (s, 2H), 3.86 (d, *J* = 2.7 Hz, 6H). ^31^P NMR (162 MHz, CDCl_3_) δ 28.80. ^13^C NMR (101 MHz, CDCl_3_) δ 166.82, 151.37, 149.28, 145.58, 140.43, 140.40, 132.94, 132.91, 132.47, 132.37, 132.13, 132.06, 132.03, 131.90, 131.87, 128.63, 128.51, 127.91, 127.79, 127.21, 122.84, 115.09, 111.12, 109.73, 65.39, 56.01, 55.93. HRMS (ESI) Calculated for C_30_H_27_O_5_P [M+Na]^+^: 521.1494 Found: 521.1501.

*4-(diphenylphosphoryl)benzyl-2,3,4-trimethoxycinnamate* ***(11h)***

White solid; 58% (490 mg); ^1^H NMR (400 MHz, Chloroform-*d*) δ 7.95 (d, *J* = 16.1 Hz, 1H), 7.78 – 7.61 (m, 6H), 7.59 – 7.40 (m, 8H), 7.27 (d, *J* = 9.3 Hz, 1H), 6.69 (d, *J* = 8.7 Hz, 1H), 6.48 (d, *J* = 16.2 Hz, 1H), 5.30 (s, 2H), 3.96 – 3.82 (m, 9H). ^31^P NMR (162 MHz, CDCl_3_) δ 28.85. ^13^C NMR (101 MHz, CDCl_3_) δ 167.16, 155.75, 153.39, 142.33, 140.68, 140.55, 132.93, 132.77, 132.44, 132.34, 132.13, 132.05, 132.03, 131.89, 131.73, 128.62, 128.50, 127.86, 127.74, 123.40, 121.23, 116.20, 107.62, 65.29, 61.47, 60.92, 56.09. HRMS (ESI) Calculated for C_31_H_29_O_6_P [M+Na]^+^: 551.1599 Found: 551.1602.

*4-(diphenylphosphoryl)benzyl-4-bromocinnamate* ***(11i)***

White solid; 78% (644 mg); ^1^H NMR (400 MHz, Chloroform-*d*) δ 7.66 (dd, *J* = 12.4, 8.6 Hz, 7H), 7.58 – 7.50 (m, 3H), 7.50 – 7.40 (m, 7H), 7.40 – 7.32 (m, 2H), 6.46 (dd, *J* = 16.2, 2.7 Hz, 1H), 5.28 (s, 2H). ^31^P NMR (162 MHz, CDCl_3_) δ 28.81. ^13^C NMR (101 MHz, CDCl_3_) δ 166.38, 144.29, 140.13, 140.10, 133.13, 132.91, 132.53, 132.43, 132.24, 132.16, 132.06, 131.87, 129.58, 128.67, 128.55, 127.99, 127.86, 124.87, 118.17, 65.67. HRMS (ESI) Calculated for C_28_H_22_BrO_3_P [M+Na]^+^: 539.0388 Found: 539.0385

*4-(diphenylphosphoryl)benzyl-3-fluorocinnamate* ***(11j)***

White solid; 68% (496 mg); ^1^H NMR (400 MHz, Chloroform-*d*) δ 7.72 – 7.57 (m, 7H), 7.54 – 7.36 (m, 8H), 7.33 – 7.20 (m, 2H), 7.16 (dt, *J* = 9.7, 2.0 Hz, 1H), 7.01 (td, *J* = 8.3, 2.5 Hz, 1H), 6.44 (d, *J* = 16.0 Hz, 1H), 5.26 (s, 2H). ^31^P NMR (162 MHz, CDCl_3_) δ 28.64. ^13^C NMR (101 MHz, CDCl_3_) δ 166.09, 164.13, 161.67, 144.10, 144.08, 140.05, 140.02, 136.40, 136.32, 133.00, 132.85, 132.42, 132.31, 132.04, 132.00, 131.97, 131.94, 131.81, 130.50, 130.42, 128.56, 128.44, 127.88, 127.76, 124.12, 124.09, 118.85, 117.40, 117.18, 114.40, 114.18, 65.56. HRMS (ESI) Calculated for C_28_H_22_FO_3_P [M+Na]^+^: 479.1188 Found: 479.1198.

*4-(diphenylphosphoryl)benzyl-4-bromo-2-fluorocinnamate* ***(11k)***

White solid; 81% (692 mg); ^1^H NMR (400 MHz, Chloroform-*d*) δ 8.03 (d, *J* = 16.0 Hz, 1H), 7.74 – 7.61 (m, 6H), 7.61 – 7.38 (m, 9H), 7.33 (dd, *J* = 8.1, 2.6 Hz, 1H), 7.03 (td, *J* = 8.3, 2.6 Hz, 1H), 6.37 (d, *J* = 15.9 Hz, 1H), 5.29 (s, 2H). ^31^P NMR (162 MHz, CDCl_3_) δ 29.04. ^13^C NMR (101 MHz, CDCl_3_) δ 165.72, 164.30, 161.76, 142.53, 140.02, 139.99, 132.99, 132.86, 132.37, 132.27, 132.00, 131.90, 130.54, 130.50, 129.00, 128.91, 128.52, 128.40, 127.80, 127.67, 125.67, 125.57, 120.67, 120.43, 120.02, 120.00, 115.39, 115.18, 65.54. HRMS (ESI) Calculated for C_28_H_21_BrFO_3_P [M+Na]^+^: 557.0293 Found: 557.0298

*4-(diphenylphosphoryl)benzyl-2-chloro-5-nitrocinnamate* ***(11l)***

White solid; 93% (769 mg); ^1^H NMR (400 MHz, Chloroform-*d*) δ 8.47 (d, *J* = 3.1 Hz, 1H), 8.20 – 8.00 (m, 2H), 7.79 – 7.35 (m, 15H), 6.62 (d, *J* = 15.9 Hz, 1H), 5.32 (s, 2H). ^31^P NMR (162 MHz, CDCl_3_) δ 28.81. ^13^C NMR (101 MHz, CDCl_3_) δ 165.39, 146.80, 141.25, 139.09, 134.03, 132.84, 132.58, 132.48, 132.15, 132.06, 131.80, 131.35, 128.68, 128.56, 128.07, 127.95, 125.28, 123.09, 122.61, 66.08. HRMS (ESI) Calculated for C_28_H_21_ClNO_5_P [M+Na]^+^: 540.0744 Found: 540.0745

*4-(diphenylphosphoryl)benzyl-4-(tert-butyl**)cinnamate* ***(11m)***

White solid; 75% (593 mg); ^1^H NMR (400 MHz, Chloroform-*d*) δ 8.11 (dd, *J* = 8.3, 2.5 Hz, 1H), 7.78 – 7.61 (m, 8H), 7.56 – 7.37 (m, 10H), 6.45 (d, *J* = 16.0 Hz, 1H), 5.28 (s, 2H), 1.31 (s, 9H). ^31^P NMR (162 MHz, CDCl_3_) δ 28.82. ^13^C NMR (101 MHz, CDCl_3_) δ 166.85, 154.17, 145.61, 140.37, 132.92, 132.50, 132.40, 132.16, 132.06, 131.88, 131.47, 129.49, 128.65, 128.54, 127.94, 127.82, 125.96, 116.48, 65.46, 34.95, 31.19. HRMS (ESI) Calculated for C_32_H_31_O_3_P [M+Na]^+^: 517.1909 Found: 517.1910.

*4-(diphenylphosphoryl)benzyl-4-chloro-3-nitrocinnamate* ***(11n)***

White solid; 86% (712 mg); ^1^H NMR (400 MHz, Chloroform-*d*) δ 7.99 (s, 1H), 7.74 – 7.58 (m, 8H), 7.58 – 7.37 (m, 9H), 6.54 (d, *J* = 15.9 Hz, 1H), 5.29 (s, 2H). ^31^P NMR (162 MHz, CDCl_3_) δ 29.16. ^13^C NMR (101 MHz, CDCl_3_) δ 165.56, 148.24, 141.48, 139.81, 134.31, 132.64, 132.59, 132.55, 132.45, 132.19, 132.16, 132.13, 132.03, 131.60, 128.69, 128.57, 128.06, 127.94, 124.66, 121.08, 65.97. HRMS (ESI) Calculated for C_28_H_21_ClNO_5_P [M+Na]^+^: 540.0744 Found: 540.0746

*4-(diphenylphosphoryl)benzyl-2-nitrocinnamate* ***(11o)***

White solid; 75% (580 mg); ^1^H NMR (400 MHz, Chloroform-*d*) δ 8.17 (d, *J* = 15.8 Hz, 1H), 8.03 (d, *J* = 8.2 Hz, 1H), 7.76 – 7.59 (m, 8H), 7.57 – 7.38 (m, 9H), 6.40 (d, *J* = 15.6 Hz, 1H), 5.30 (s, 2H). ^31^P NMR (162 MHz, CDCl_3_) δ 28.83. ^13^C NMR (101 MHz, CDCl_3_) δ 165.47, 148.38, 141.06, 140.00, 133.70, 133.16, 132.94, 132.60, 132.50, 132.21, 132.11, 131.90, 130.59, 130.52, 129.26, 128.72, 128.60, 128.03, 127.91, 125.07, 122.62, 65.92. HRMS (ESI) Calculated for C_28_H_22_NO_5_P [M+Na]^+^: 506.1133 Found: 506.1138

*4-(diphenylphosphoryl)benzyl-4-dimethylaminocinnamate* ***(11p)***

Yellowish solid; 81% (624 mg); ^1^H NMR (400 MHz, Chloroform-*d*) δ 7.70 – 7.57 (m, 7H), 7.53 – 7.32 (m, 10H), 6.59 (d, *J* = 8.4 Hz, 2H), 6.23 (d, *J* = 15.8 Hz, 1H), 5.23 (s, 2H), 2.93 (s, 6H). ^31^P NMR (162 MHz, CDCl_3_) δ 28.79. ^13^C NMR (101 MHz, CDCl_3_) δ 167.34, 151.80, 146.10, 140.73, 140.70, 132.86, 132.54, 132.30, 132.20, 132.00, 131.94, 131.90, 131.82, 131.50, 129.80, 128.51, 128.39, 127.71, 127.59, 121.79, 111.68, 111.33, 64.93, 39.97. HRMS (ESI) Calculated for C_30_H_28_NO_3_P [M+Na]^+^: 504.1704 Found: 504.1717

## General procedure for the preparation of 4-(diphenylphosphoryl)benzyl cinnamides (12)

To a stirred solution of 1.6 mmol cinnamic acid **10** and dppBnNH_2_ (0.49 g, 1.6 mmol) in 30 mL anhydrous DCM is added DCC (0.33 g, 1.6 mmol) at room temperature, which is then stirred overnight. Precipitated urea is then filtered off and the filtrate evaporated down in vacuo. GAP purification was performed by dissolving the crude mixture in a minimal amount of ethyl acetate, followed by precipitation with petroleum ether and filtration of the resulting precipitate.

*N-(4-(diphenylphosphoryl)benzyl)cinnamamide* ***(12a)***

White solid; 92% (643mg); ^1^H NMR (400 MHz, Chloroform-*d*) δ 8.09 (t, *J* = 5.3 Hz, 1H), 7.63 (d, *J* = 15.7 Hz, 1H), 7.60 – 7.49 (m, 6H), 7.45 – 7.35 (m, 7H), 7.33 – 7.23 (m, 5H), 6.67 (d, *J* = 15.7 Hz, 1H), 4.57 (d, *J* = 5.9 Hz, 2H). ^31^P NMR (162 MHz, CDCl_3_) δ 29.83. ^13^C NMR (101 MHz, CDCl_3_) δ 166.57, 143.52, 143.49, 140.88, 135.20, 132.78, 132.41, 132.31, 132.25, 132.22, 132.11, 132.02, 131.74, 131.17, 130.12, 129.54, 128.80, 128.78, 128.66, 128.03, 127.90, 121.21, 43.28. HRMS (ESI) Calculated for C_28_H_24_NO_2_P [M+Na]^+^: 460.1442 Found: 460.1445.

*N-(4-(diphenylphosphoryl)benzyl)-4-methylcinnamamide* ***(12b)***

White solid; 94% (679 mg); ^1^H NMR (400 MHz, Chloroform-*d*) δ 8.26 (t, *J* = 6.0 Hz, 1H), 7.65 – 7.48 (m, 7H), 7.46 – 7.35 (m, 6H), 7.33 – 7.23 (m, 4H), 7.06 (d, *J* = 7.8 Hz, 2H), 6.65 (d, *J* = 15.7 Hz, 1H), 4.55 (d, *J* = 5.8 Hz, 2H), 2.32 (s, 3H). ^31^P NMR (162 MHz, CDCl_3_) δ 29.86. ^13^C NMR (101 MHz, CDCl_3_) δ 166.49, 143.34, 143.31, 140.37, 139.35, 132.44, 132.10, 132.00, 131.89, 131.85, 131.75, 131.65, 131.40, 130.67, 129.62, 129.15, 128.42, 128.29, 127.64, 127.51, 119.89, 42.86, 21.15. HRMS (ESI) Calculated for C_29_H_26_NO_2_P [M+Na]^+^: 474.1599 Found: 474.1604.

*N-(4-(diphenylphosphoryl)benzyl)-2-methoxycinnamamide* ***(12c)***

White solid; 96% (718 mg); ^1^H NMR (400 MHz, Chloroform-*d*) δ 8.17 (t, *J* = 6.0 Hz, 1H), 7.92 (d, *J* = 15.8 Hz, 1H), 7.59 – 7.43 (m, 6H), 7.42 – 7.18 (m, 10H), 6.84 – 6.70 (m, 3H), 4.51 (d, *J* = 5.9 Hz, 2H), 3.70 (s, 3H). ^31^P NMR (162 MHz, CDCl_3_) δ 29.69. ^13^C NMR (101 MHz, CDCl_3_) δ 167.09, 158.11, 143.67, 143.64, 136.00, 132.74, 132.21, 132.10, 132.08, 132.06, 131.99, 131.89, 131.70, 130.94, 130.57, 129.89, 128.63, 128.51, 127.84, 127.72, 124.09, 121.80, 120.53, 111.06, 55.35, 43.09. HRMS (ESI) Calculated for C_29_H_26_NO_3_P [M+Na]^+^: 490.1548 Found: 490.1552.

*N-(4-(diphenylphosphoryl)benzyl)-3,4-dimethoxycinnamamide* ***(12d)***

White solid; 96% (764 mg); ^1^H NMR (400 MHz, Chloroform-*d*) δ 8.06 (t, *J* = 6.0 Hz, 1H), 7.62 – 7.46 (m, 7H), 7.43 – 7.32 (m, 6H), 7.31 – 7.24 (m, 2H), 7.02 – 6.88 (m, 2H), 6.76 (d, *J* = 8.2 Hz, 1H), 6.58 (d, *J* = 15.6 Hz, 1H), 4.55 (d, *J* = 5.8 Hz, 2H), 3.86 (s, 3H), 3.69 (s, 3H). ^31^P NMR (162 MHz, CDCl_3_) δ 29.76. ^13^C NMR (101 MHz, CDCl_3_) δ 166.81, 150.46, 149.10, 143.64, 140.61, 132.80, 132.31, 132.21, 132.18, 132.04, 131.94, 131.09, 130.04, 128.73, 128.61, 128.23, 127.90, 127.78, 122.02, 119.22, 111.12, 109.78, 56.01, 55.78, 43.16. HRMS (ESI) Calculated for C_30_H_28_NO_4_P [M+Na]^+^: 520.1654 Found: 520.1661.

*N-(4-(diphenylphosphoryl)benzyl)-2,3,4-trimethoxycinnamamide* ***(12e)***

White solid; 96% (810 mg); ^1^H NMR (400 MHz, Chloroform-*d*) δ 8.10 (t, *J* = 6.0 Hz, 1H), 7.78 (d, *J* = 15.8 Hz, 1H), 7.59 – 7.45 (m, 6H), 7.43 – 7.33 (m, 6H), 7.28 (dd, *J* = 8.4, 2.8 Hz, 2H), 7.07 (d, *J* = 8.8 Hz, 1H), 6.66 (d, *J* = 15.8 Hz, 1H), 6.52 (d, *J* = 8.7 Hz, 1H), 4.51 (d, *J* = 5.9 Hz, 2H), 3.85 – 3.72 (m, 9H). ^31^P NMR (162 MHz, CDCl_3_) δ 29.77. ^13^C NMR (101 MHz, CDCl_3_) δ 167.08, 154.77, 153.04, 143.69, 143.66, 142.34, 135.68, 132.71, 132.22, 132.12, 132.10, 132.07, 132.00, 131.90, 131.67, 130.92, 129.88, 128.63, 128.51, 127.81, 127.68, 122.99, 122.17, 120.32, 107.51, 61.27, 60.85, 56.03, 43.08. HRMS (ESI) Calculated for C_31_H_30_NO_5_P [M+Na]^+^: 550.1759 Found: 550.1766.

*N-(4-(diphenylphosphoryl)benzyl)-4-bromocinnamamide* ***(12f)***

White solid; 91% (750 mg); ^1^H NMR (400 MHz, Chloroform-*d*) δ 8.47 (t, *J* = 5.9 Hz, 1H), 7.59 – 7.48 (m, 7H), 7.45 – 7.31 (m, 8H), 7.31 – 7.16 (m, 4H), 6.70 (d, *J* = 15.5 Hz, 1H), 4.56 (d, *J* = 5.8 Hz, 2H). ^31^P NMR (162 MHz, CDCl_3_) δ 30.19. ^13^C NMR (101 MHz, CDCl_3_) δ 166.34, 143.53, 139.31, 134.19, 132.61, 132.34, 132.24, 132.05, 131.95, 131.56, 130.91, 129.86, 129.27, 128.81, 128.69, 128.06, 127.93, 123.49, 122.07, 43.24. HRMS (ESI) Calculated for C_28_H_23_BrNO_2_P [M+Na]^+^: 538.0547 Found: 538.0541.

*N-(4-(diphenylphosphoryl)benzyl)-3-fluorocinnamamide* ***(12g)***

White solid; 89% (648 mg); ^1^H NMR (400 MHz, Chloroform-*d*) δ 8.55 (t, *J* = 6.0 Hz, 1H), 7.64 – 7.47 (m, 7H), 7.46 – 7.17 (m, 9H), 7.12 (d, *J* = 7.7 Hz, 1H), 7.06 (dt, *J* = 9.9, 2.0 Hz, 1H), 6.97 (td, *J* = 8.3, 2.6 Hz, 1H), 6.72 (d, *J* = 15.7 Hz, 1H), 4.57 (d, *J* = 5.8 Hz, 2H). ^31^P NMR (162 MHz, CDCl_3_) δ 30.06. ^13^C NMR (101 MHz, CDCl_3_) δ 166.24, 164.23, 161.78, 143.52, 139.27, 137.64, 137.56, 132.63, 132.34, 132.31, 132.24, 132.03, 131.93, 131.58, 130.32, 130.24, 128.82, 128.70, 128.10, 127.98, 124.07, 122.81, 116.39, 116.17, 113.96, 113.74, 43.26. HRMS (ESI) Calculated for C_28_H_23_FNO_2_P [M+Na]^+^: 478.1348 Found: 478.1348.

*N-(4-(diphenylphosphoryl)benzyl)-4-bromo-2-fluorocinnamamide* ***(12h)***

White solid; 88% (751 mg); ^1^H NMR (400 MHz, Chloroform-*d*) δ 8.63 (t, *J* = 5.9 Hz, 1H), 7.88 (d, *J* = 15.6 Hz, 1H), 7.59 – 7.45 (m, 6H), 7.42 – 7.22 (m, 10H), 6.82 (td, *J* = 8.3, 2.6 Hz, 1H), 6.64 (d, *J* = 15.6 Hz, 1H), 4.55 (d, *J* = 5.9 Hz, 2H). ^31^P NMR (162 MHz, CDCl_3_) δ 30.12. ^13^C NMR (101 MHz, CDCl_3_) δ 165.95, 163.81, 161.29, 143.56, 137.83, 132.61, 132.29, 132.20, 132.03, 131.93, 131.79, 131.76, 131.57, 130.89, 129.84, 128.77, 128.65, 128.08, 127.96, 125.39, 125.30, 124.29, 124.27, 120.58, 120.34, 115.07, 114.85, 43.24. HRMS (ESI) Calculated for C_28_H_22_BrFNO_2_P [M+H]^+^: 534.0634 Found: 534.0637.

*N-(4-(diphenylphosphoryl)benzyl)-4-(tert-butyl)cinnamamide* ***(12i)***

White solid; 91% (718 mg); ^1^H NMR (400 MHz, Chloroform-*d*) δ 8.09 (t, *J* = 6.0 Hz, 1H), 7.66 – 7.48 (m, 7H), 7.45 – 7.36 (m, 6H), 7.36 – 7.24 (m, 6H), 6.65 (d, *J* = 15.7 Hz, 1H), 4.55 (d, *J* = 5.8 Hz, 2H), 1.30 (s, 9H). ^31^P NMR (162 MHz, CDCl_3_) δ 29.86. ^13^C NMR (101 MHz, CDCl_3_) δ 166.48, 147.37, 144.49, 139.46, 132.59, 132.33, 132.25, 132.04, 131.94, 131.53, 128.81, 128.69, 128.38, 127.97, 127.84, 124.66, 120.27, 109.25, 56.29, 43.20. HRMS (ESI) Calculated for C_32_H_32_NO_2_P [M+Na]^+^: 516.2068 Found: 516.2072.

*N-(4-(diphenylphosphoryl)benzyl)-4-chloro-3-nitrocinnamamide* ***(12j)***

White solid; 96% (793 mg); ^1^H NMR (400 MHz, Chloroform-*d*) δ 8.91 (t, *J* = 5.8 Hz, 1H), 7.81 (s, 1H), 7.62 – 7.22 (m, 17H), 6.85 (d, *J* = 15.7 Hz, 1H), 4.57 (d, *J* = 5.7 Hz, 2H). ^31^P NMR (162 MHz, CDCl_3_) δ 30.18. ^13^C NMR (101 MHz, CDCl_3_) δ 165.52, 148.25, 143.38, 136.53, 135.61, 132.44, 132.33, 132.23, 132.06, 132.00, 131.90, 131.39, 130.92, 129.87, 128.86, 128.74, 128.13, 128.01, 127.81, 127.13, 125.15, 123.70, 43.27. HRMS (ESI) Calculated for C_28_H_22_ClN_2_O_4_P [M+Na]^+^: 539.0903 Found: 539.0906.

*N-(4-(diphenylphosphoryl)benzyl)-2-nitrocinnamamide* ***(12k)***

White solid; 92% (710 mg); ^1^H NMR (400 MHz, Chloroform-*d*) δ 8.59 (t, *J* = 5.9 Hz, 1H), 8.00 (d, *J* = 15.6 Hz, 1H), 7.92 (dd, *J* = 6.1, 2.3 Hz, 1H), 7.55 – 7.46 (m, 6H), 7.46 – 7.40 (m, 3H), 7.40 – 7.27 (m, 8H), 6.68 (d, *J* = 15.6 Hz, 1H), 4.54 (d, *J* = 5.8 Hz, 2H). ^31^P NMR (162 MHz, CDCl_3_) δ 29.92. ^13^C NMR (101 MHz, CDCl_3_) δ 165.51, 148.44, 143.44, 143.41, 135.62, 133.19, 132.55, 132.28, 132.18, 131.98, 131.88, 131.51, 131.38, 130.89, 129.85, 129.52, 129.07, 128.74, 128.62, 128.05, 127.92, 126.70, 124.73, 43.21. HRMS (ESI) Calculated for C_28_H_23_N_2_O_4_P [M+Na]^+^: 505.1292 Found: 505.1291.

*N-(4-(diphenylphosphoryl)benzyl)-4-dimethylaminocinnamamide* ***(12l)***

White solid; 92% (707 mg); ^1^H NMR (400 MHz, Chloroform-*d*) δ 7.66 – 7.35 (m, 14H), 7.34 – 7.27 (m, 4H), 6.57 (d, *J* = 8.9 Hz, 2H), 6.40 (d, *J* = 15.6 Hz, 1H), 4.54 (d, *J* = 5.9 Hz, 2H), 2.95 (s, 6H). ^31^P NMR (162 MHz, CDCl_3_) δ 29.60. ^13^C NMR (101 MHz, CDCl_3_) δ 167.37, 151.34, 143.76, 143.73, 141.38, 132.91, 132.36, 132.26, 132.11, 132.08, 132.01, 131.87, 131.14, 130.10, 129.38, 128.69, 128.57, 127.84, 127.71, 122.97, 115.74, 111.94, 43.16, 40.26. HRMS (ESI) Calculated for C_30_H_29_N_2_O_2_P [M+Na]^+^: 503.1864 Found: 503.1866.

# Aminochlorination of 4-(diphenylphosphoryl)benzyl cinnamates (13)

**Typical procedure:** Into a dry vial was added 4-(diphenylphosphoryl)benzyl cinnamate **11** (1.5 mmol, 1 eq), 4-TsNCl_2_, (3 mmol, 2 eq) 4-TsNH_2_ (3 mmol, 2 eq), PhI(OAc)_2_ (20 mol%) and freshly activated 4 Å molecular sieves (150 mg). The resulting mixture was capped under argon protection. Anhydrous DCM (3 mL) was injected through a syringe and the reaction was allowed to stir at reflux for 48 h. The progress of the reaction was monitored through TLC analysis. After completion, the reaction was quenched by dropwise addition of saturated aqueous Na_2_SO_3_ solution (2 mL). The phases were separated, and the aqueous phase was extracted with DCM (3×10 mL). The combined organic layers were washed with water and brine, dried over anhydrous calcium sulfate, and concentrated to dryness. GAP purification was performed by dissolving the crude mixture in a minimal amount of ethyl acetate or DCM, followed by precipitation with petroleum ether and filtration of the resulting precipitate.

*4-(diphenylphosphoryl)benzyl 3-chloro-2-((4-methylphenyl)sulfonamido)-3-phenylpropanoate* ***(13a)***

White solid, 83% (80 mg); ^1^H NMR (400 MHz, Chloroform-*d*) δ 7.70 – 7.51 (m, 10H), 7.50 – 7.41 (m, 4H), 7.33 – 7.11 (m, 9H), 5.89 (d, *J* = 9.8 Hz, 1H), 5.13 (d, *J* = 6.4 Hz, 1H), 4.94 (q, *J* = 12.8 Hz, 2H), 4.49 (dd, *J* = 9.8, 6.4 Hz, 1H), 2.33 (d, *J* = 6.6 Hz, 3H). ^31^P NMR (162 MHz, CDCl_3_) δ 28.75. ^13^C NMR (101 MHz, CDCl_3_) δ 168.41, 143.82, 138.53, 138.50, 136.69, 135.85, 132.74, 132.47, 132.37, 132.24, 132.21, 132.17, 132.07, 131.70, 129.66, 129.58, 129.10, 128.75, 128.69, 128.63, 128.53, 128.19, 128.07, 127.67, 127.27, 127.05, 66.91, 62.08, 61.72, 21.61. HRMS (ESI) Calculated for C_35_H_31_ClNO_5_PS [M+Na]^+^: 666.1247 Found: 666.1244.

*4-(diphenylphosphoryl)benzyl-3-chloro-2-((4-methylphenyl)sulfonamido)-3-(naphthalen-1-yl)propanoate* ***(13b)***

White solid, 77% (80 mg); ^1^H NMR (400 MHz, Chloroform-*d*) δ 7.99 – 7.92 (m, 1H), 7.85 – 7.76 (m, 1H), 7.72 – 7.41 (m, 19H), 7.22 (d, *J* = 7.8 Hz, 1H), 7.08 (t, *J* = 7.6 Hz, 3H), 6.22 (d, *J* = 9.7 Hz, 1H), 5.98 (d, *J* = 6.3 Hz, 1H), 4.94 – 4.80 (m, 2H), 4.76 (dd, *J* = 9.7, 6.3 Hz, 1H), 2.30 (s, 3H). ^31^P NMR (162 MHz, CDCl_3_) δ 28.59. ^13^C NMR (101 MHz, CDCl_3_) δ 168.28, 143.77, 138.41, 136.43, 133.66, 132.76, 132.35, 132.25, 132.17, 132.14, 132.04, 131.72, 131.22, 130.14, 129.77, 129.60, 129.26, 129.18, 128.71, 128.59, 128.00, 127.87, 127.12, 126.74, 126.60, 126.02, 125.11, 122.16, 66.79, 60.63, 21.59. HRMS (ESI) Calculated for C_39_H_33_ClNO_5_PS [M+Na]^+^: 716.1403 Found: 716.1405.

*4-(diphenylphosphoryl)benzyl-3-chloro-2-((4-methylphenyl)sulfonamido)-3-(o-tolyl)propanoate* ***(13c)***

White solid, 82% (81 mg); ^1^H NMR (400 MHz, Chloroform-*d*) δ 7.69 – 7.50 (m, 10H), 7.48 – 7.40 (m, 5H), 7.31 – 7.19 (m, 2H), 7.16 – 6.97 (m, 5H), 6.37 (d, *J* = 10.5 Hz, 1H), 5.34 (d, *J* = 7.6 Hz, 1H), 5.02 – 4.83 (m, 2H), 4.52 (dd, *J* = 9.9, 7.6 Hz, 1H), 2.32 (s, 3H), 2.25 (s, 3H). ^31^P NMR (162 MHz, CDCl_3_) δ 28.63. ^13^C NMR (101 MHz, CDCl_3_) δ 168.78, 143.80, 138.57, 136.62, 135.82, 134.25, 133.29, 132.79, 132.40, 132.30, 132.17, 132.15, 132.05, 131.75, 130.76, 129.63, 128.88, 128.70, 128.57, 128.02, 127.90, 127.74, 127.25, 126.51, 66.76, 60.51, 58.48, 21.58, 19.23. HRMS (ESI) Calculated for C_36_H_33_ClNO_5_PS [M+Na]^+^: 680.1403 Found: 680.1407.

*4-(diphenylphosphoryl)benzyl-3-chloro-2-((4-methylphenyl)sulfonamido)-3-(p-tolyl)propanoate* ***(13d)***

White solid, 92% (91 mg); ^1^H NMR (400 MHz, Chloroform-*d*) δ 7.70 – 7.51 (m, 10H), 7.45 (m, 4H), 7.30 – 7.21 (m, 2H), 7.19 – 7.11 (m, 2H), 7.06 (d, *J* = 7.9 Hz, 2H), 6.97 (d, *J* = 7.9 Hz, 2H), 5.87 (d, *J* = 9.8 Hz, 1H), 5.09 (d, *J* = 6.6 Hz, 1H), 5.05 – 4.86 (m, 2H), 4.55 – 4.38 (m, 1H), 2.34 (s, 3H), 2.24 (s, 3H). ^31^P NMR (162 MHz, CDCl_3_) δ 28.62. ^13^C NMR (101 MHz, CDCl_3_) δ 168.53, 143.70, 139.07, 138.59, 138.56, 136.75, 133.46, 132.88, 132.81, 132.43, 132.33, 132.19, 132.16, 132.06, 131.77, 129.64, 129.57, 129.47, 129.33, 129.14, 128.72, 128.60, 128.13, 128.01, 127.56, 127.26, 127.07, 66.84, 62.10, 61.53, 21.61, 21.20. HRMS (ESI) Calculated for C_36_H_33_ClNO_5_PS [M+Na]^+^: 680.1403 Found: 680.1408.

*4-(diphenylphosphoryl)benzyl-3-chloro-3-(2-methoxyphenyl)-2-((4-methylphenyl)sulfonamido)propanoate* ***(13e)***

White solid, 94% (95 mg) ; ^1^H NMR (400 MHz, Chloroform-*d*) δ 7.73 – 7.50 (m, 10H), 7.49 – 7.43 (m, 4H), 7.42 – 7.27 (m, 2H), 7.24 – 7.14 (m, 4H), 6.87 – 6.74 (m, 2H), 5.59 (d, *J* = 9.9 Hz, 1H), 5.53 (d, *J* = 5.9 Hz, 1H), 4.91 (s, 2H), 4.61 (dd, *J* = 9.9, 5.9 Hz, 1H), 3.76 (s, 3H), 2.34 (s, 3H). ^31^P NMR (162 MHz, CDCl_3_) δ 28.68. ^13^C NMR (101 MHz, CDCl_3_) δ 168.52, 156.05, 143.77, 136.90, 132.41, 132.31, 132.20, 132.18, 132.08, 130.18, 129.65, 129.42, 129.05, 128.73, 128.61, 127.93, 127.81, 127.27, 126.96, 123.97, 120.91, 110.53, 66.65, 60.31, 57.30, 55.62, 21.61. HRMS (ESI) Calculated for C_36_H_33_ClNO_6_PS [M+Na]^+^: 696.1352 Found: 696.1355.

*4-(diphenylphosphoryl)benzyl-3-chloro-3-(4-methoxyphenyl)-2-((4-methylphenyl)sulfonamido)propanoate* ***(13f)***

White solid, 87% (87 mg); ^1^H NMR (400 MHz, Chloroform-*d*) δ 7.70 – 7.40 (m, 15H), 7.29 – 7.27 (m, 1H), 7.23 – 7.17 (m, 1H), 7.15 – 7.05 (m, 3H), 6.72 – 6.64 (m, 2H), 6.02 (d, *J* = 9.8 Hz, 1H), 5.10 – 4.88 (m, 3H), 4.48 – 4.37 (m, 1H), 3.70 (s, 3H), 2.33 (s, 3H). ^31^P NMR (162 MHz, CDCl_3_) δ 28.64. ^13^C NMR (101 MHz, CDCl_3_) δ 168.71, 160.10, 143.66, 138.75, 138.73, 136.84, 133.43, 132.80, 132.38, 132.34, 132.15, 132.05, 131.76, 129.55, 129.50, 129.02, 128.94, 128.71, 128.59, 128.12, 128.00, 127.87, 127.23, 127.07, 114.02, 66.82, 62.71, 62.18, 55.30, 21.55. HRMS (ESI) Calculated for C_36_H_33_ClNO_6_PS [M+Na]^+^: 696.1352 Found: 696.1353.

*4-(diphenylphosphoryl)benzyl-3-chloro-3-(3,4-dimethoxyphenyl)-2-((4-methylphenyl)sulfonamido)propanoate* ***(13g)***

Light yellow solid, 79% (83 mg); ^1^H NMR (400 MHz, Chloroform-*d*) δ 7.71 – 7.59 (m, 7H), 7.58 – 7.50 (m, 3H), 7.50 – 7.34 (m, 7H), 7.30 (dd, *J* = 8.2, 2.5 Hz, 1H), 7.12 (d, *J* = 7.9 Hz, 1H), 7.06 (d, *J* = 8.0 Hz, 1H), 6.69 (d, *J* = 5.1 Hz, 1H), 5.84 (d, *J* = 2.8 Hz, 1H), 5.61 (d, *J* = 10.1 Hz, 1H), 5.20 (s, 2H), 5.06 – 4.93 (m, 1H), 3.83 (s, 3H), 3.76 (s, 3H), 2.32 (s, 3H). ^31^P NMR (162 MHz, CDCl_3_) δ 28.67. ^13^C NMR (101 MHz, CDCl_3_) δ 168.27, 149.82, 147.78, 143.64, 138.86, 136.85, 132.58, 132.48, 132.40, 132.18, 132.08, 129.55, 129.44, 128.73, 128.61, 127.84, 127.71, 127.22, 126.61, 125.34, 122.77, 112.69, 111.49, 67.31, 60.53, 59.68, 56.14, 56.03, 21.54. HRMS (ESI) Calculated for C_37_H_35_ClNO_7_PS [M+H]^+^: 704.1639 Found: 704.1635.

*4-(diphenylphosphoryl)benzyl-3-chloro-2-((4-methylphenyl)sulfonamido)-3-(2,3,4-trimethoxyphenyl)propanoate* ***(13h)***

White solid, 84% (92 mg); ^1^H NMR (400 MHz, Chloroform-*d*) 7.73 – 7.36 (m, 17H), 7.24 (d, *J* = 2.4 Hz, 1H), 7.12 – 7.06 (m, 2H), 5.84 (d, *J* = 9.8 Hz, 1H), 5.33 (d, *J* = 7.0 Hz, 1H), 5.18 (s, 2H), 4.55 (dd, *J* = 10.2, 3.1 Hz, 1H), 3.87 (s, 3H), 3.82 (s, 3H), 3.79 (s, 3H), 2.33 (s, 3H). ^31^P NMR (162 MHz, CDCl_3_) δ 28.65, 28.58. ^13^C NMR (101 MHz, CDCl_3_) δ 168.54, 150.93, 149.14, 146.49, 143.81, 138.98, 138.96, 136.88, 132.94, 132.64, 132.46, 132.24, 132.15, 131.90, 129.59, 128.77, 128.76, 128.65, 128.64, 127.87, 127.75, 126.87, 125.36, 125.34, 124.67, 122.73, 67.31, 61.46, 61.26, 61.05, 60.92, 57.79, 21.63. HRMS (ESI) Calculated for C_38_H_37_ClNO_8_PS [M+Na]^+^: 756.1564 Found: 756.1557.

*4-(diphenylphosphoryl)benzyl-3-(4-bromophenyl)-3-chloro-2-((4-methylphenyl)sulfonamido)propanoate* ***(13i)***

White solid, 67% (72 mg); ^1^H NMR (400 MHz, Chloroform-*d*) δ 7.70 – 7.58 (m, 6H), 7.58 – 7.40 (m, 8H), 7.32 – 7.19 (m, 4H), 7.12 (d, *J* = 8.1 Hz, 2H), 7.04 (d, *J* = 8.5 Hz, 2H), 6.38 (d, *J* = 9.8 Hz, 1H), 5.16 – 4.93 (m, 3H), 4.41 (dd, *J* = 9.8, 7.5 Hz, 1H), 2.36 (s, 3H). ^31^P NMR (162 MHz, CDCl_3_) δ 28.69. ^13^C NMR (101 MHz, CDCl_3_) δ 168.56, 143.83, 138.62, 138.59, 136.77, 135.28, 132.75, 132.51, 132.45, 132.41, 132.24, 132.21, 132.19, 132.09, 131.69, 129.62, 129.60, 129.47, 128.77, 128.65, 128.16, 128.03, 127.12, 123.23, 66.97, 62.04, 60.66, 21.67. HRMS (ESI) Calculated for C_35_H_30_BrClNO_5_PS [M+Na]^+^: 744.0352 Found: 744.0349.

*4-(diphenylphosphoryl)benzyl-3-chloro-3-(3-fluorophenyl)-2-((4-methylphenyl)sulfonamido)propanoate* ***(13j)***

White solid, 51% (51 mg); ^1^H NMR (400 MHz, Chloroform-*d*) δ 7.70 – 7.51 (m, 10H), 7.50 – 7.41 (m, 5H), 7.30 – 7.22 (m, 1H), 7.19 – 7.08 (m, 3H), 6.97 (d, *J* = 7.8 Hz, 1H), 6.93 – 6.86 (m, 2H), 6.09 (d, *J* = 9.7 Hz, 1H), 5.10 (d, *J* = 6.4 Hz, 1H), 4.98 (q, *J* = 13.7 Hz, 2H), 4.44 (dd, *J* = 9.8, 6.7 Hz, 1H), 2.35 (s, 3H). ^31^P NMR (162 MHz, CDCl_3_) δ 28.74. ^13^C NMR (101 MHz, CDCl_3_) δ 168.29, 163.79, 161.33, 143.97, 138.55, 138.47, 138.42, 132.80, 132.55, 132.45, 132.25, 132.21, 132.11, 131.76, 130.30, 130.22, 129.72, 128.77, 128.65, 128.24, 128.11, 127.23, 126.96, 123.49, 123.46, 116.19, 115.98, 115.09, 114.86, 67.08, 62.08, 60.93, 21.63. HRMS (ESI) Calculated for C_35_H_30_ClFNO_5_PS [M+Na]^+^: 684.1153 Found: 684.1155.

*4-(diphenylphosphoryl)benzyl-3-(4-bromo-2-fluorophenyl)-3-chloro-2-((4-methylphenyl)sulfonamido)propanoate* ***(13k)***

White solid, 54% (60 mg); ^1^H NMR (400 MHz, Chloroform-*d*) δ 7.70 – 7.51 (m, 10H), 7.50 – 7.42 (m, 4H), 7.37 (dd, *J* = 8.9, 5.8 Hz, 1H), 7.30 – 7.22 (m, 3H), 7.19 – 7.12 (m, 3H), 6.84 (ddd, *J* = 8.7, 7.7, 2.6 Hz, 1H), 5.87 – 5.79 (m, 1H), 5.49 (d, *J* = 7.3 Hz, 1H), 5.02 – 4.87 (m, 2H), 4.52 (dd, *J* = 10.4, 7.3 Hz, 1H), 2.34 (s, 3H). ^31^P NMR (162 MHz, CDCl_3_) δ 28.63. ^13^C NMR (101 MHz, CDCl_3_) δ 168.33, 163.47, 160.94, 144.08, 138.37, 136.63, 132.53, 132.42, 132.24, 132.22, 132.20, 132.10, 131.54, 131.50, 131.22, 131.13, 129.72, 128.76, 128.64, 128.12, 127.99, 127.30, 123.72, 123.62, 119.89, 115.41, 115.20, 67.06, 60.70, 59.58, 21.62. HRMS (ESI) Calculated for C_35_H_29_BrClFNO_5_PS [M+Na]^+^: 762.0258 Found: 762.0261.

*4-(diphenylphosphoryl)benzyl-3-chloro-3-(2-chloro-4-nitrophenyl)-2-((4-methylphenyl)sulfonamido)propanoate* ***(13l)***

White solid, 47% (51 mg); ^1^H NMR (400 MHz, Chloroform-*d*) δ 8.29 (d, *J* = 2.9 Hz, 1H), 8.01 (dd, *J* = 8.7, 2.7 Hz, 1H), 7.76 – 7.43 (m, 16H), 7.25 (s, 1H), 7.18 (d, *J* = 7.9 Hz, 2H), 5.90 (d, *J* = 10.1 Hz, 1H), 5.58 (d, *J* = 6.6 Hz, 1H), 5.04 (s, 2H), 4.56 (dd, *J* = 9.8, 2.9 Hz, 1H), 2.36 (s, 3H). ^31^P NMR (162 MHz, CDCl_3_) δ 28.74. ^13^C NMR (101 MHz, CDCl_3_) δ 168.03, 146.72, 144.45, 139.68, 138.12, 136.37, 136.15, 132.63, 132.53, 132.26, 132.16, 130.70, 129.88, 128.81, 128.69, 128.06, 127.93, 127.34, 125.28, 124.72, 67.47, 60.49, 57.60, 21.62. HRMS (ESI) Calculated for C_35_H_29_Cl_2_N_2_O_7_PS [M+Na]^+^: 745.0708 Found: 745.0703.

*4-(diphenylphosphoryl)benzyl-3-(4-(tert-butyl)phenyl)-3-chloro-2-((4-methylphenyl)sulfonamido)propanoate* ***(13m)***

White solid, 45% (47 mg); ^1^H NMR (400 MHz, Chloroform-*d*) δ 7.77 – 7.66 (m, 8H), 7.66 – 7.59 (m, 2H), 7.58 – 7.50 (m, 4H), 7.35 – 7.28 (m, 5H), 7.25 – 7.17 (m, 3H), 5.71 (d, *J* = 9.8 Hz, 1H), 5.22 (d, *J* = 6.1 Hz, 1H), 5.08 – 4.96 (m, 2H), 4.57 (dd, *J* = 9.8, 6.0 Hz, 1H), 2.43 (s, 3H), 1.32 (s, 9H). ^31^P NMR (162 MHz, CDCl_3_) δ 28.74. ^13^C NMR (101 MHz, CDCl_3_) δ 168.45, 152.32, 143.89, 138.52, 136.63, 132.71, 132.50, 132.40, 132.23, 132.20, 132.18, 132.08, 129.67, 128.76, 128.64, 128.15, 128.03, 127.36, 125.70, 66.96, 62.05, 61.67, 34.68, 31.31, 21.66. HRMS (ESI) Calculated for C_39_H_39_ClNO_5_PS [M+Na]^+^: 722.1873 Found: 72.1877.

# Aminochlorination of *N*-(4-(diphenylphosphoryl)benzyl)cinnamamides (14)

**Table 1s. Optimization of the reaction conditions with GAP amide substrates ^[a]^**

| Entry | Cat. 20 mol % | Solvent | Yield %^[b]^ | dr^[c]^ |
| --- | --- | --- | --- | --- |
|  | -- | DCM | 13 | ‒ |
|  | Mn(OAc)_2_ | DCM | 20 | ‒ |
|  | FeCl_3_ | DCM | 37 | 4:1 |
|  | PhI(OAc)_2_ | DCM | 73 | 10:1 |
|  | Pd(OAc)_2_ | DCM | 68 | 8:1 |
|  | CuI | DCM | 68 | 8:1 |
|  | Cu(OTf)_2_ | DCM | 60 | 9:1 |
|  | ZnCl_2_ | DCM | 31 | 7:1 |
|  | PhI(OAc)_2_ | CHCl_3_ | 70 | 5:1 |
|  | PhI(OAc)_2_ | MeCN | 61 | 9:1 |
|  | PhI(OAc)_2_ | THF | traces | ‒ |
|  | PhI(OAc)_2_ | DCE | 38 | 4:1 |
|  | PhI(OAc)_2_ | Toluene | 35 | 5:1 |
|  | PhI(OAc)_2_ | MeOH | ‒ | ‒ |
|  | PhI(OAc)_2_ | Acetone | 17 | 3:1 |
|  | PhI(OAc)_2_ | Dioxane | 23 | ‒ |
| [a] Unless otherwise indicated, reactions were performed with 0.15 mmol of **14**, 4-TsNH_2_ (3 mmol), 4-TsNCl_2_ (3 mmol), 750 mg of 4Å MS in 1.5 mL of solvent at reflux temperature under argon. [b] isolated yields with GAP washing (GAP purification was not conducted for a yield below 38%). [c] The dr values were determined by the analysis of ^1^H and ^31^P NMR spectra. | | | | |

**Typical procedure:** Into a dry vial was added *N*-(4-(diphenylphosphoryl)benzyl)cinnamamide **12** (1.5 mmol, 1 eq), 4-TsNCl_2_, (3 mmol, 2 eq) 4-TsNH_2_ (3 mmol, 2 eq), PhI(OAc)_2_ (20 mol%) and freshly activated 4 Å molecular sieves (150 mg). The resulting mixture was capped under argon protection. Anhydrous DCM (3 mL) was injected through a syringe and the reaction was allowed to stir at reflux for 48 h. The progress of the reaction was monitored through TLC analysis. After completion, the reaction was quenched by dropwise addition of saturated aqueous Na_2_SO_3_ solution (2 mL). The phases were separated, and the aqueous phase was extracted with DCM (3×10 mL). The combined organic layers were washed with water and brine, dried over anhydrous calcium sulfate, and concentrated to dryness. GAP purification was performed by dissolving the crude mixture in a minimal amount of ethyl acetate or DCM, followed by precipitation with petroleum ether and filtration of the resulting precipitate.

*3-chloro-N-(4-(diphenylphosphoryl)benzyl)-2-((4-methylphenyl)sulfonamido)-3-phenylpropanamide* ***(14a)***

White solid, 73% (70 mg); ^1^H NMR (400 MHz, DMSO-*d*_6_) δ 8.87 (t, *J* = 5.8 Hz, 1H), 8.30 (d, *J* = 9.8 Hz, 1H), 7.69 – 7.47 (m, 13H), 7.41 (d, *J* = 8.0 Hz, 2H), 7.37 – 7.20 (m, 7H), 7.15 (d, *J* = 7.9 Hz, 2H), 4.99 (d, *J* = 10.1 Hz, 1H), 4.44 (t, *J* = 10.0 Hz, 1H), 4.15 (ddd, *J* = 51.0, 15.9, 5.7 Hz, 2H), 2.27 (s, 3H). ^31^P NMR (162 MHz, DMSO) δ 25.34. ^13^C NMR (101 MHz, DMSO) δ 168.38, 142.75, 142.73, 142.21, 138.06, 137.65, 133.30, 132.28, 131.98, 131.95, 131.57, 131.45, 131.43, 131.35, 131.33, 130.54, 129.02, 128.75, 128.63, 128.42, 128.17, 127.41, 127.29, 126.25, 60.91, 60.74, 42.03, 20.85. HRMS (ESI) Calculated for C_35_H_32_ClN_2_O_4_PS [M+Na]^+^: 665.1407 Found: 665.1408.

*3-chloro-N-(4-(diphenylphosphoryl)benzyl)-2-((4-methylphenyl)sulfonamido)-3-(p-tolyl)propenamide* ***(14b)***

White solid, 92% (91 mg); ^1^H NMR (400 MHz, Chloroform-*d*) δ 8.66 (s, 1H), 7.60 – 7.38 (m, 12H), 7.29 – 7.05 (m, 5H), 7.02 (d, *J* = 8.0 Hz, 2H), 6.84 (d, *J* = 7.8 Hz, 2H), 6.65 – 6.56 (m, 1H), 5.17 (d, *J* = 7.5 Hz, 1H), 4.66 (ddd, *J* = 9.3, 7.5, 1.6 Hz, 1H), 4.54 – 4.37 (m, 1H), 4.19 (ddd, *J* = 56.9, 15.5, 5.6 Hz, 2H), 2.31 (s, 3H), 2.20 (s, 3H). ^31^P NMR (162 MHz, CDCl_3_) δ 30.25. ^13^C NMR (101 MHz, CDCl_3_) δ 168.96, 143.13, 142.39, 142.36, 138.57, 137.23, 133.99, 132.37, 132.29, 132.17, 132.06, 129.34, 129.13, 128.85, 128.83, 128.73, 128.71, 128.05, 128.00, 127.88, 127.48, 62.97, 61.65, 43.35, 21.60, 21.26. HRMS (ESI) Calculated for C_36_H_34_ClN_2_O_4_PS [M+Na]^+^: 679.1563 Found: 679.1561.

*3-chloro-N-(4-(diphenylphosphoryl)benzyl)-3-(2-methoxyphenyl)-2-((4-methylphenyl)sulfonamido)propenamide* ***(14c)***

White solid, 94% (95 mg); ^1^H NMR (400 MHz, Chloroform-*d*) δ 7.81 (t, *J* = 6.0 Hz, 1H), 7.60 – 7.34 (m, 14H), 7.23 – 7.00 (m, 5H), 6.75 – 6.66 (m, 2H), 6.62 (d, *J* = 8.2 Hz, 1H), 5.08 (t, *J* = 6.2 Hz, 1H), 4.97 (d, *J* = 6.6 Hz, 1H), 4.39 – 4.14 (m, 3H), 3.62 (s, 3H), 2.30 (s, 3H). ^31^P NMR (162 MHz, CDCl_3_) δ 30.15. ^13^C NMR (101 MHz, CDCl_3_) δ 170.67, 155.94, 143.13, 142.55, 136.83, 132.54, 132.31, 132.23, 132.21, 132.11, 132.01, 131.50, 129.46, 128.90, 128.74, 128.61, 127.79, 127.49, 127.45, 127.33, 127.15, 120.94, 110.34, 70.05, 60.37, 55.38, 42.83, 21.52. HRMS (ESI) Calculated for C_36_H_34_ClN_2_O_5_PS [M+Na]^+^: 695.1512 Found: 695.1513.

*3-chloro-3-(3,4-dimethoxyphenyl)-N-(4-(diphenylphosphoryl)benzyl)-2-((4-methylphenyl)sulfonamido)propenamide* ***(14d)***

White solid, 83% (182 mg); ^1^H NMR (400 MHz, Chloroform-*d*) δ 7.87 (d, *J* = 8.4 Hz, 2H), 7.63 – 7.23 (m, 17H), 6.75 – 6.68 (m, 2H), 6.57 (s, 1H), 4.34 (dd, *J* = 15.7, 6.8 Hz, 1H), 4.07 (dd, *J* = 15.8, 5.4 Hz, 1H), 4.01 (d, *J* = 7.5 Hz, 1H), 3.80 – 3.74 (m, 2H), 3.62 (s, 3H), 3.46 (s, 3H), 2.38 (s, 3H). ^31^P NMR (162 MHz, CDCl_3_) δ 28.70. ^13^C NMR (101 MHz, CDCl_3_) δ 163.75, 149.91, 147.80, 145.76, 141.83, 141.81, 133.11, 132.86, 132.71, 132.18, 132.07, 132.04, 131.97, 131.68, 130.63, 130.13, 129.08, 128.69, 128.64, 128.57, 128.52, 128.48, 127.07, 126.94, 125.33, 121.14, 112.52, 111.85, 56.08, 55.97, 45.00, 44.35, 42.58, 21.68. HRMS (ESI) Calculated for C_37_H_36_ClN_2_O_6_PS [M+Na]^+^: 725.1618 Found: 725.1615.

*3-chloro-N-(4-(diphenylphosphoryl)benzyl)-2-((4-methylphenyl)sulfonamido)-3-(2,3,4-trimethoxyphenyl)propenamide* ***(14e)***

White solid, 83% (182 mg); ^1^H NMR (400 MHz, Chloroform-*d*) δ 9.02 (t, *J* = 5.9 Hz, 1H), 7.60 – 7.34 (m, 14H), 7.23 – 7.13 (m, 2H), 7.07 – 6.96 (m, 3H), 6.85 – 6.77 (m, 1H), 5.09 (dd, *J* = 8.9, 6.4 Hz, 1H), 4.73 (d, *J* = 6.5 Hz, 1H), 4.35 – 4.24 (m, 3H), 3.83 (s, 3H), 3.76 (s, 3H), 3.57 (s, 3H), 2.31 (s, 3H). ^31^P NMR (162 MHz, CDCl_3_) δ 30.41. ^13^C NMR (101 MHz, CDCl_3_) δ 168.20, 150.20, 150.11, 146.78, 142.90, 137.94, 132.33, 132.26, 132.16, 132.09, 132.05, 132.00, 131.95, 129.23, 129.17, 128.84, 128.78, 128.72, 128.66, 127.76, 127.71, 127.64, 127.59, 127.10, 127.03, 126.62, 123.74, 122.56, 61.17, 61.06, 60.68, 57.30, 55.93, 43.00, 21.53. HRMS (ESI) Calculated for C_38_H_38_ClN_2_O_7_PS [M+Na]^+^: 755.1724 Found: 755.1722.

*3-(4-bromophenyl)-3-chloro-N-(4-(diphenylphosphoryl)benzyl)-2-((4-methylphenyl)sulfonamido)propenamide* ***(14f)***

White solid, 83% (182 mg); ^1^H NMR (400 MHz, Chloroform-*d*) δ 8.78 (t, *J* = 5.7 Hz, 1H), 7.61 – 7.38 (m, 13H), 7.29 – 7.20 (m, 2H), 7.16 – 7.07 (m, 4H), 7.05 – 6.98 (m, 3H), 6.74 (d, *J* = 10.1 Hz, 1H), 5.08 (d, *J* = 8.1 Hz, 1H), 4.65 (dd, *J* = 10.3, 8.1 Hz, 1H), 4.24 (ddd, *J* = 53.3, 15.5, 5.2 Hz, 2H), 2.34 (s, 3H). ^31^P NMR (162 MHz, CDCl_3_) δ 30.42. ^13^C NMR (101 MHz, CDCl_3_) δ 169.01, 143.25, 142.40, 142.37, 137.19, 136.41, 132.51, 132.48, 132.31, 132.21, 132.15, 132.05, 131.39, 129.91, 129.37, 128.92, 128.89, 128.80, 128.77, 128.14, 128.01, 127.33, 127.08, 122.82, 62.65, 60.76, 43.41, 21.66. HRMS (ESI) Calculated for C_35_H_31_BrClN_2_O_4_PS [M+Na]^+^: 743.0512 Found: 743.0507.

*3-chloro-N-(4-(diphenylphosphoryl)benzyl)-3-(3-fluorophenyl)-2-((4-methylphenyl)sulfonamido)propenamide* ***(14g)***

^1^H NMR (400 MHz, Chloroform-*d*) δ 8.86 (t, *J* = 5.5 Hz, 1H), 7.68 – 7.36 (m, 13H), 7.31 – 7.22 (m, 2H), 7.20 – 7.09 (m, 2H), 7.04 – 6.94 (m, 3H), 6.93 – 6.80 (m, 2H), 6.72 (d, *J* = 10.1 Hz, 1H), 5.12 (d, *J* = 7.9 Hz, 1H), 4.67 (dd, *J* = 10.0, 7.9 Hz, 1H), 4.36 – 4.09 (m, 2H), 2.29 (s, 3H). ^31^P NMR (162 MHz, CDCl_3_) δ 30.39. ^13^C NMR (101 MHz, CDCl_3_) δ 168.88, 163.68, 161.23, 144.51, 143.24, 142.43, 142.41, 139.71, 139.64, 137.11, 132.45, 132.42, 132.32, 132.31, 132.21, 132.15, 132.12, 132.05, 132.02, 131.25, 129.91, 129.83, 129.73, 129.64, 129.42, 129.37, 128.89, 128.76, 128.73, 128.02, 127.89, 127.66, 127.41, 127.08, 124.14, 124.11, 115.82, 115.61, 115.34, 115.11, 62.69, 60.82, 43.32, 21.55. HRMS (ESI) Calculated for C_35_H_31_ClFN_2_O_4_PS [M+Na]^+^: 683.1312 Found: 683.1310.

*3-(4-bromo-2-fluorophenyl)-3-chloro-N-(4-(diphenylphosphoryl)benzyl)-2-((4-methylphenyl)sulfonamido)propenamide* ***(14h)***

^1^H NMR (400 MHz, Chloroform-*d*) δ 9.23 (t, *J* = 5.2 Hz, 1H), 7.62 – 7.36 (m, 14H), 7.30 – 7.08 (m, 4H), 6.97 (d, *J* = 8.1 Hz, 2H), 6.61 (td, *J* = 8.3, 2.7 Hz, 1H), 6.49 (d, *J* = 10.2 Hz, 1H), 5.54 (d, *J* = 8.8 Hz, 1H), 4.90 (dd, *J* = 10.5, 8.9 Hz, 1H), 4.31 (dd, *J* = 15.5, 5.6 Hz, 1H), 4.04 (dd, *J* = 15.4, 4.7 Hz, 1H), 2.30 (s, 3H). ^31^P NMR (162 MHz, CDCl_3_) δ 30.41. ^13^C NMR (101 MHz, CDCl_3_) δ 168.56, 143.21, 142.40, 142.37, 137.19, 132.49, 132.31, 132.26, 132.16, 132.06, 131.22, 129.46, 129.34, 128.92, 128.79, 128.21, 128.08, 127.49, 127.35, 127.02, 124.38, 124.28, 119.72, 119.50, 115.24, 115.02, 61.80, 58.97, 43.50, 21.56. HRMS (ESI) Calculated for C_35_H_30_BrClFN_2_O_4_PS [M+Na]^+^: 761.0418 Found: 761.0415.

*3-chloro-3-(3-chloro-4-nitrophenyl)-N-(4-(diphenylphosphoryl)benzyl)-2-((4-methylphenyl)sulfonamido)propenamide* ***(14j)***

^1^H NMR (400 MHz, Chloroform-*d*) δ 8.98 (t, *J* = 5.0 Hz, 1H), 7.74 – 7.32 (m, 14H), 7.30 – 7.05 (m, 6H), 7.03 – 6.96 (m, 2H), 5.12 (d, *J* = 8.4 Hz, 1H), 4.69 (dd, *J* = 10.2, 8.4 Hz, 1H), 4.37 – 4.12 (m, 2H), 2.32 (s, 3H). ^31^P NMR (162 MHz, CDCl_3_) δ 31.14. ^13^C NMR (101 MHz, CDCl_3_) δ 168.61, 147.12, 143.57, 142.41, 138.15, 137.16, 133.01, 132.66, 132.37, 132.27, 132.14, 132.04, 131.83, 130.98, 129.49, 129.46, 129.03, 128.91, 128.16, 128.04, 127.89, 127.76, 127.31, 127.21, 126.98, 125.58, 62.62, 59.47, 43.46, 21.67. HRMS (ESI) Calculated for C_35_H_30_Cl_2_N_3_O_6_PS [M+H]^+^: 722.1048 Found: 722.1040.

# General procedure for deprotection of GAP auxiliary BnDpp

To a stirred solution **13a** or **14a** (0.1 g, 0.16 mmol) and 10 wt% Pd/C (10 mg) in MeOH (1 mL) was added NaBH_4_ (7.6 mg, 2 equiv.). The 10 mL flask was closed with a rubber septum with an empty (deflated) balloon to avoid the loss of generated hydrogen and overpressure in the flask. After 2 h, the reaction mixture was filtered through Celite and filtrate was evaporated to dryness and re-dissolved in EtOAc. Then the reaction mixture was neutralized with KHSO_4_. The organic layer was separated, dried over anhydrous Na_2_SO_4_ and evaporated to dryness to afford crude GAP auxiliary, which was easily purified using the GAP washing method.

*3-chloro-2-((4-methylphenyl)sulfonamido)-3-phenylpropanamide (15a)*

^1^H NMR (400 MHz, DMSO-*d*_6_) δ 8.12 (d, *J* = 9.9 Hz, 1H), 7.72 (d, *J* = 2.3 Hz, 1H), 7.62 (d, *J* = 8.0 Hz, 1H), 7.41 (d, *J* = 8.1 Hz, 2H), 7.36 – 7.23 (m, 5H), 7.19 (d, *J* = 7.7 Hz, 2H), 4.95 (d, *J* = 9.8 Hz, 1H), 4.39 – 4.26 (m, 1H), 2.34 (s, 3H). ^13^C NMR (101 MHz, DMSO) δ 169.95, 142.18, 138.26, 137.76, 129.16, 129.10, 128.23, 128.19, 126.32, 63.90, 60.83, 20.98.

# **NMR Spectra**

#
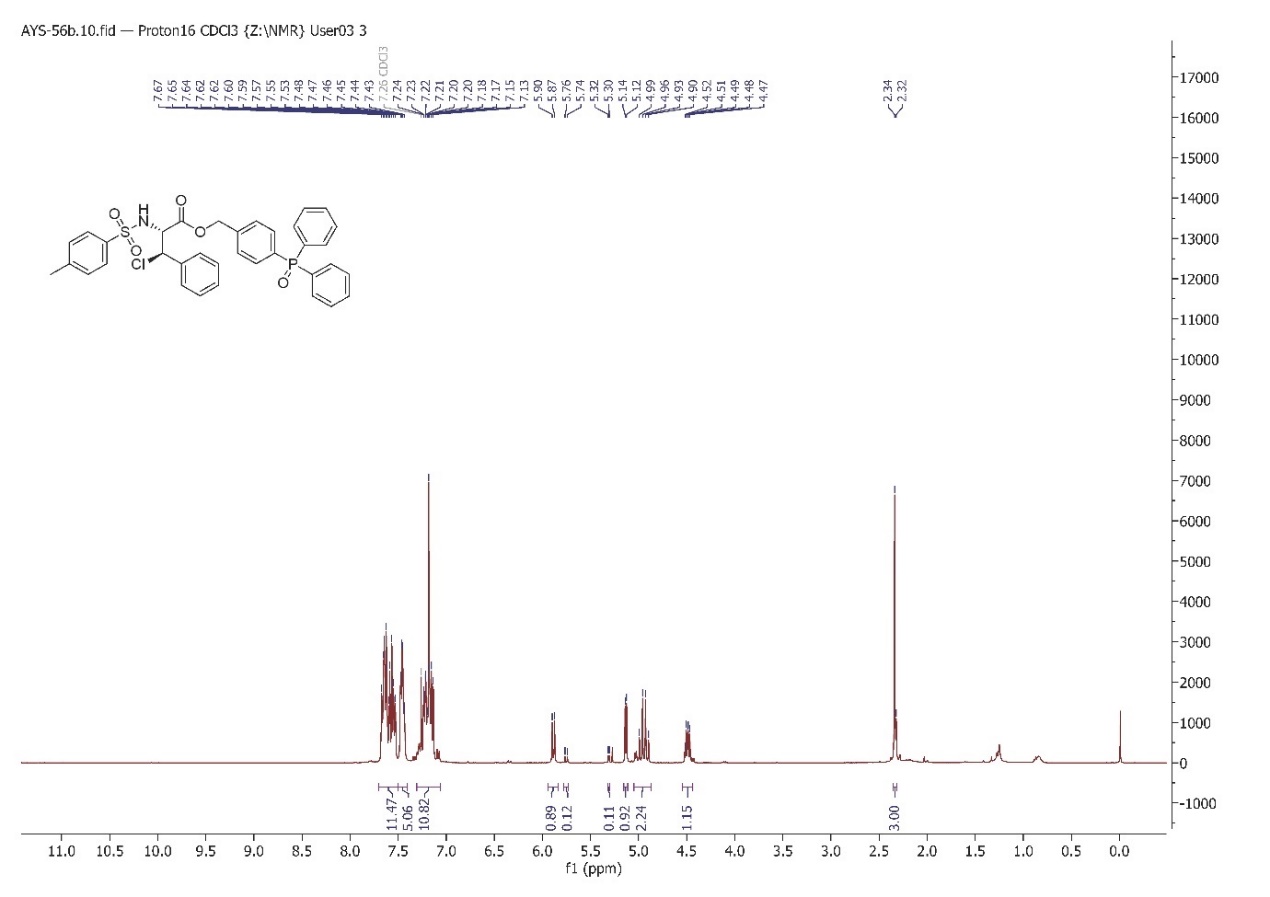

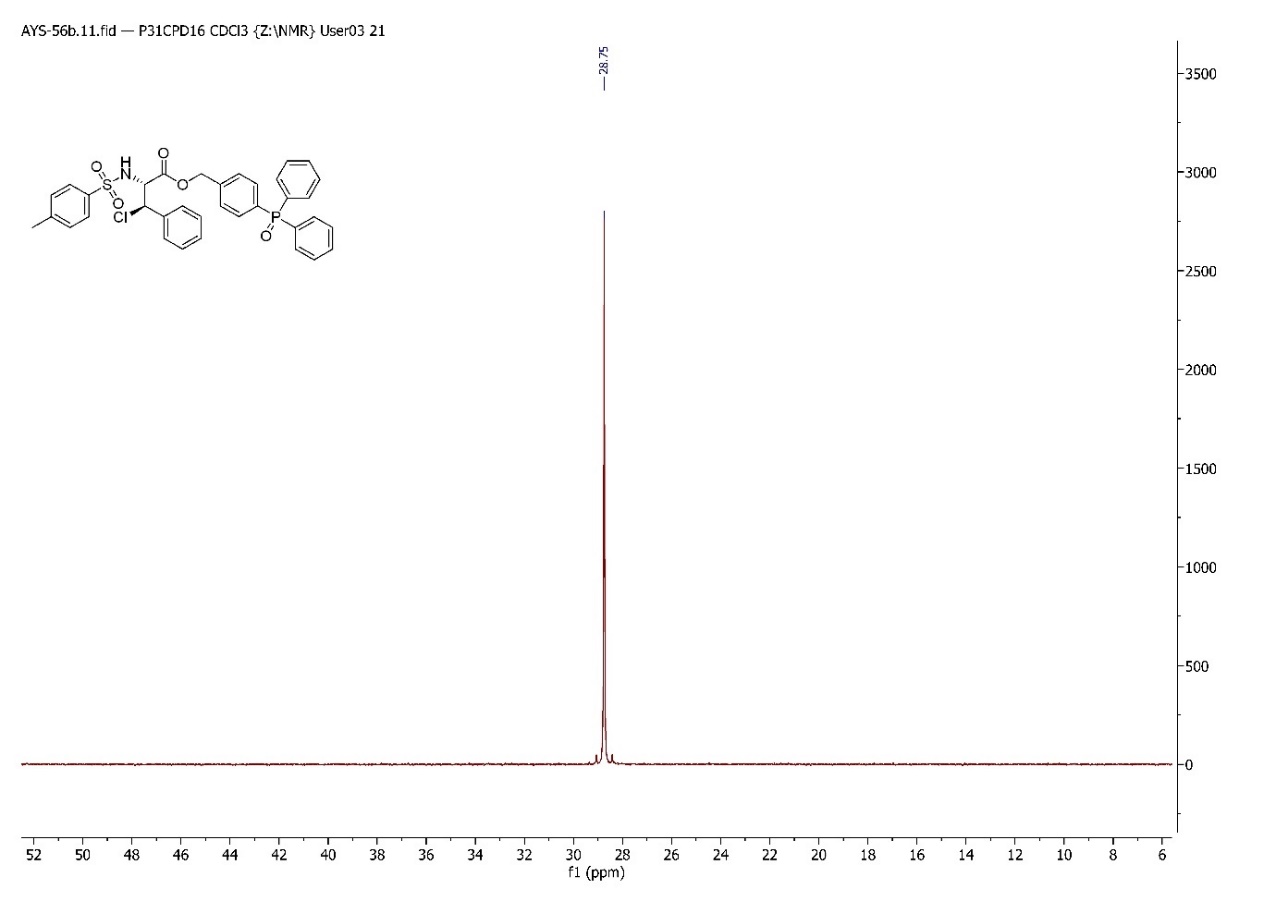

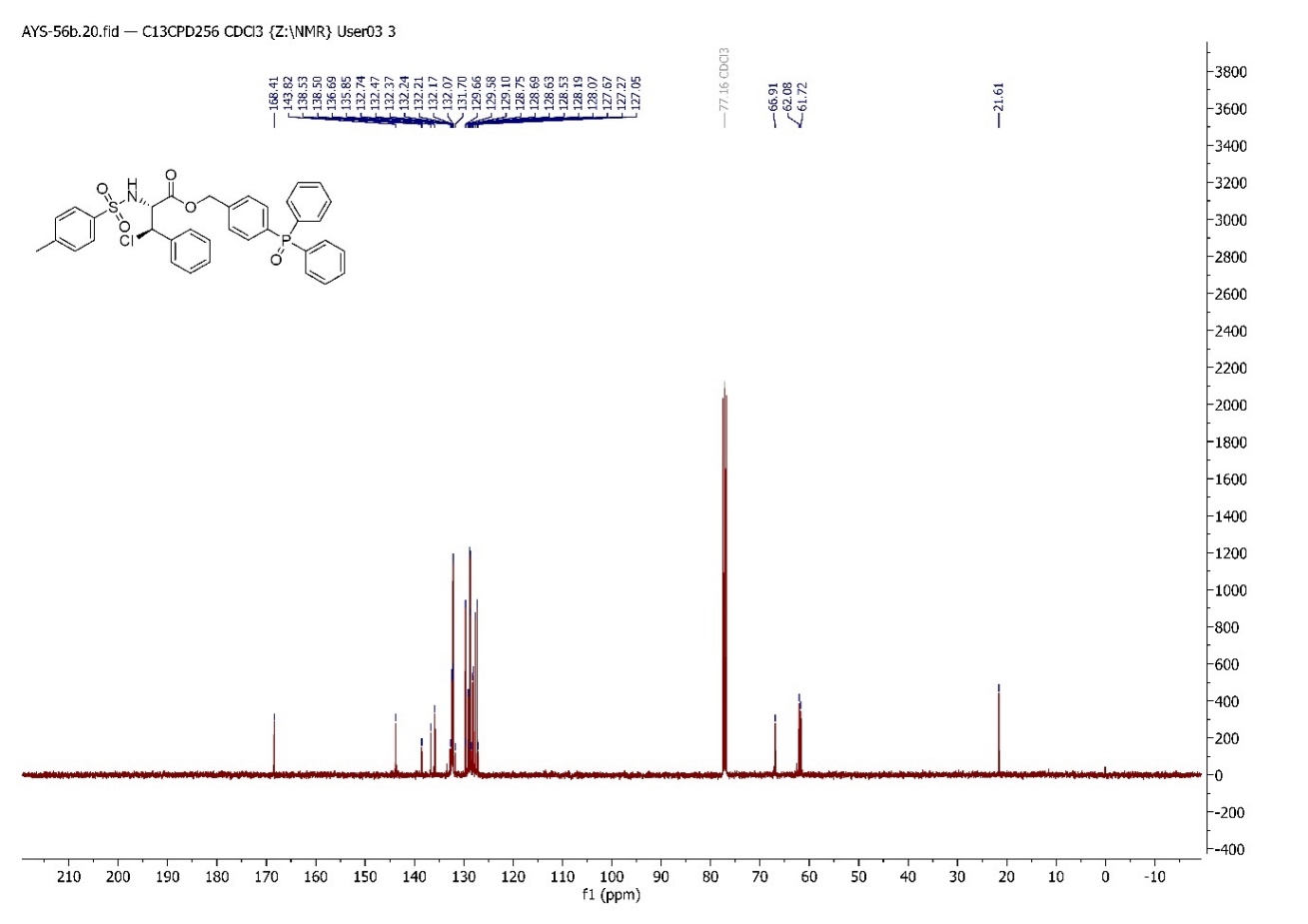

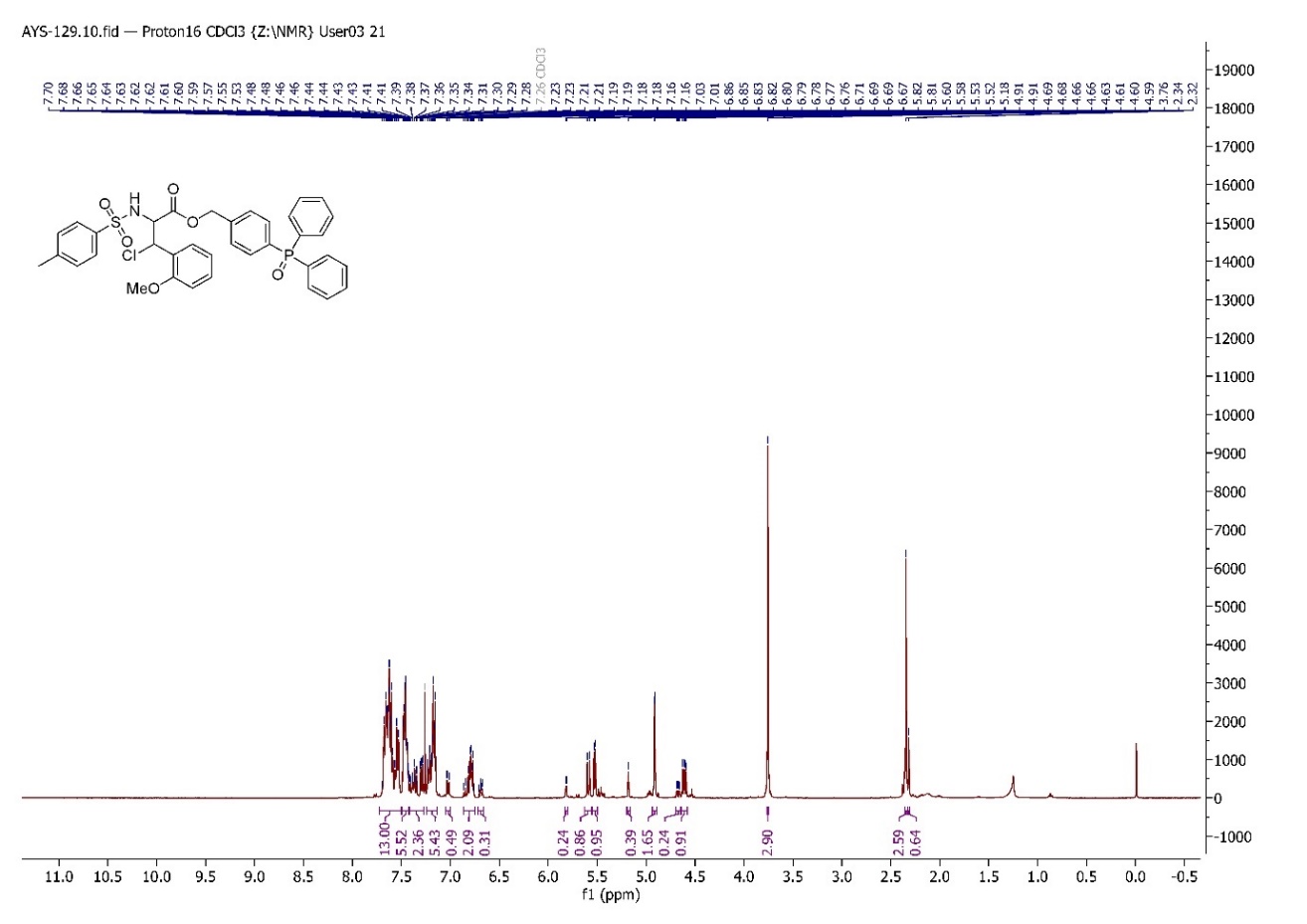

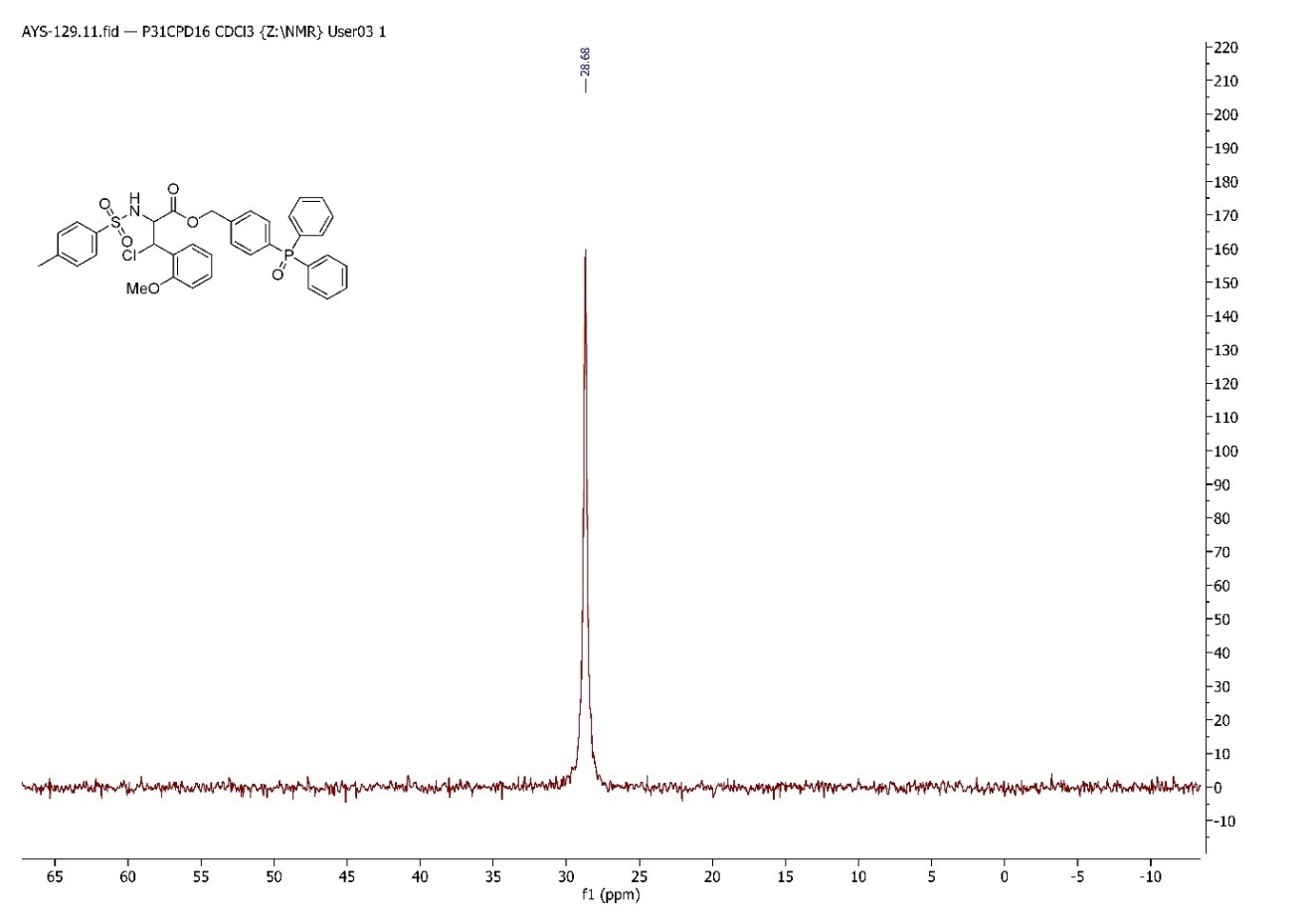

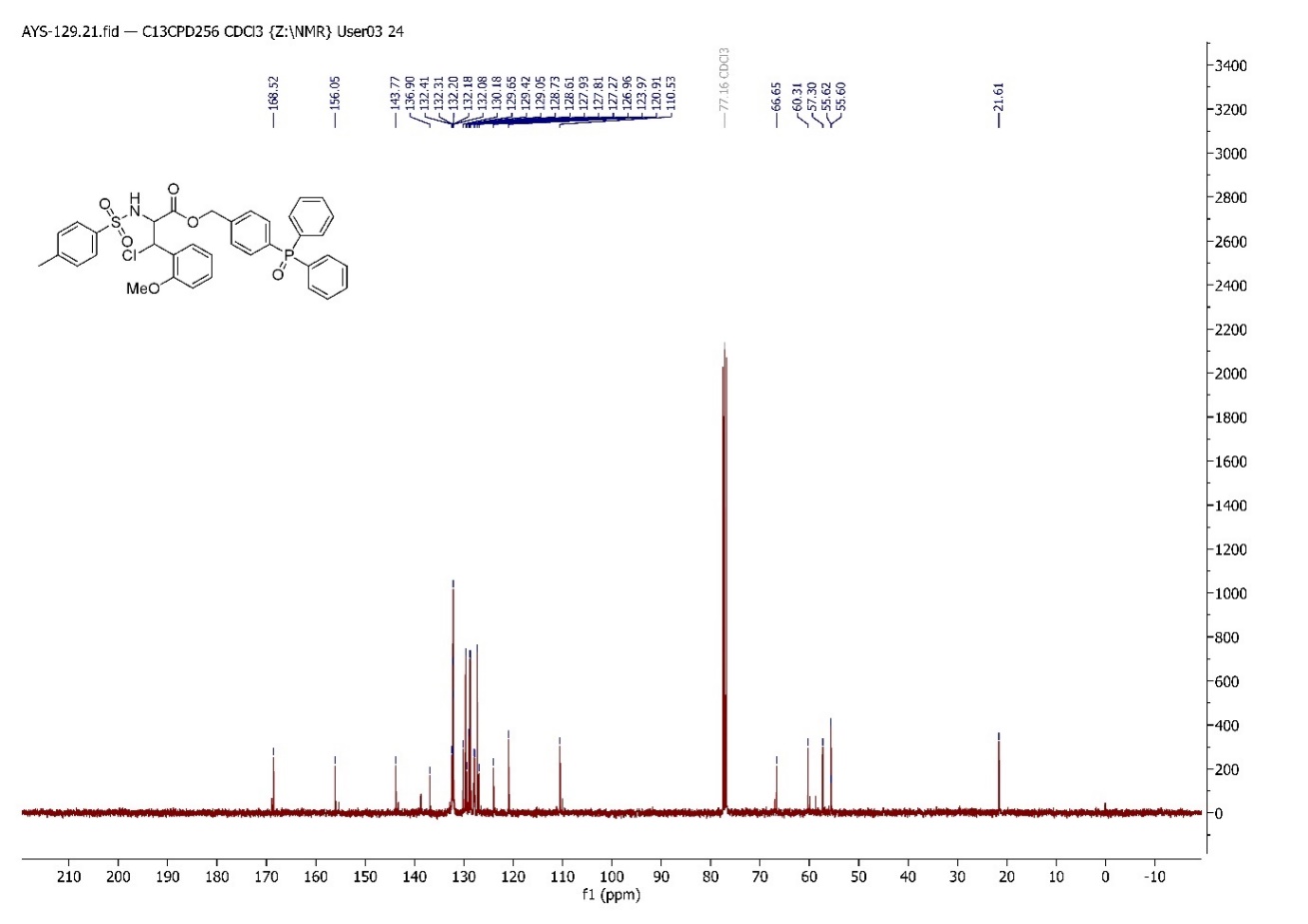

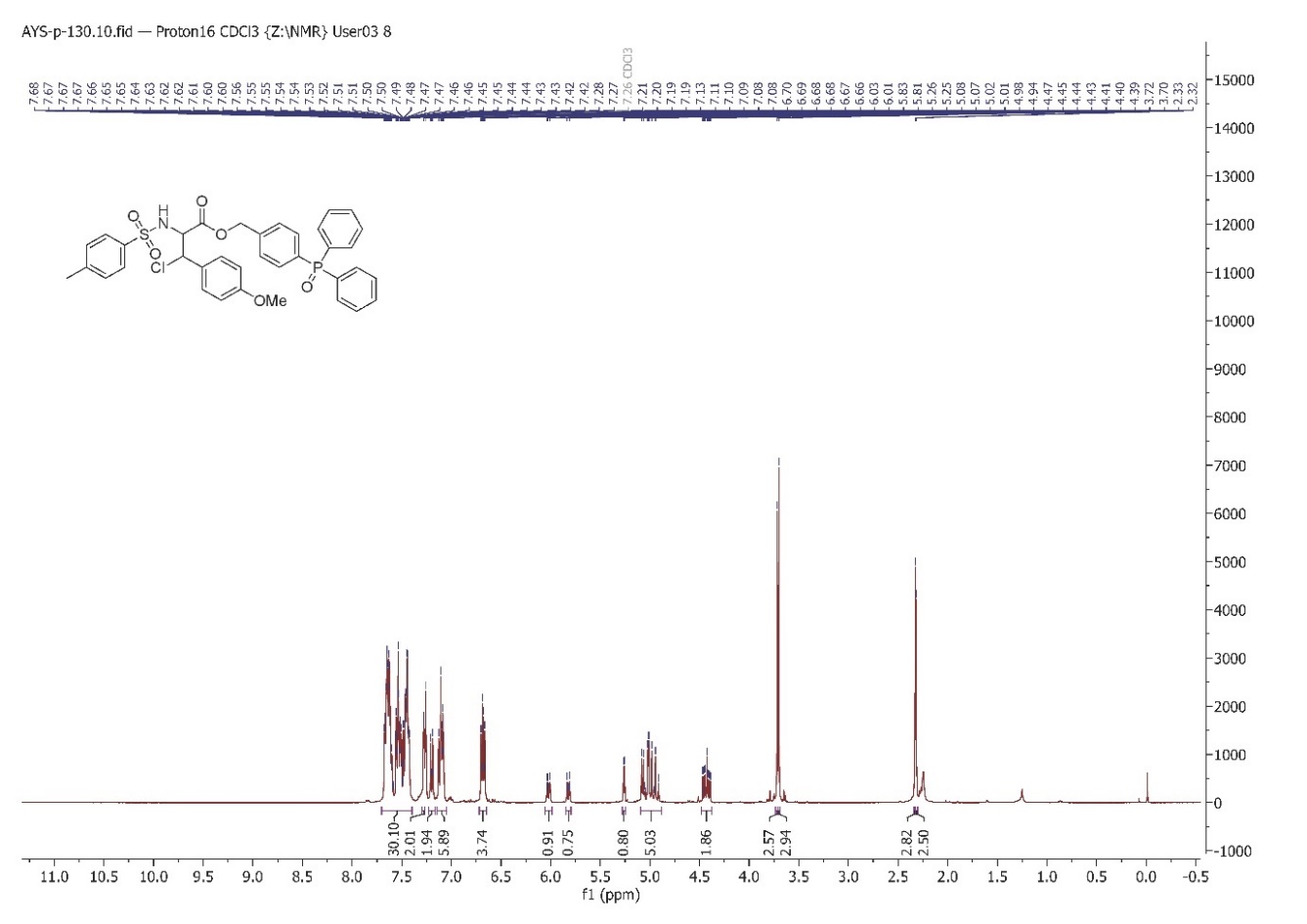

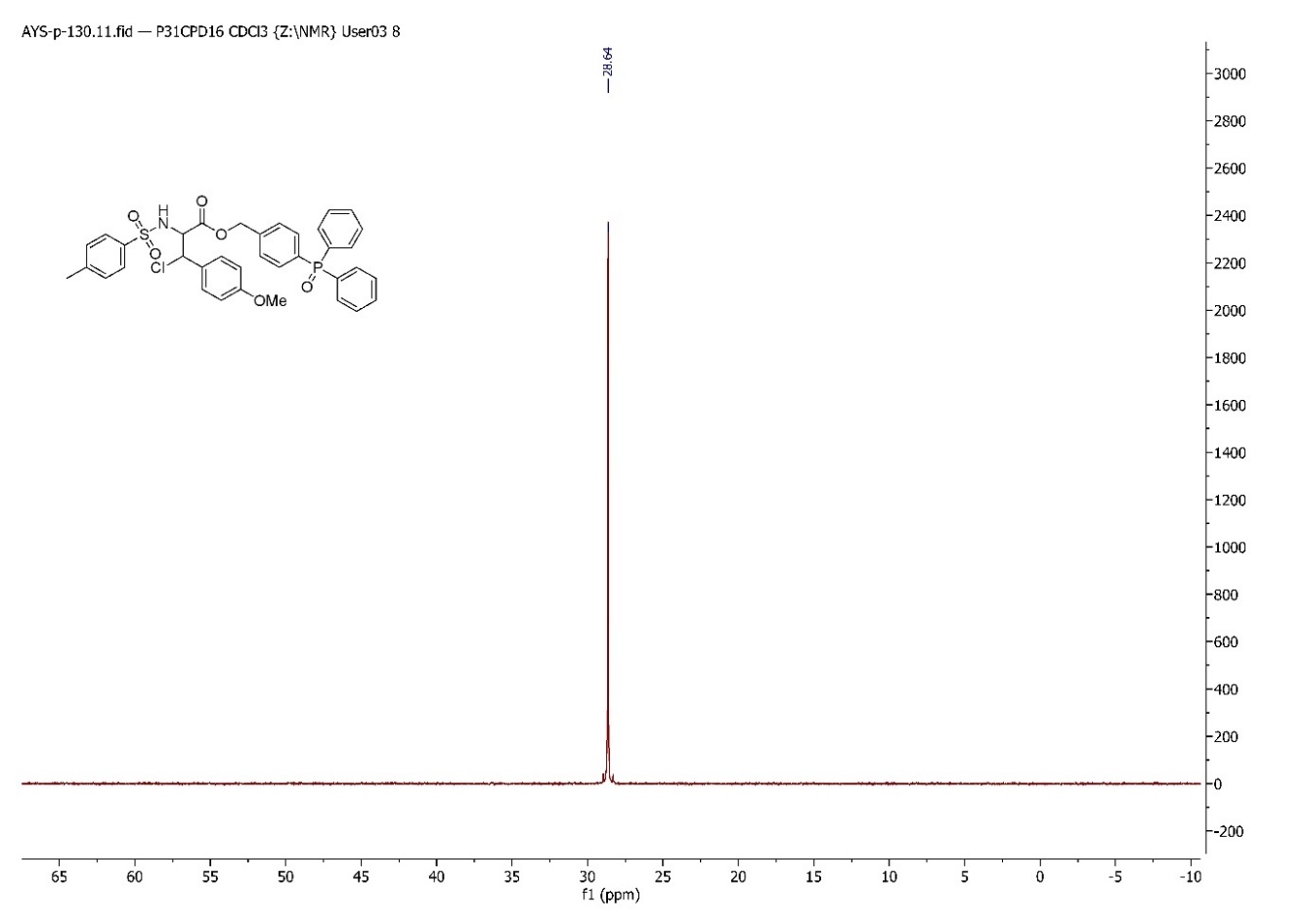

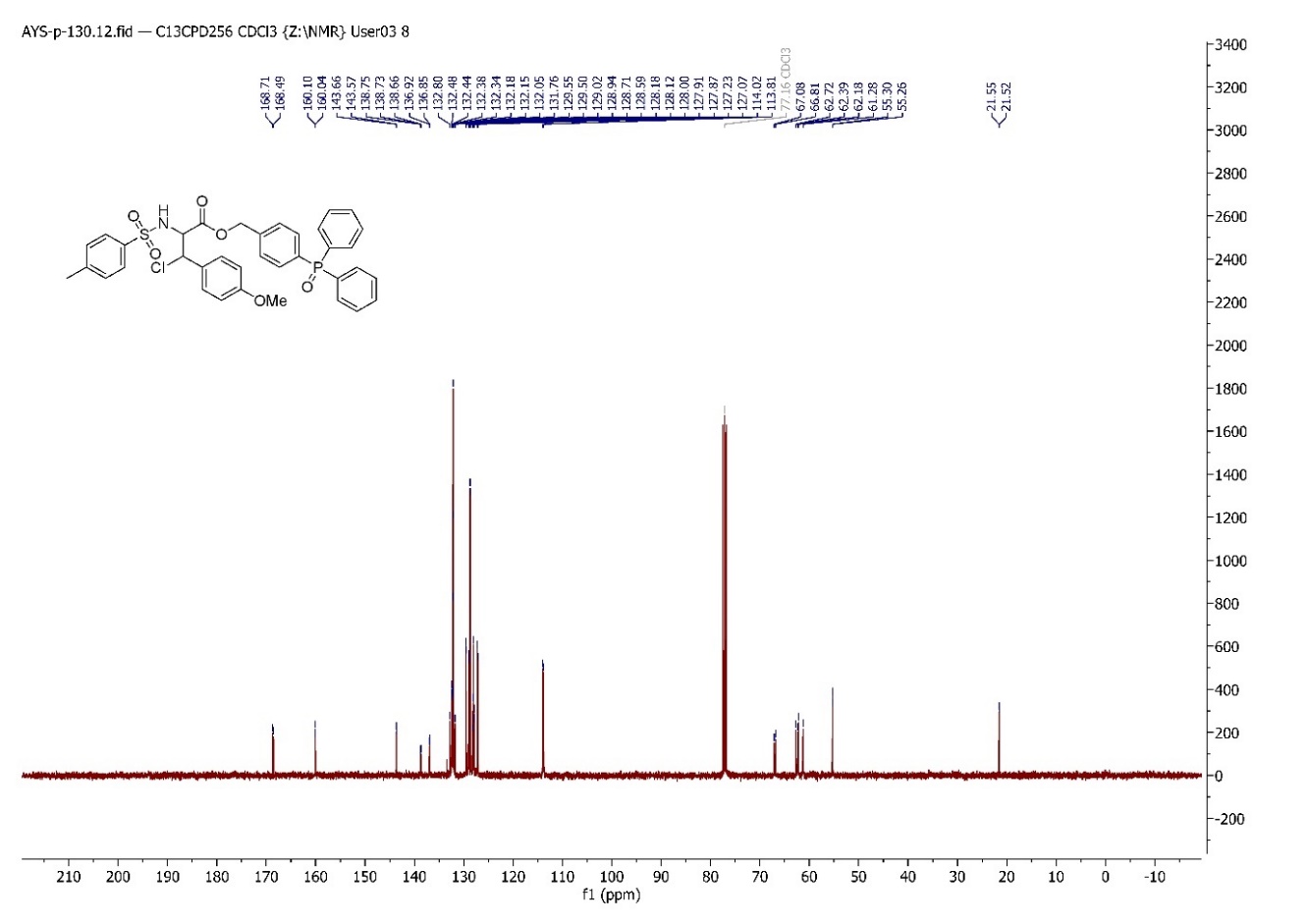

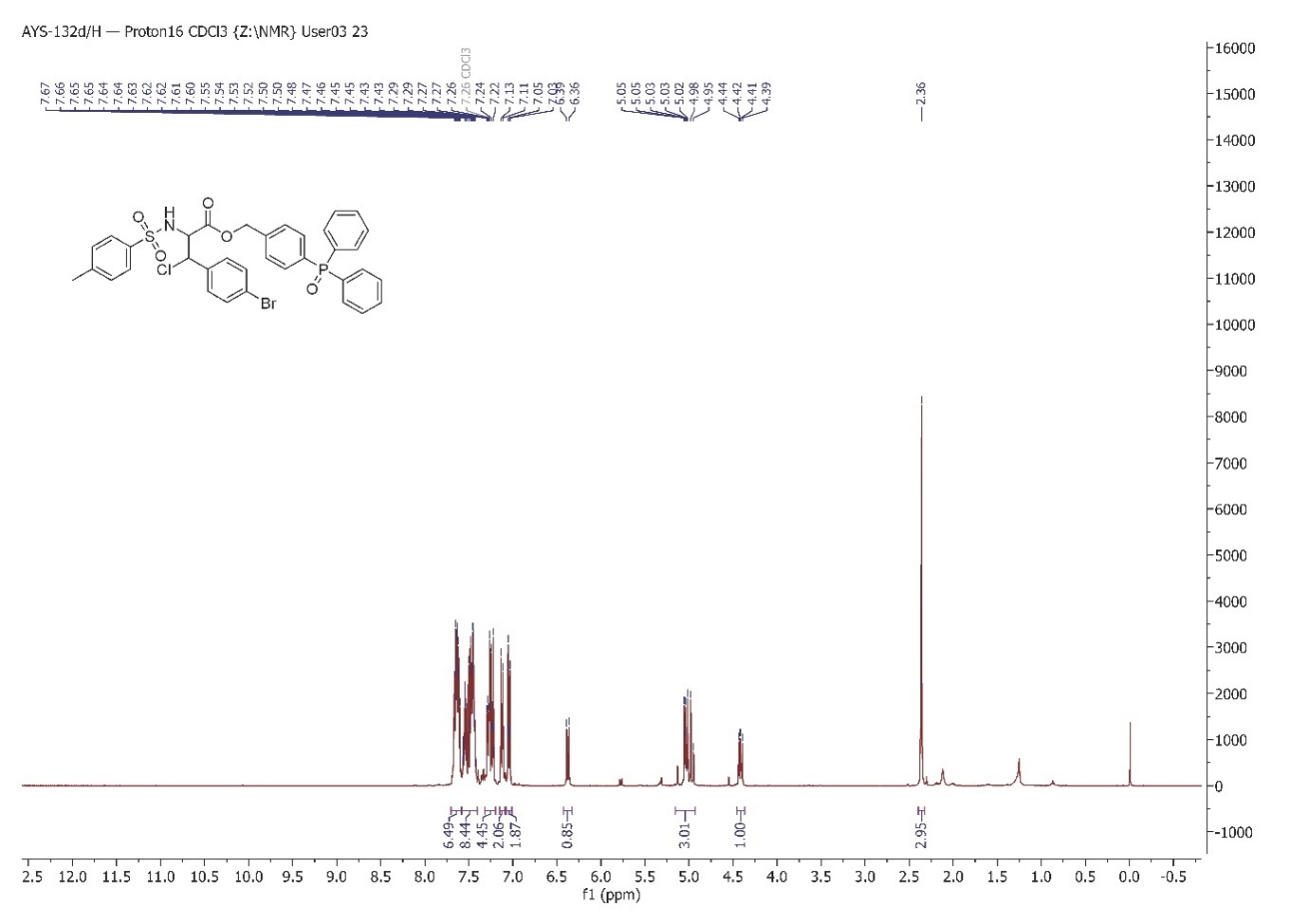

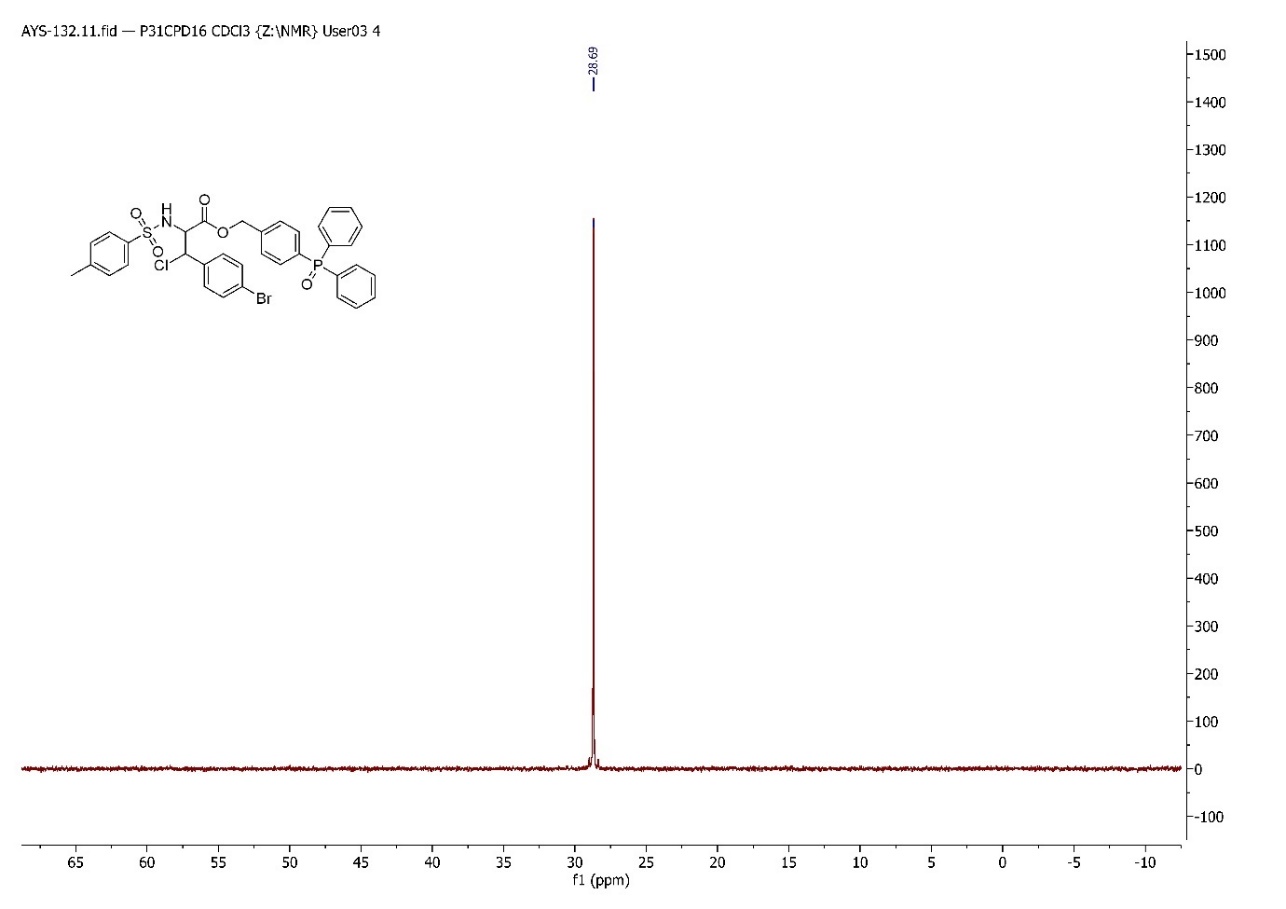

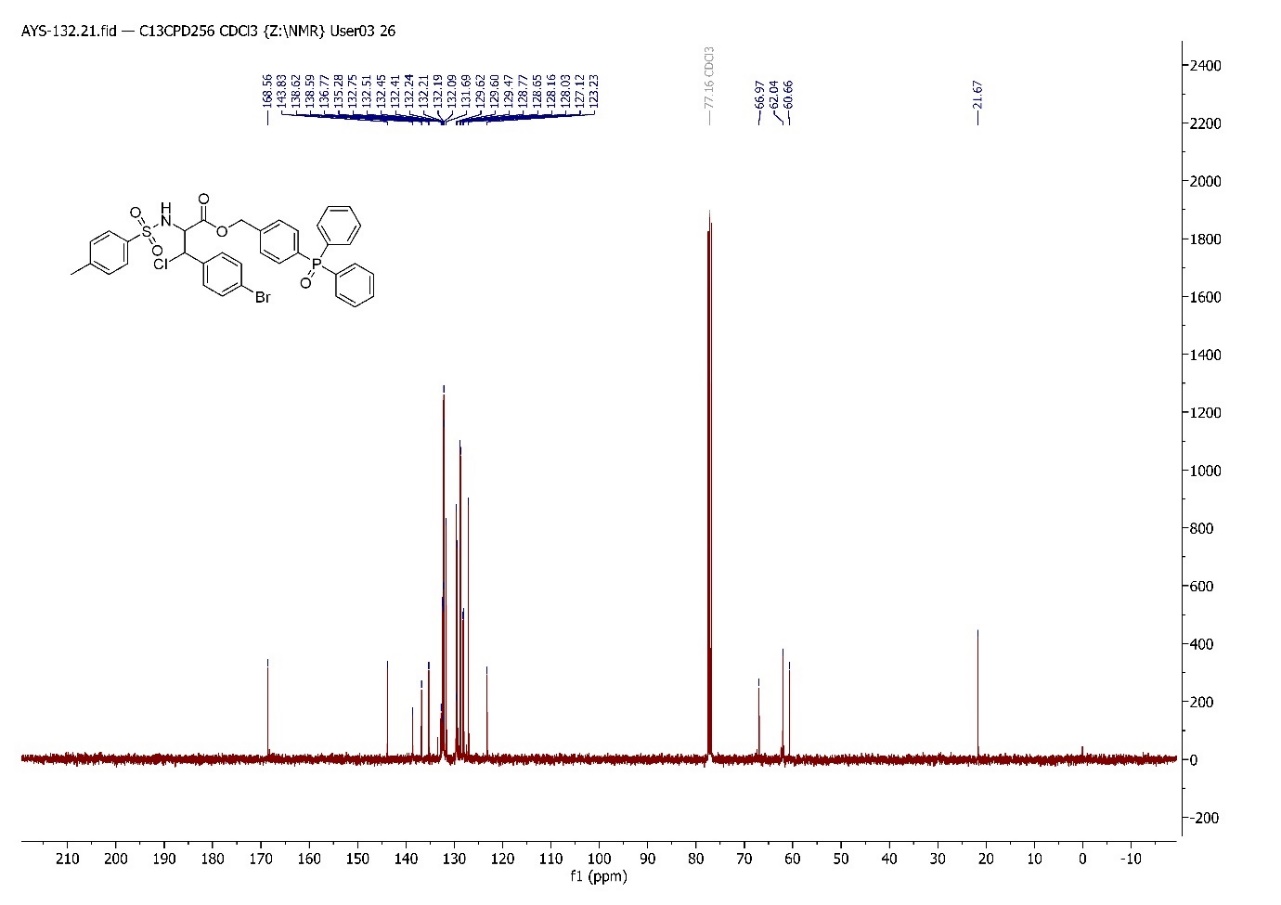


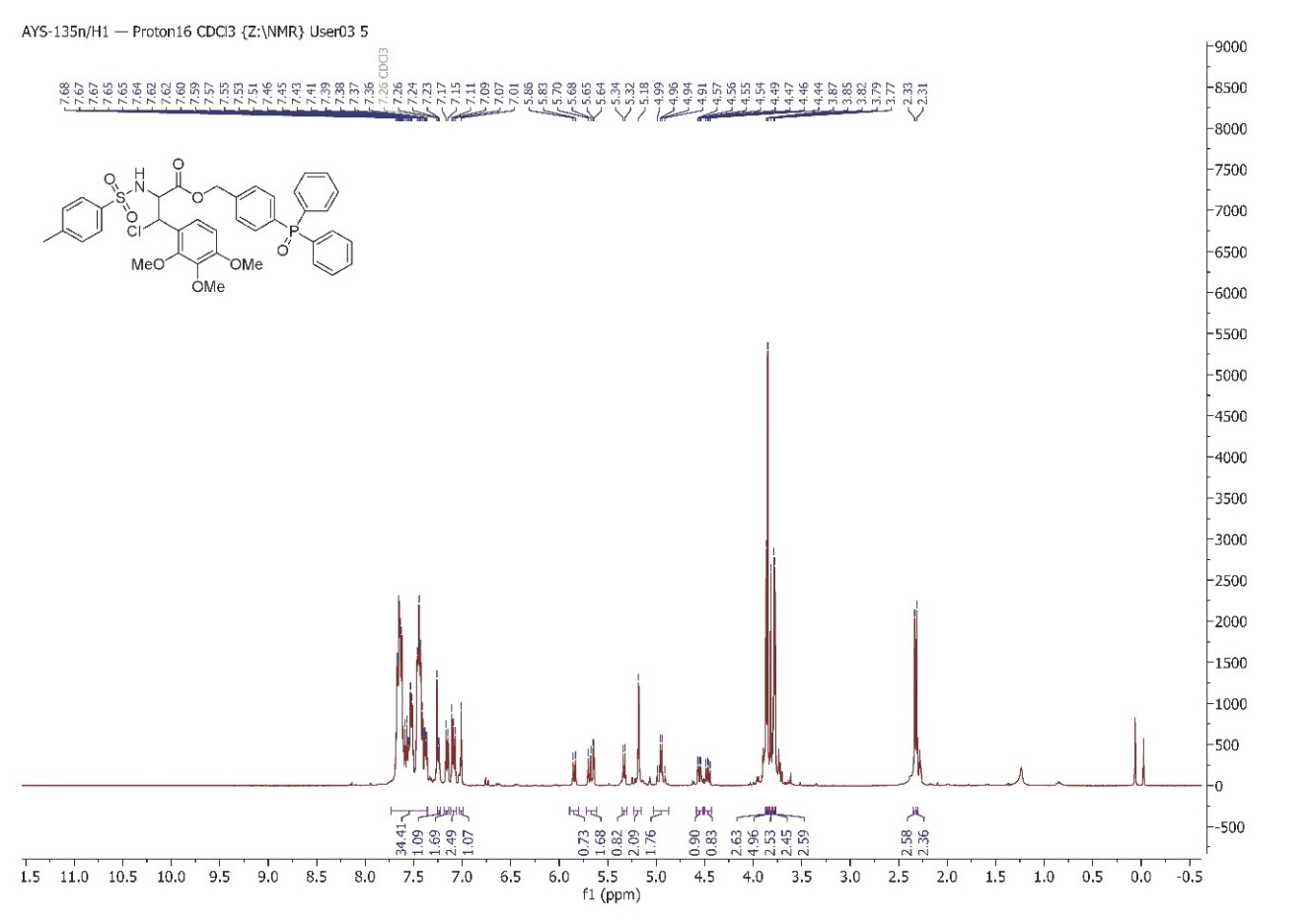

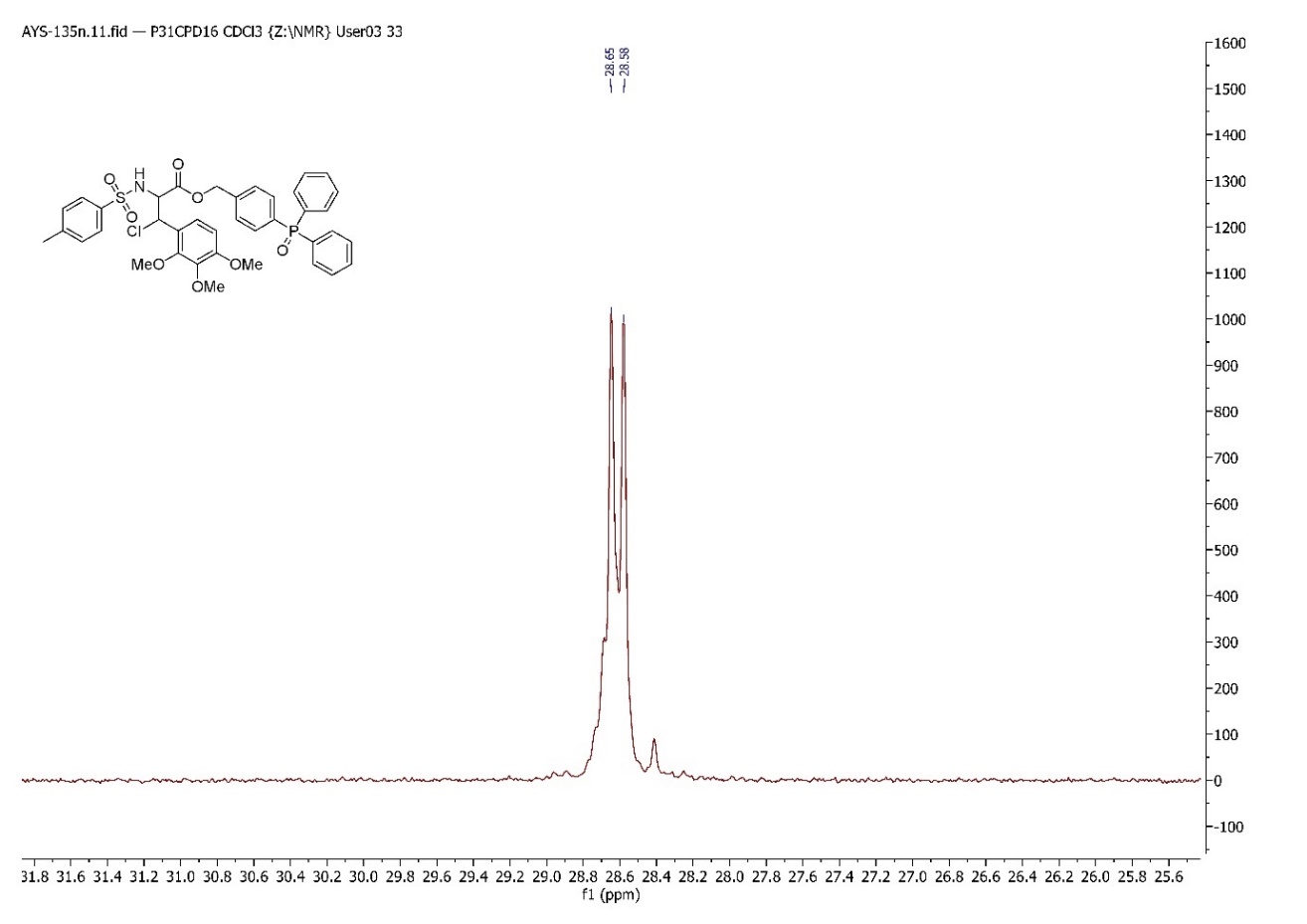

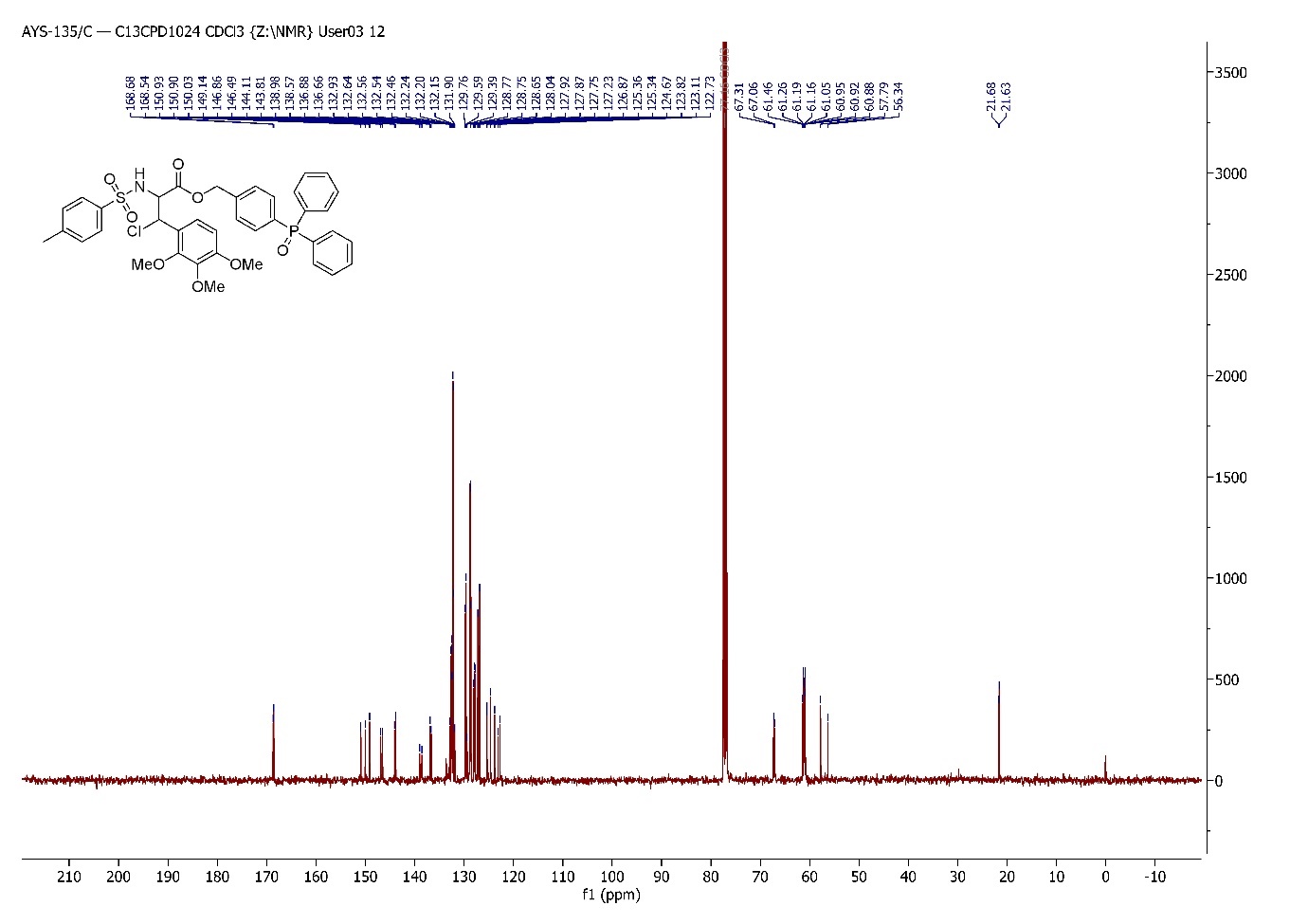

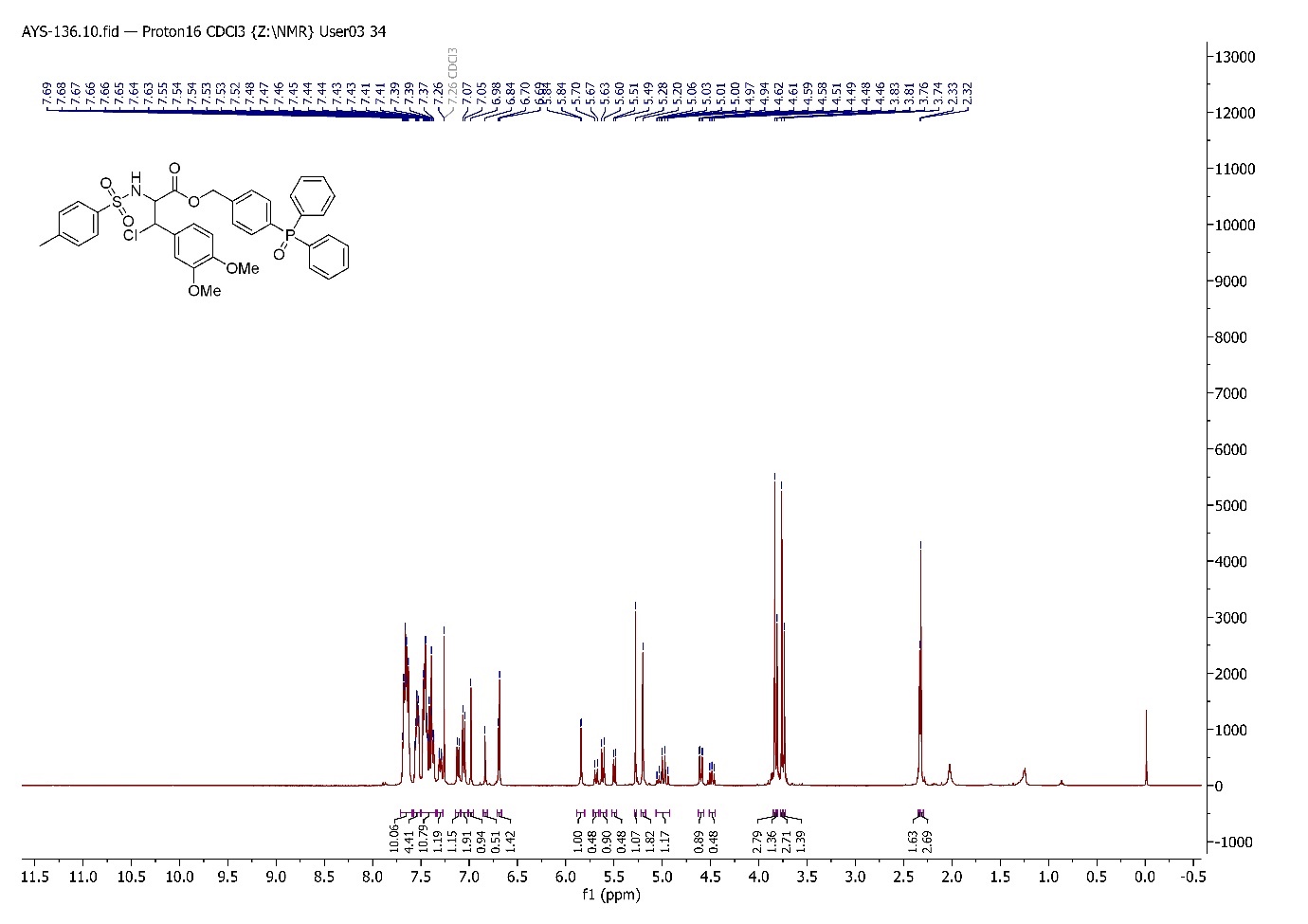

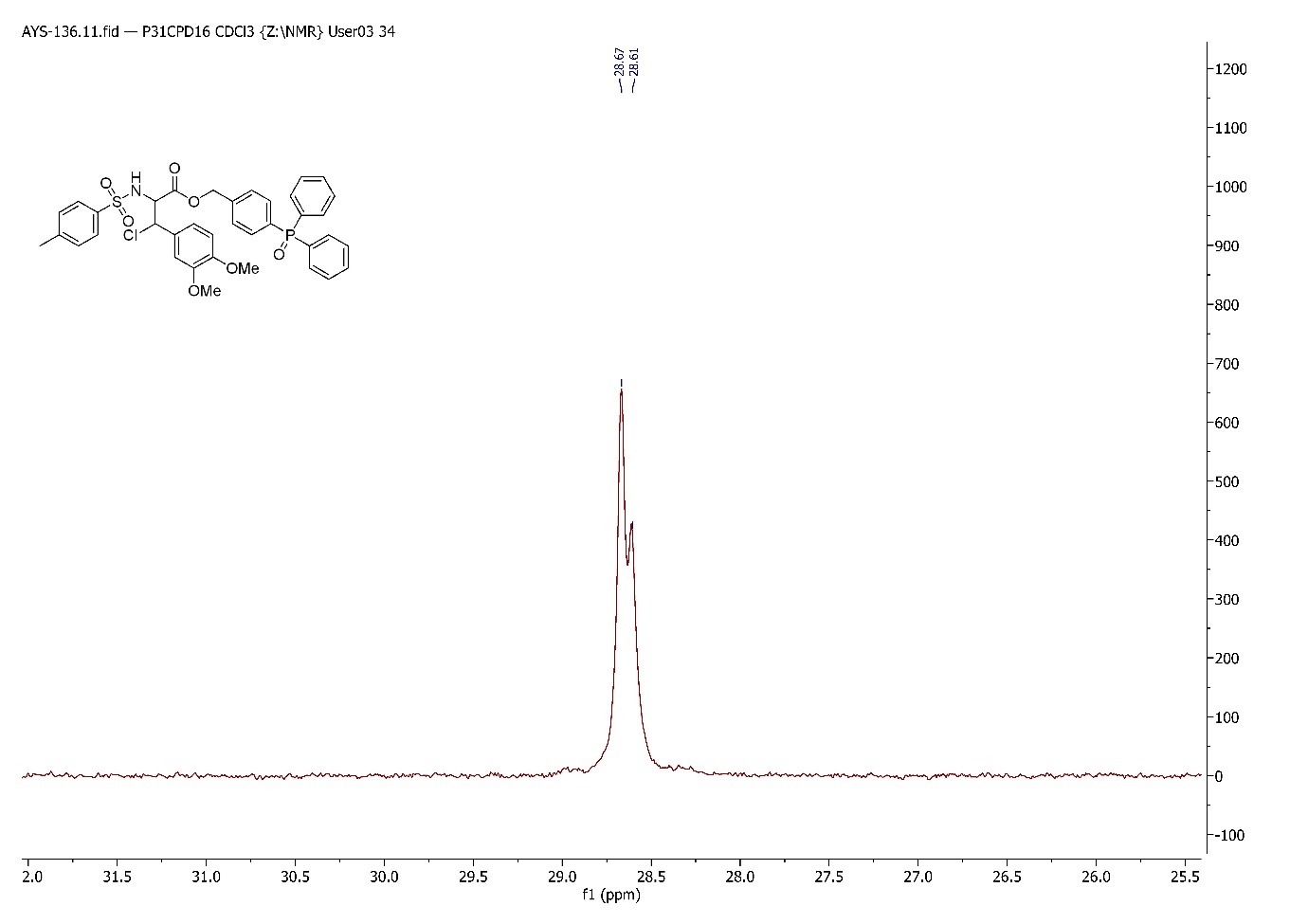

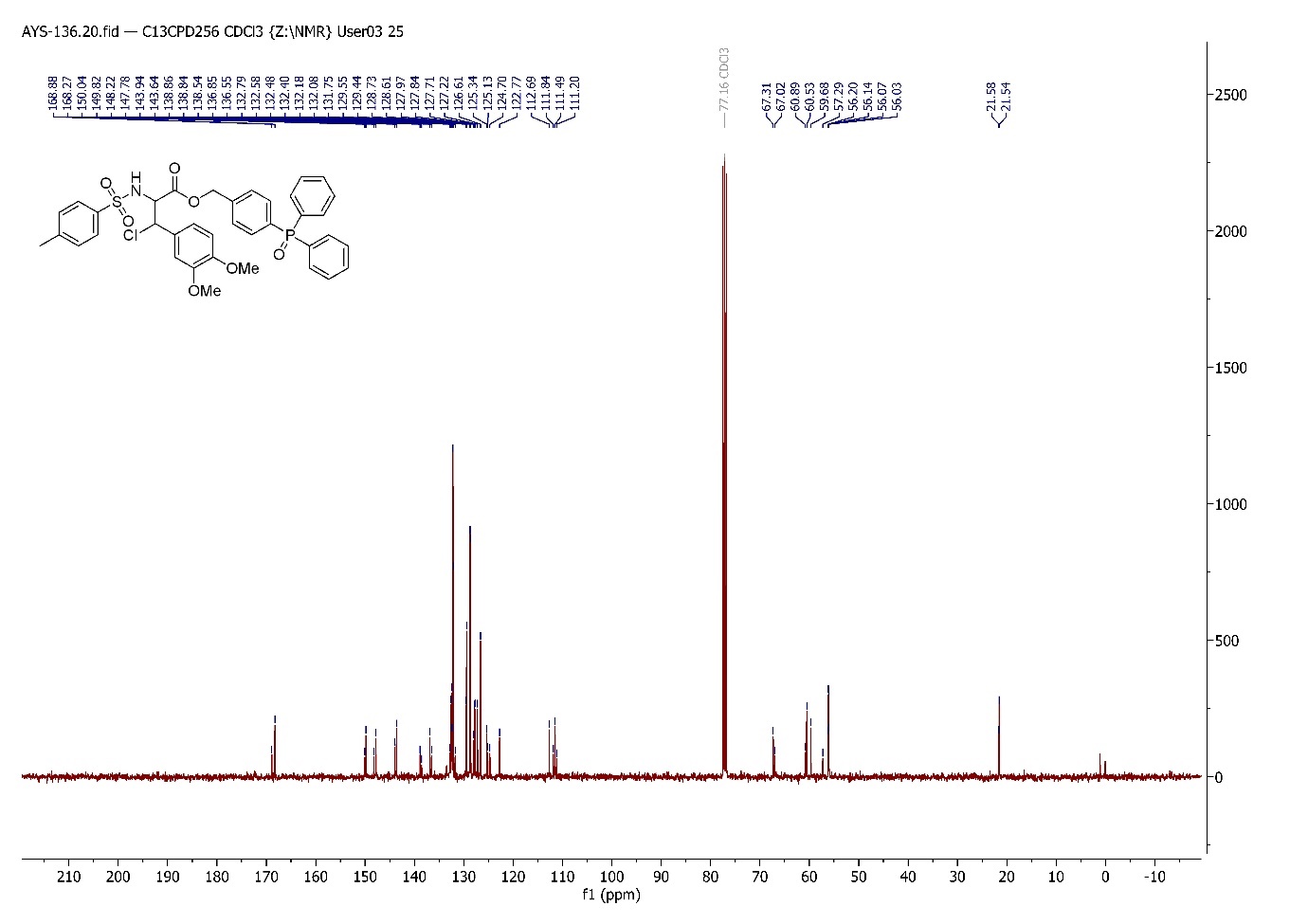


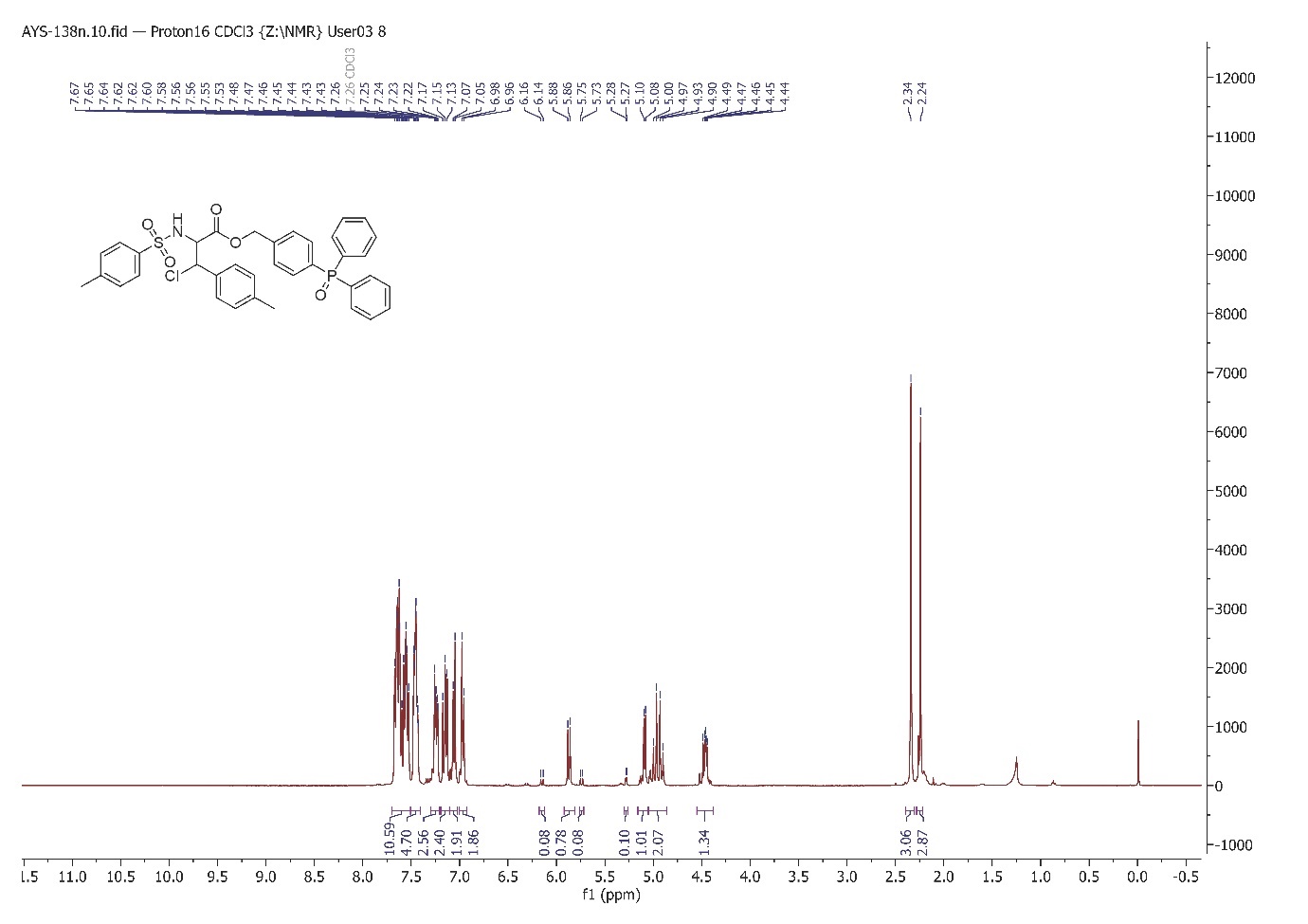

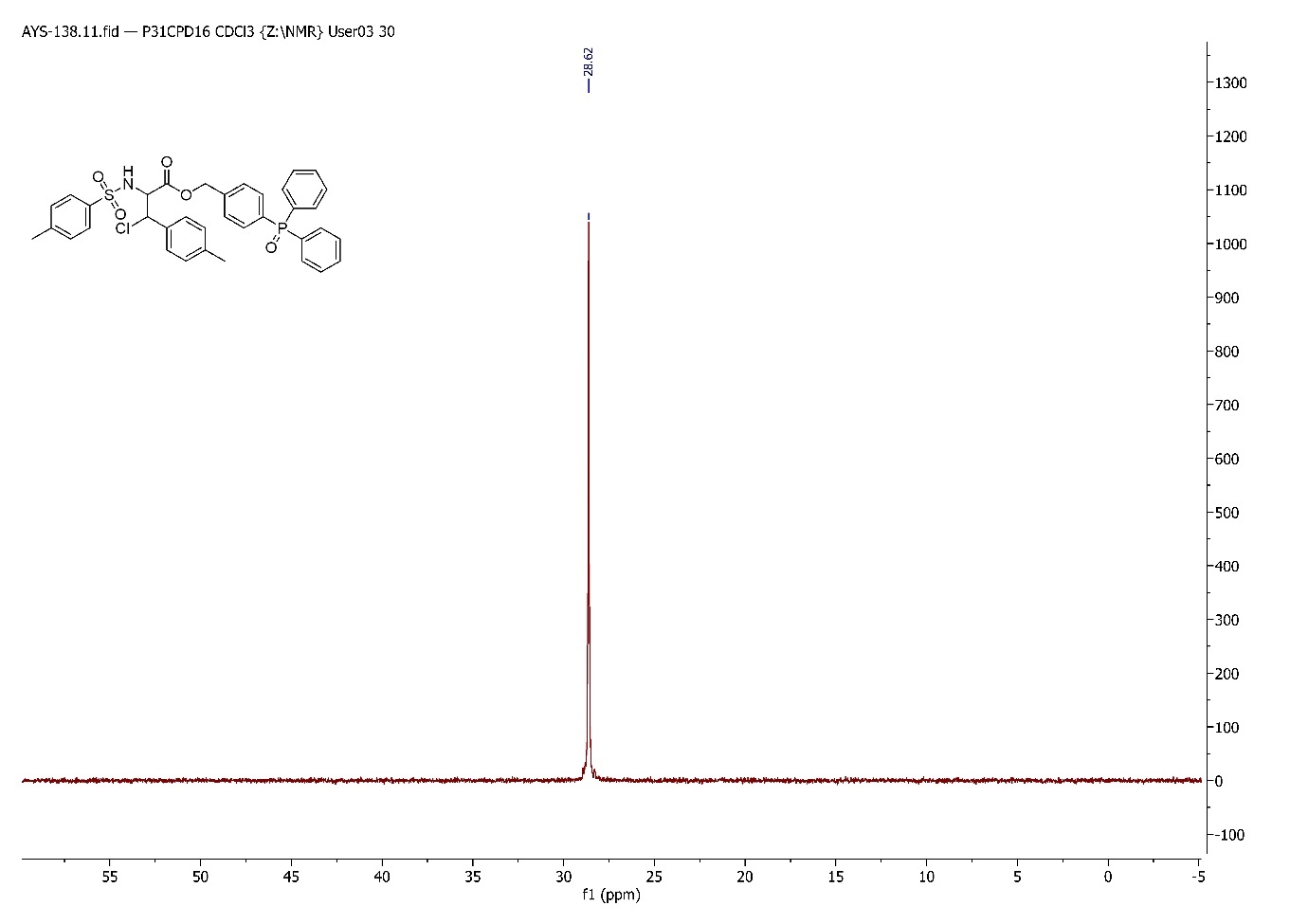

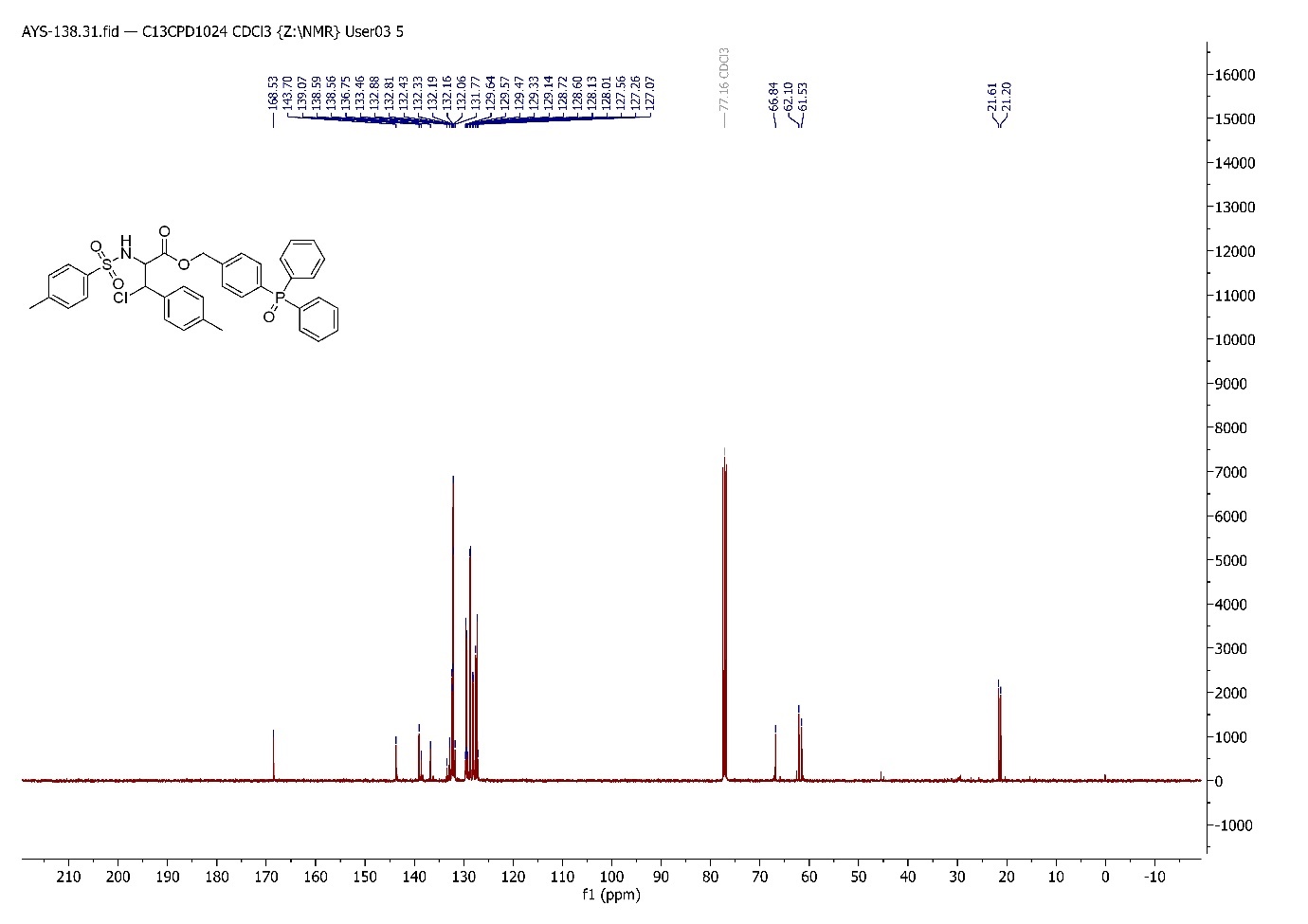

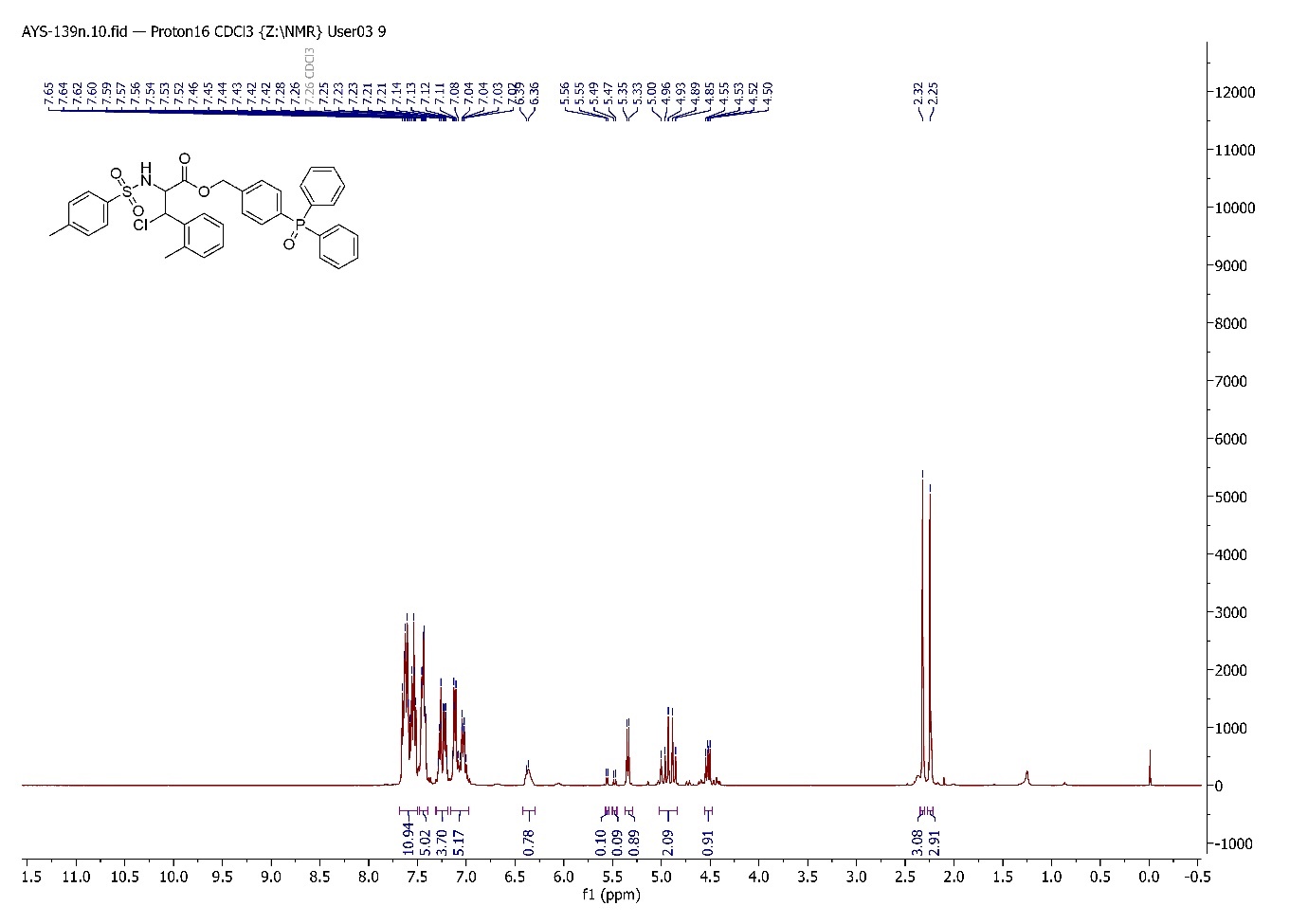

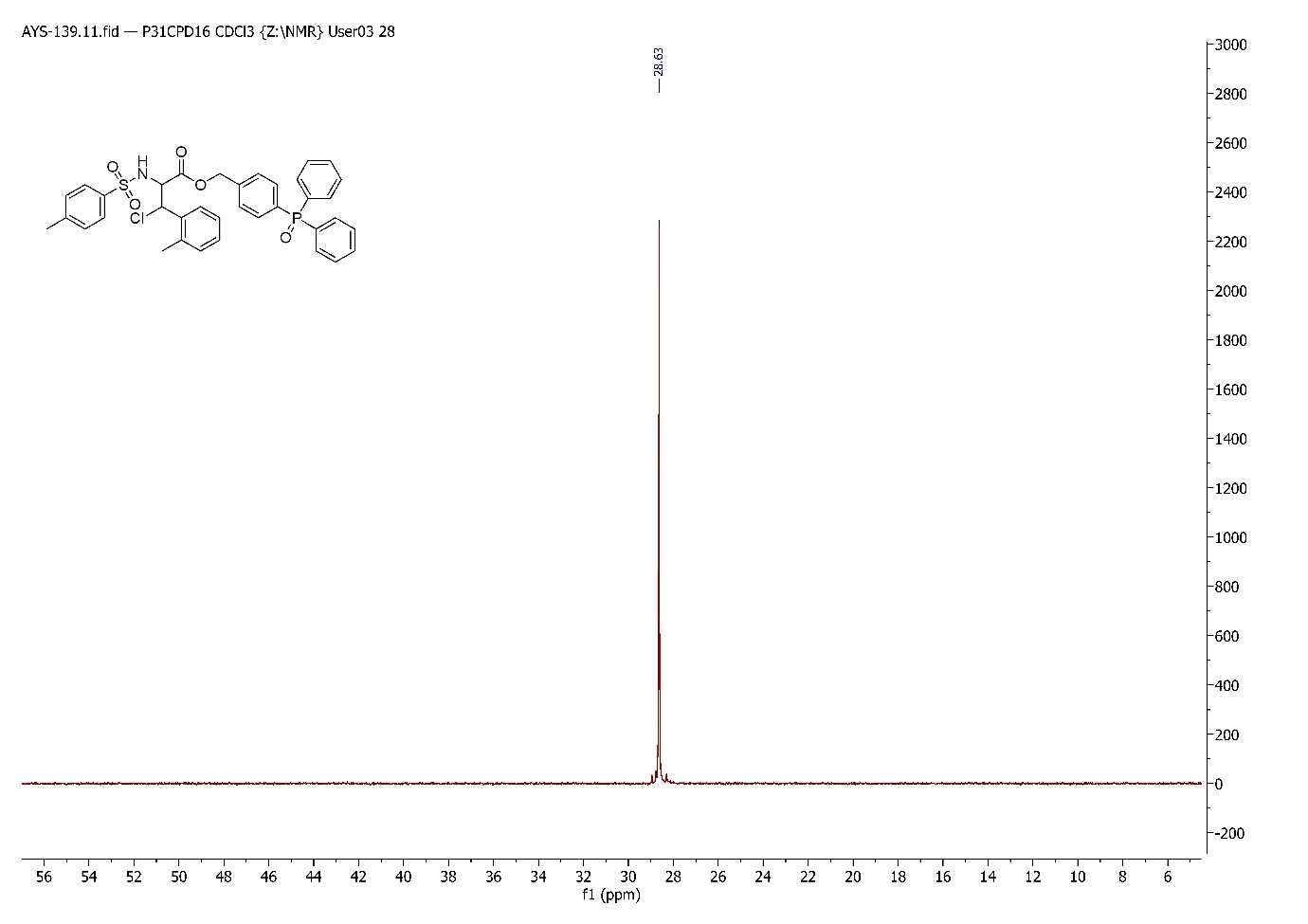

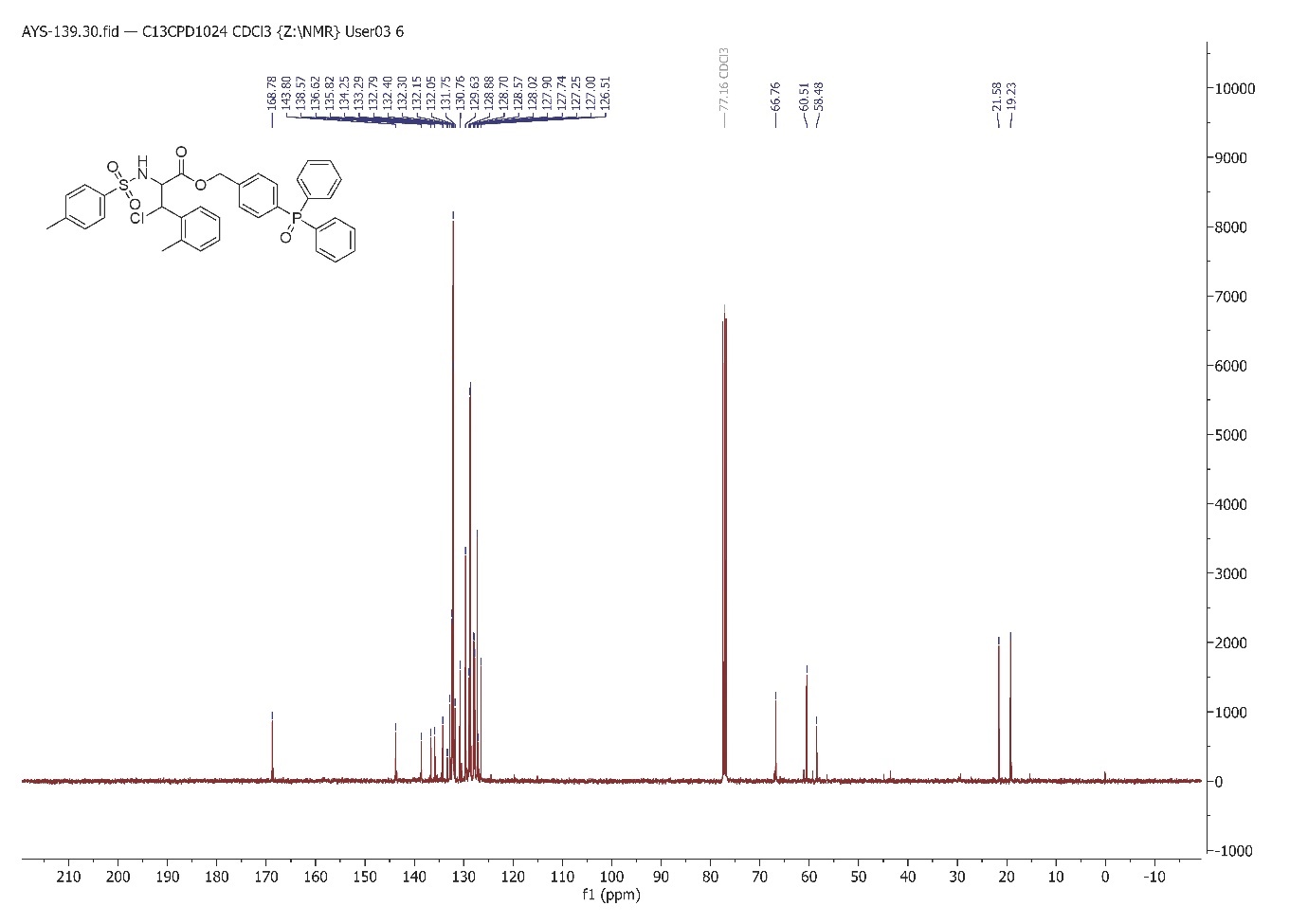


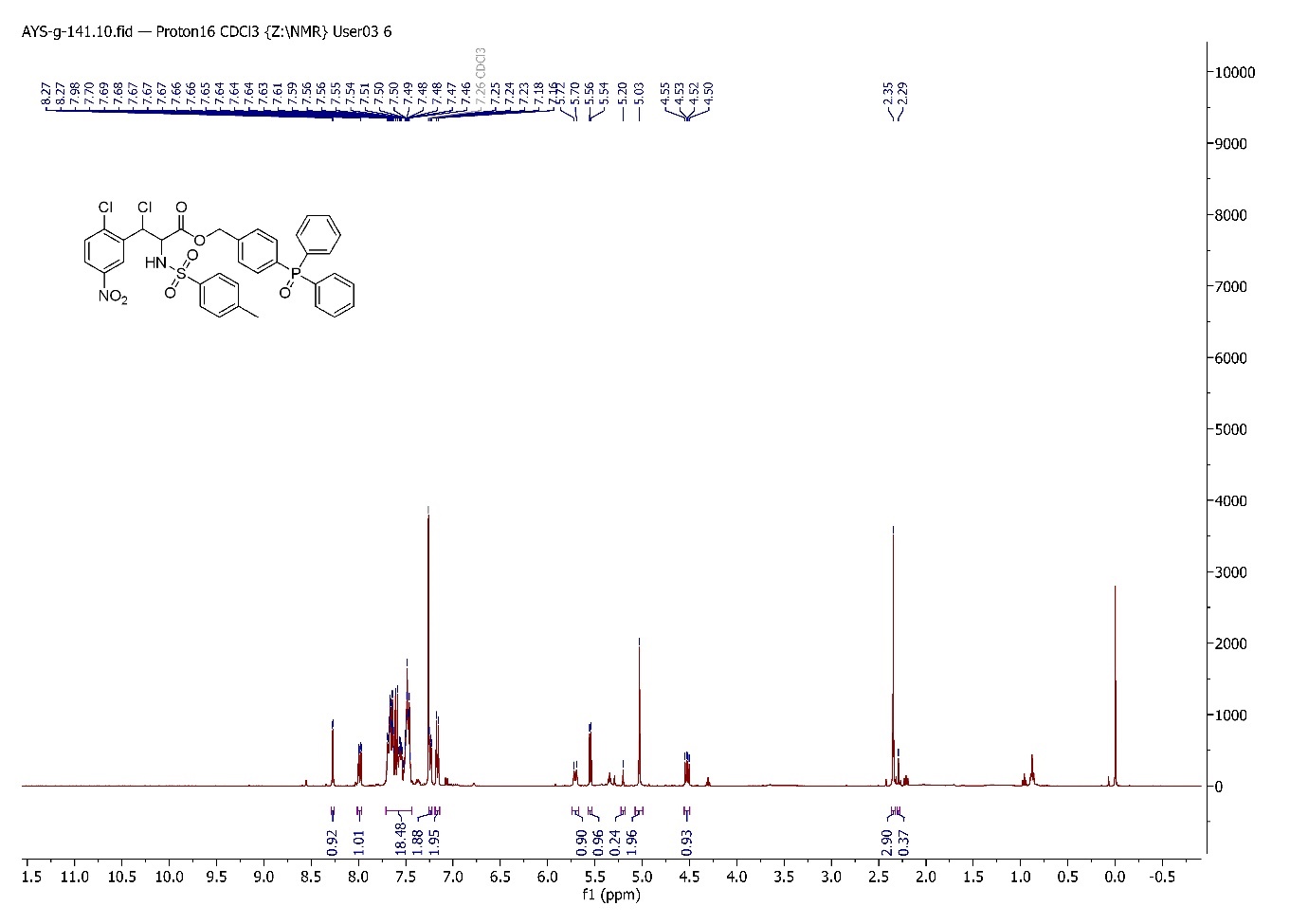

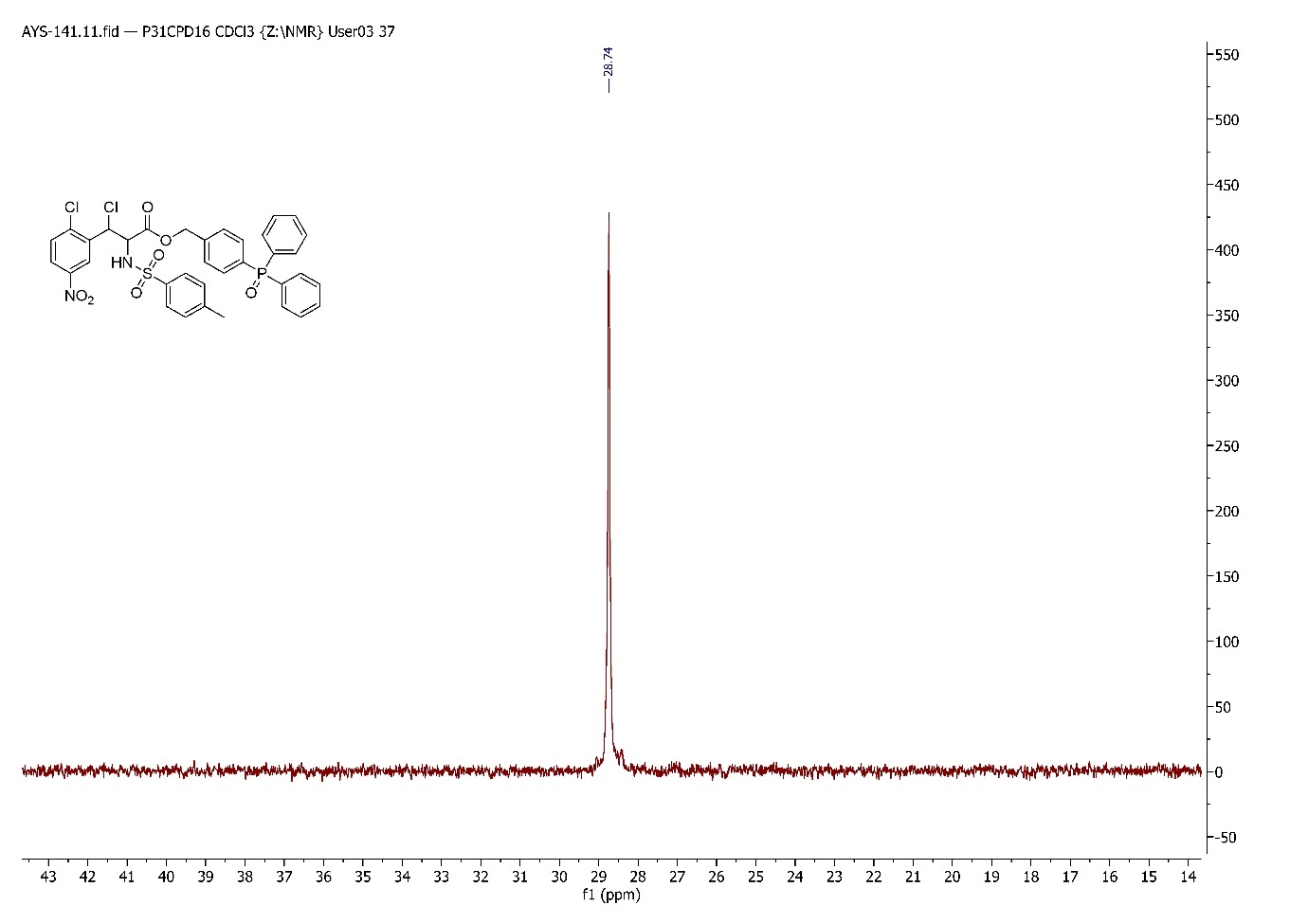

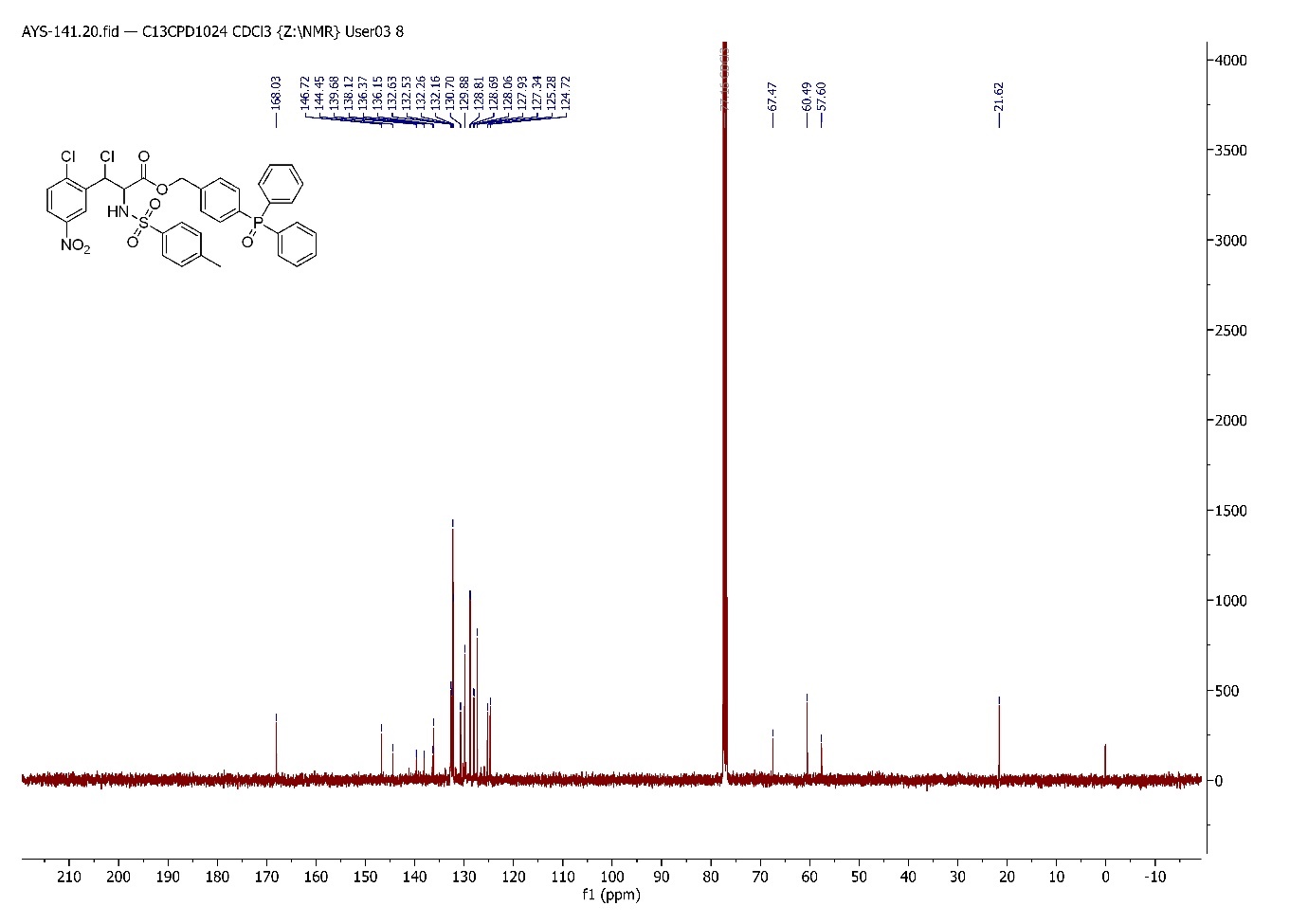

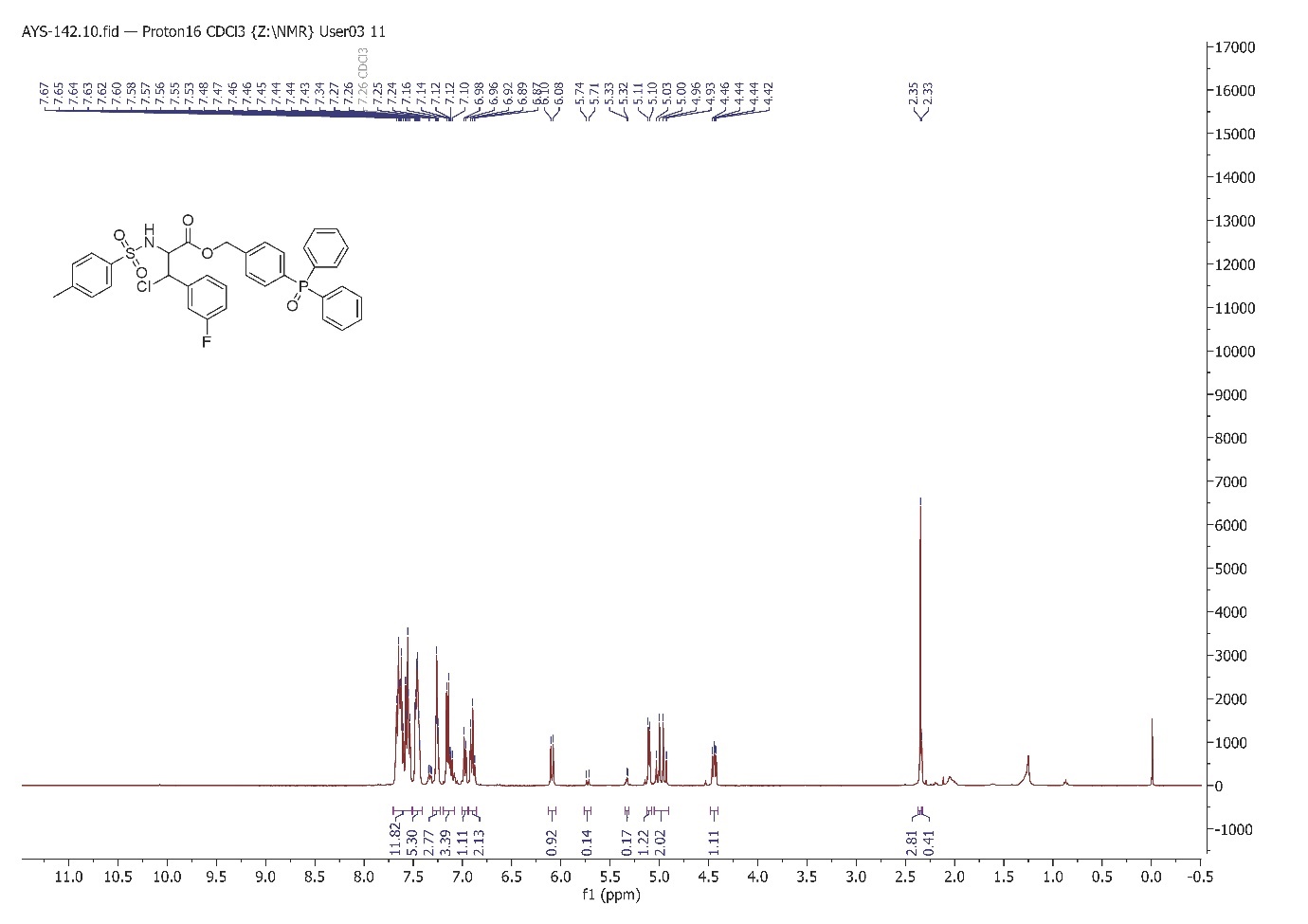

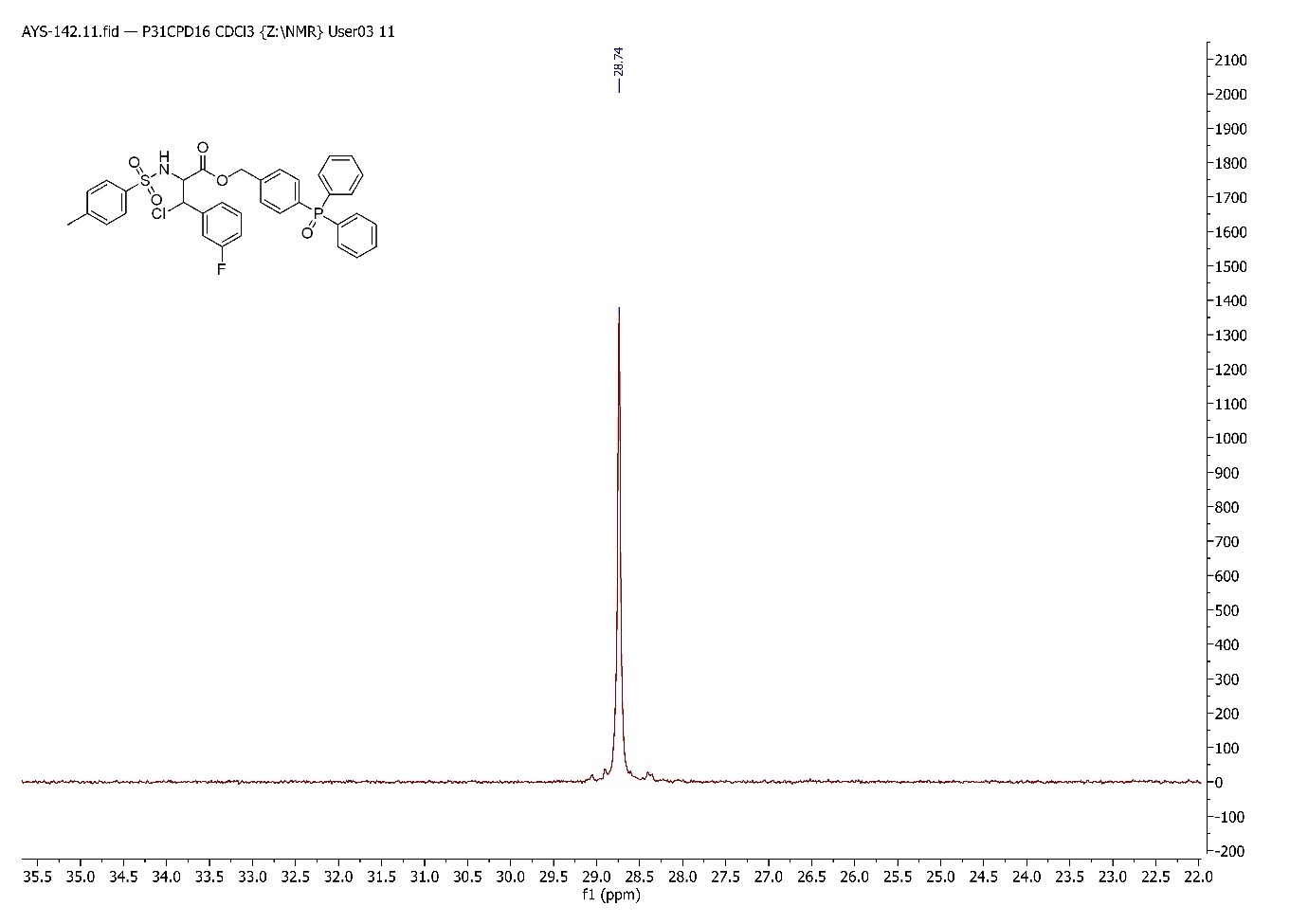

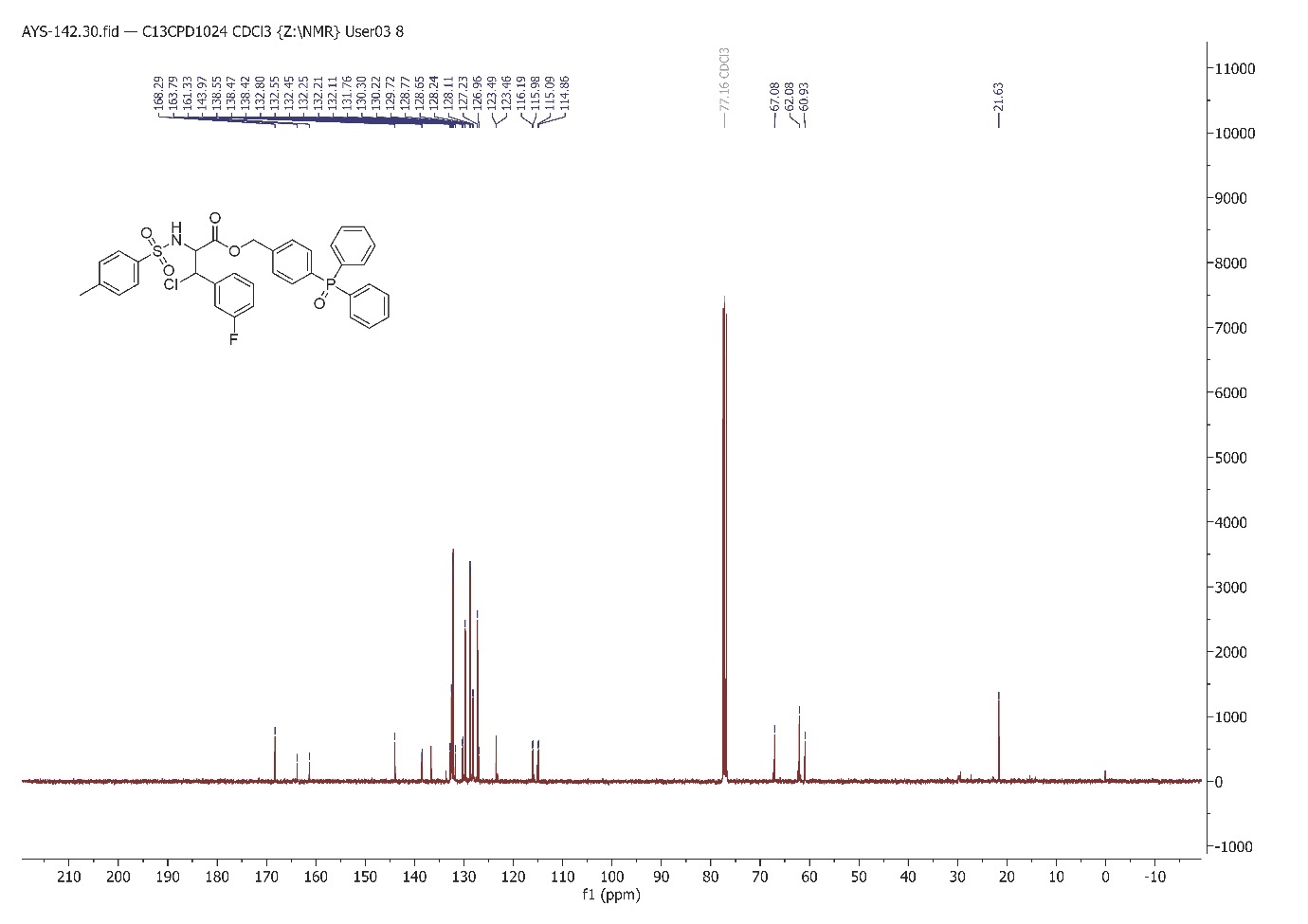


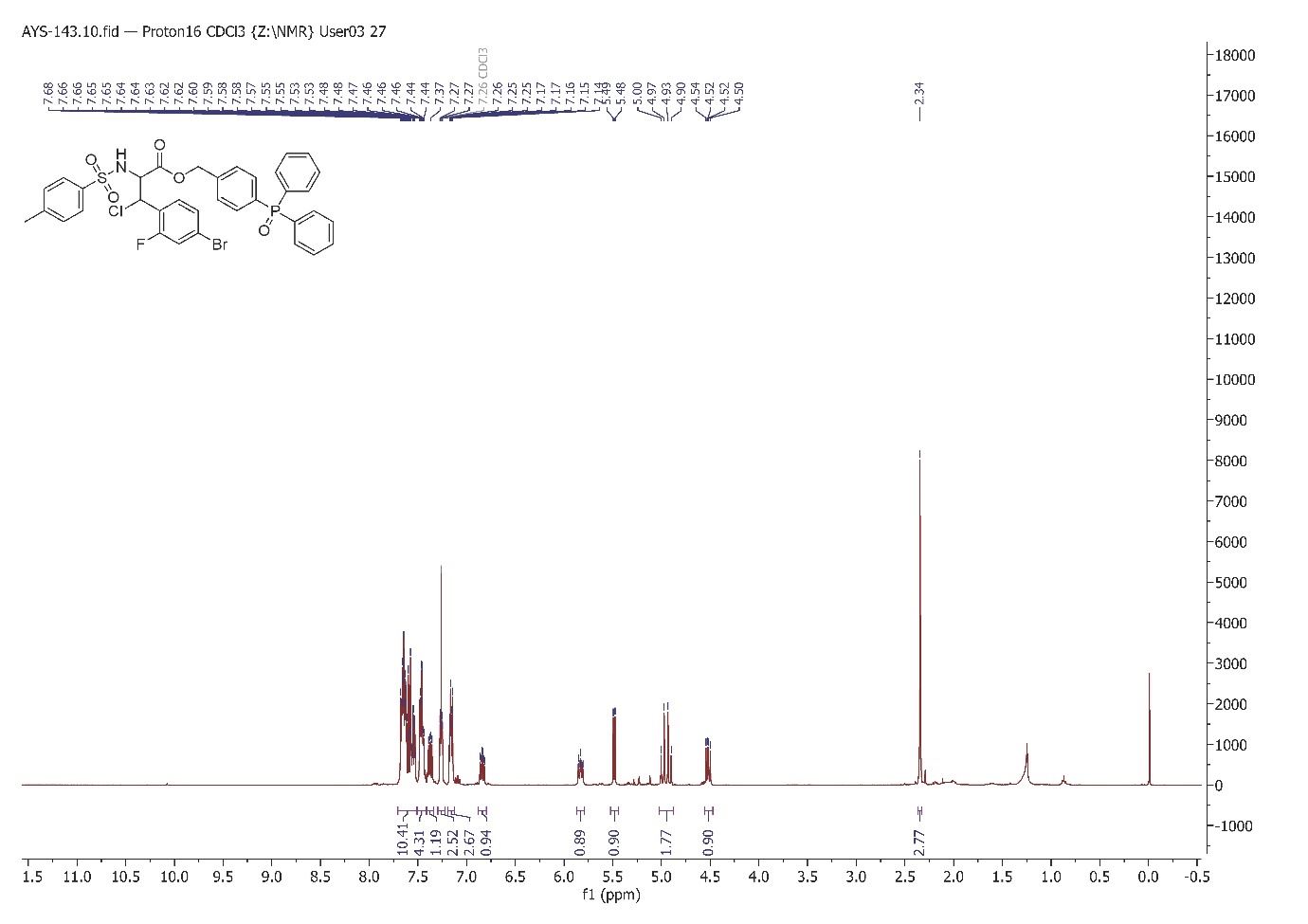

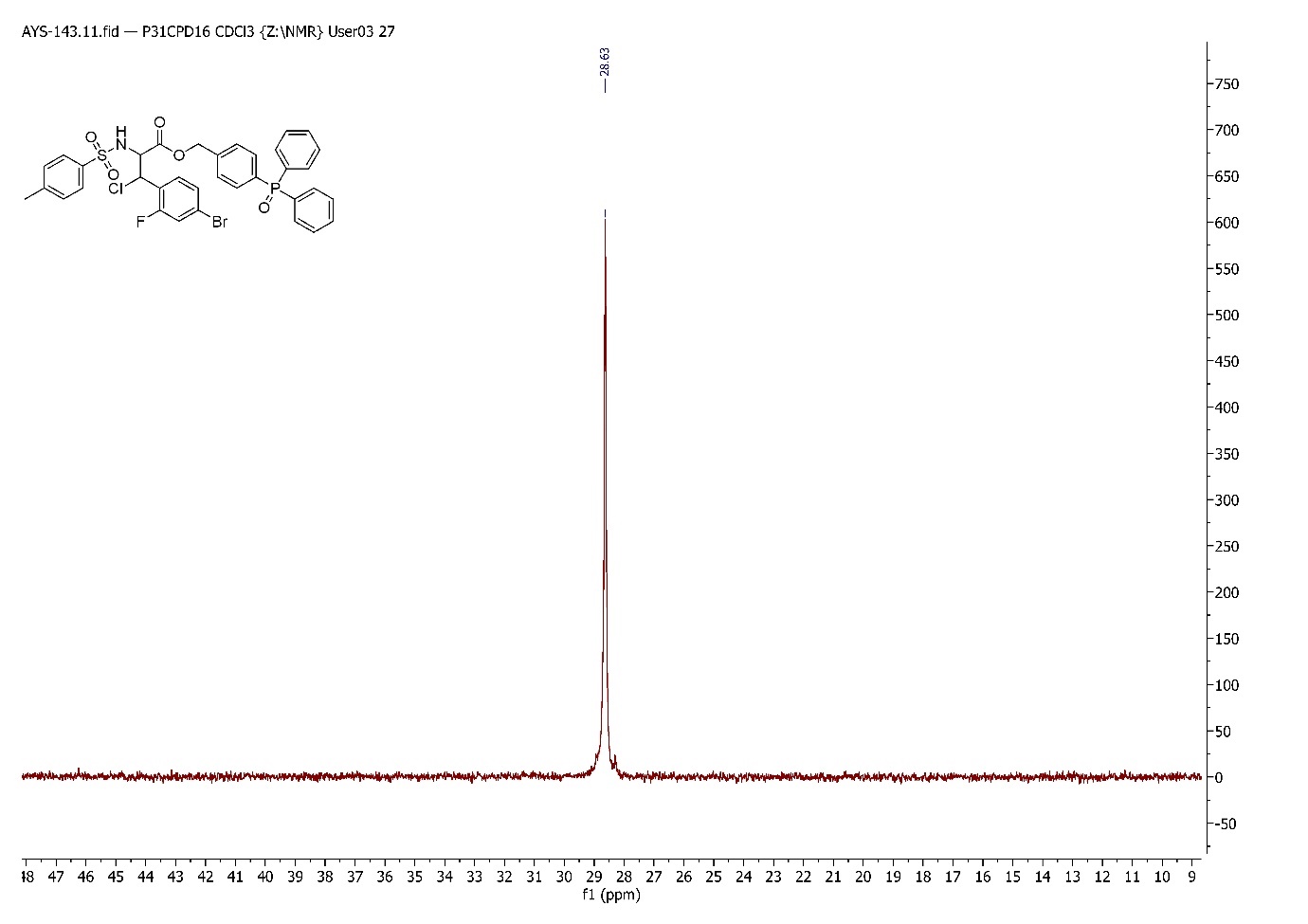

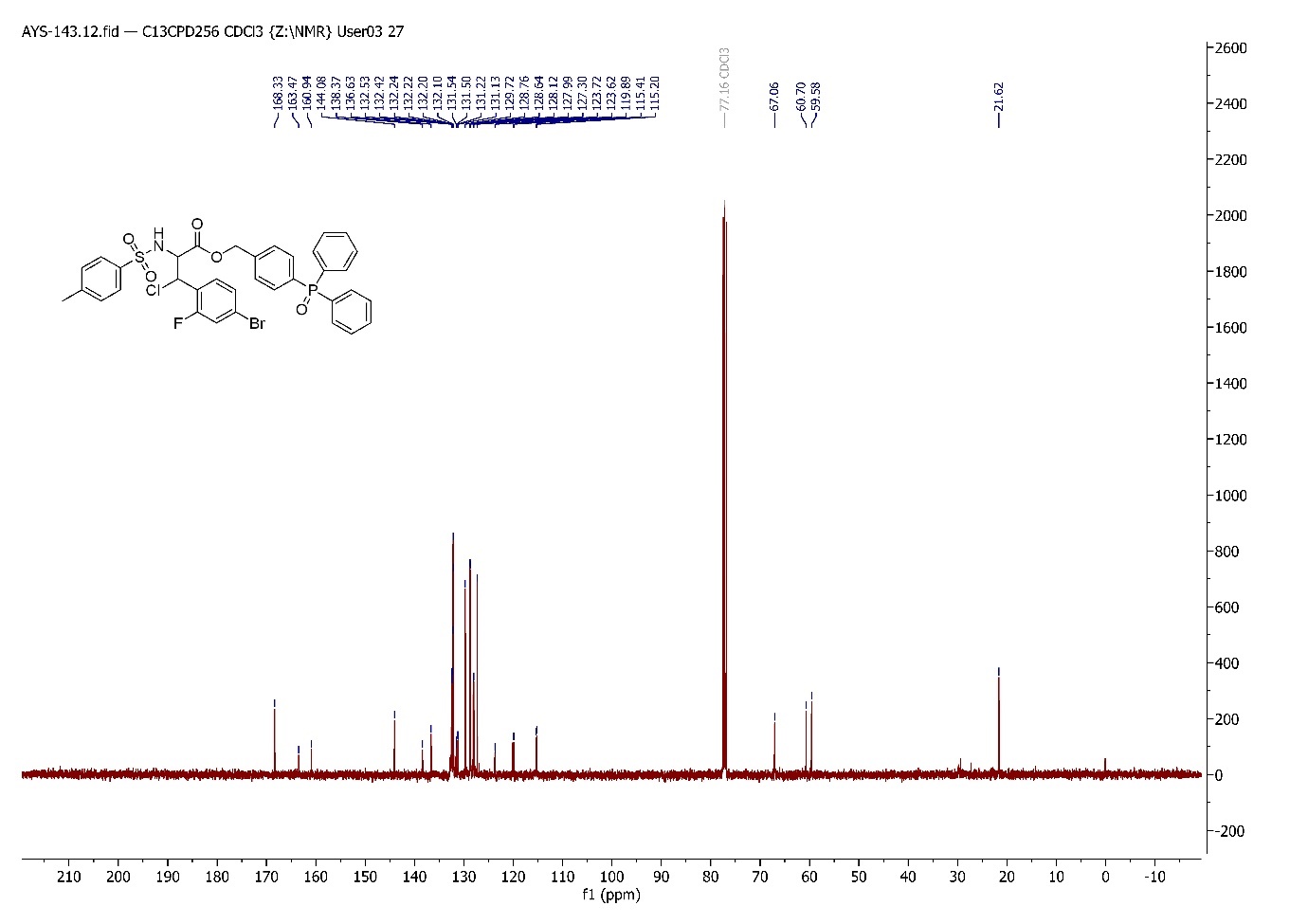

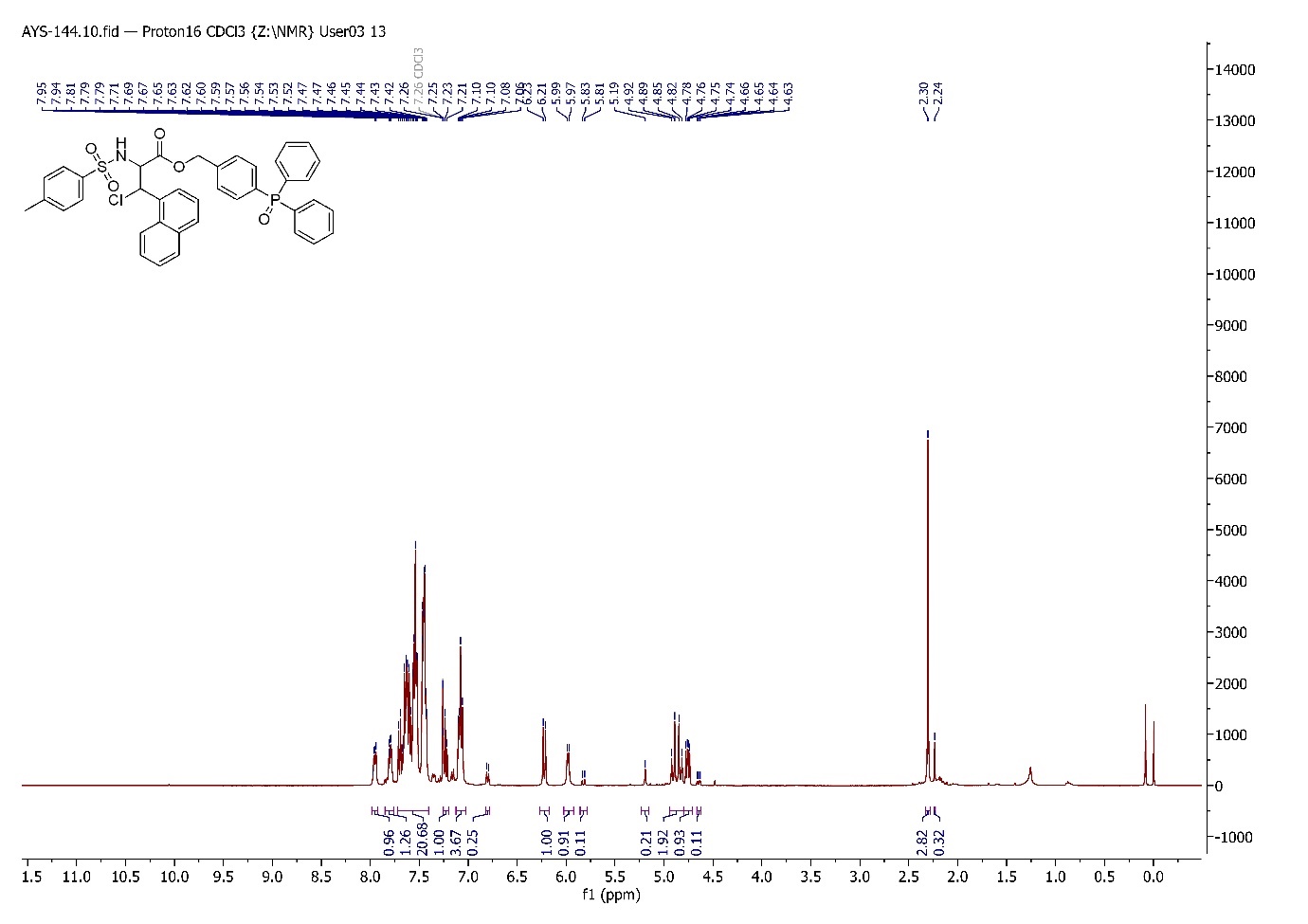

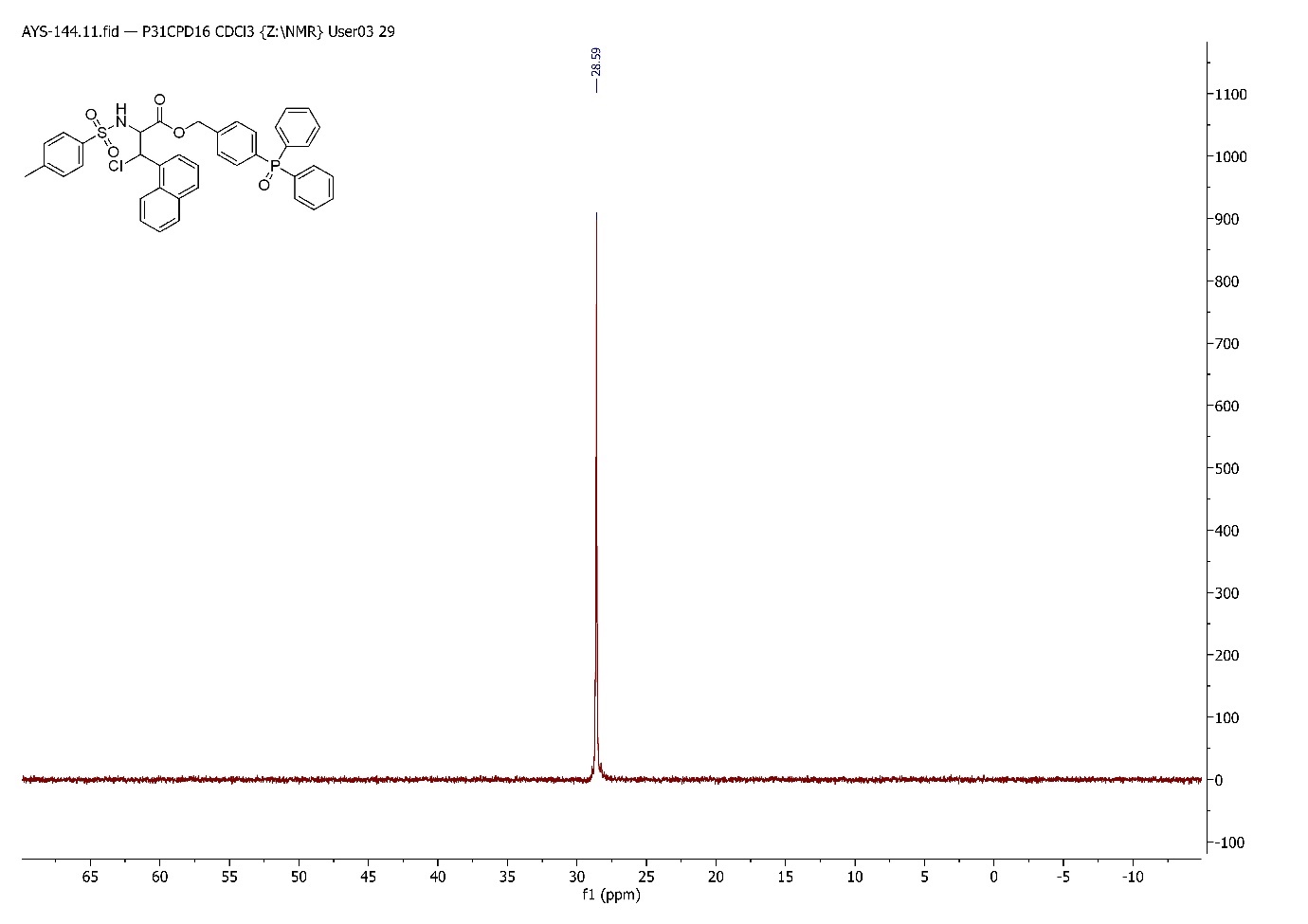

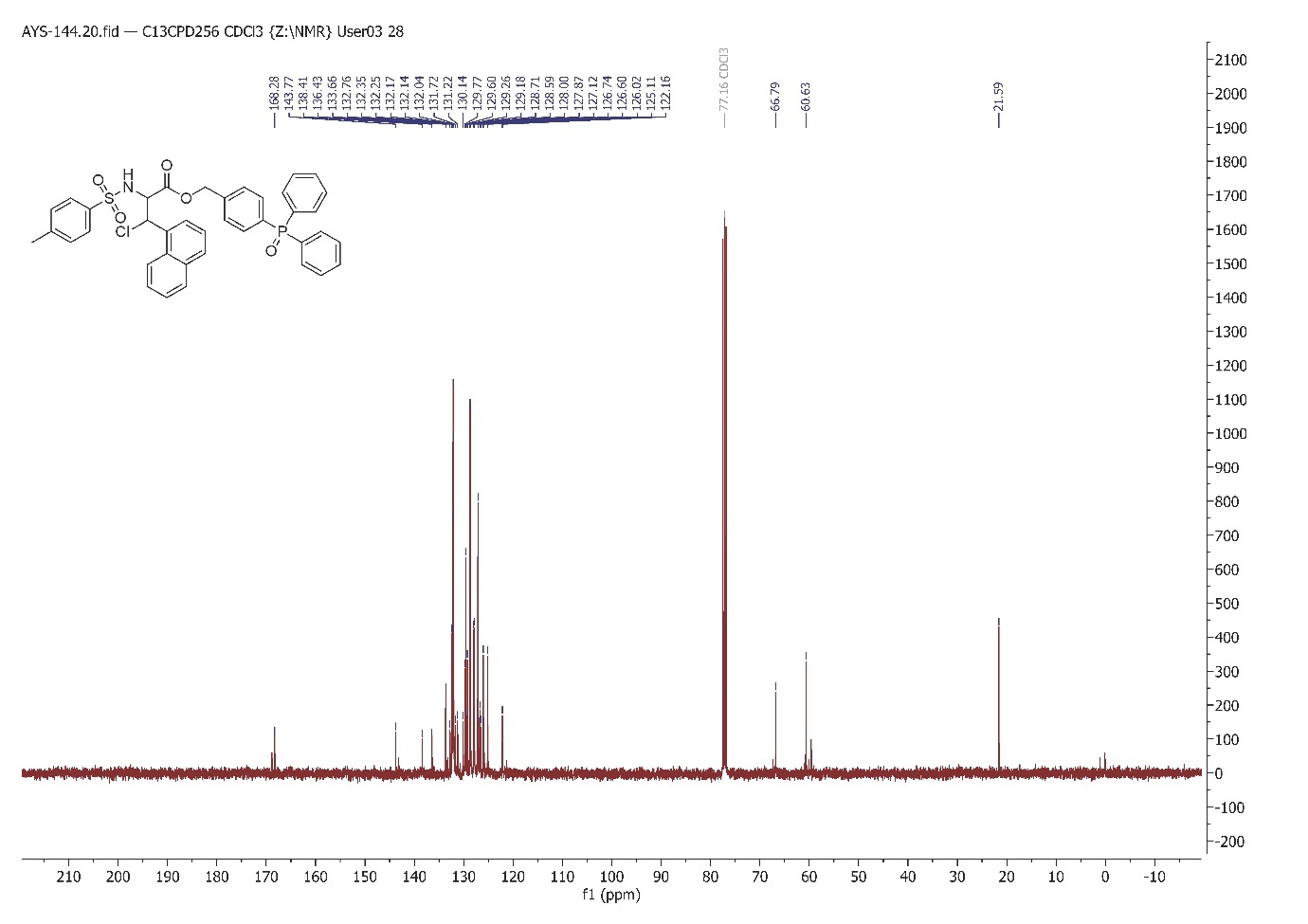


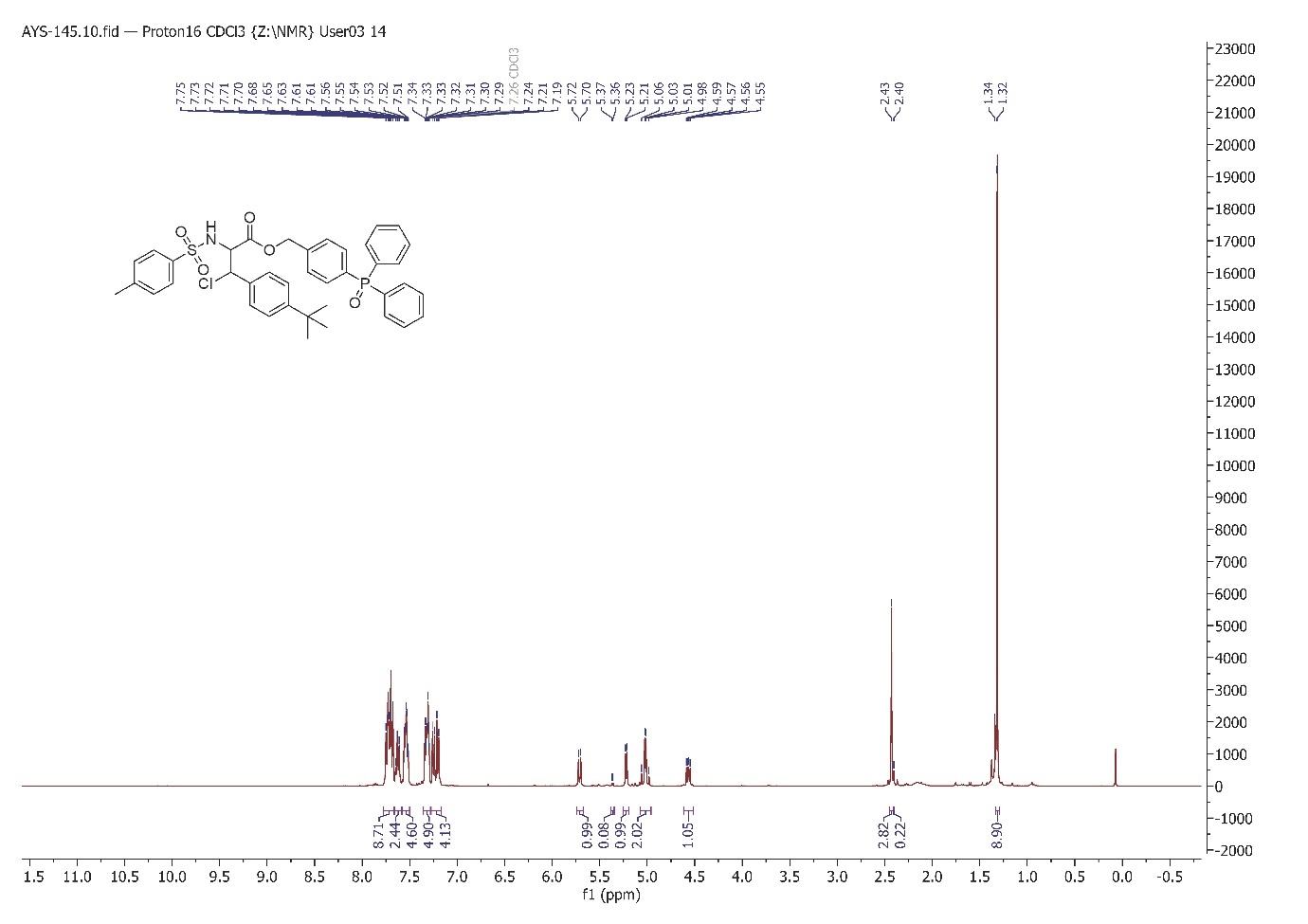

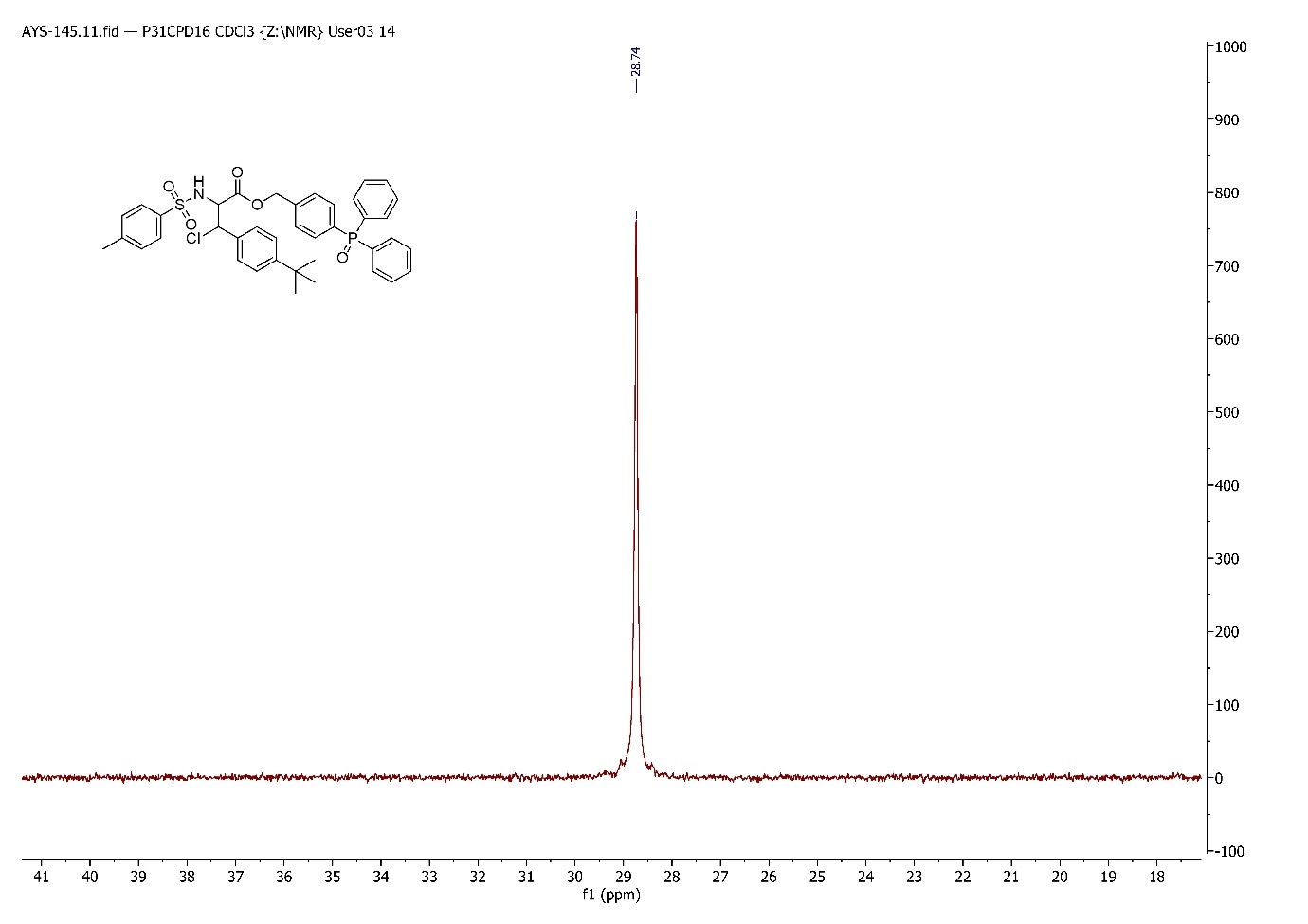

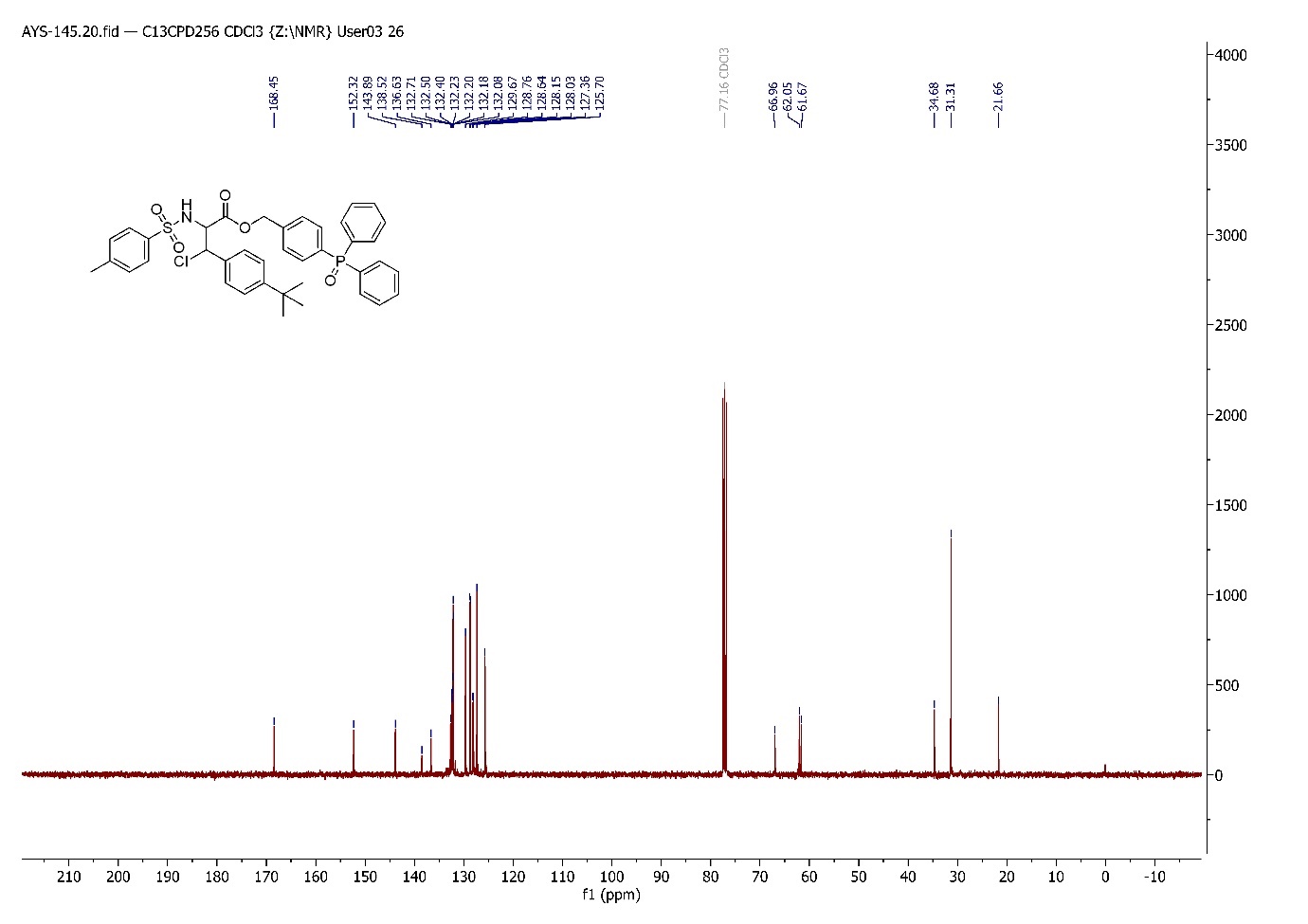

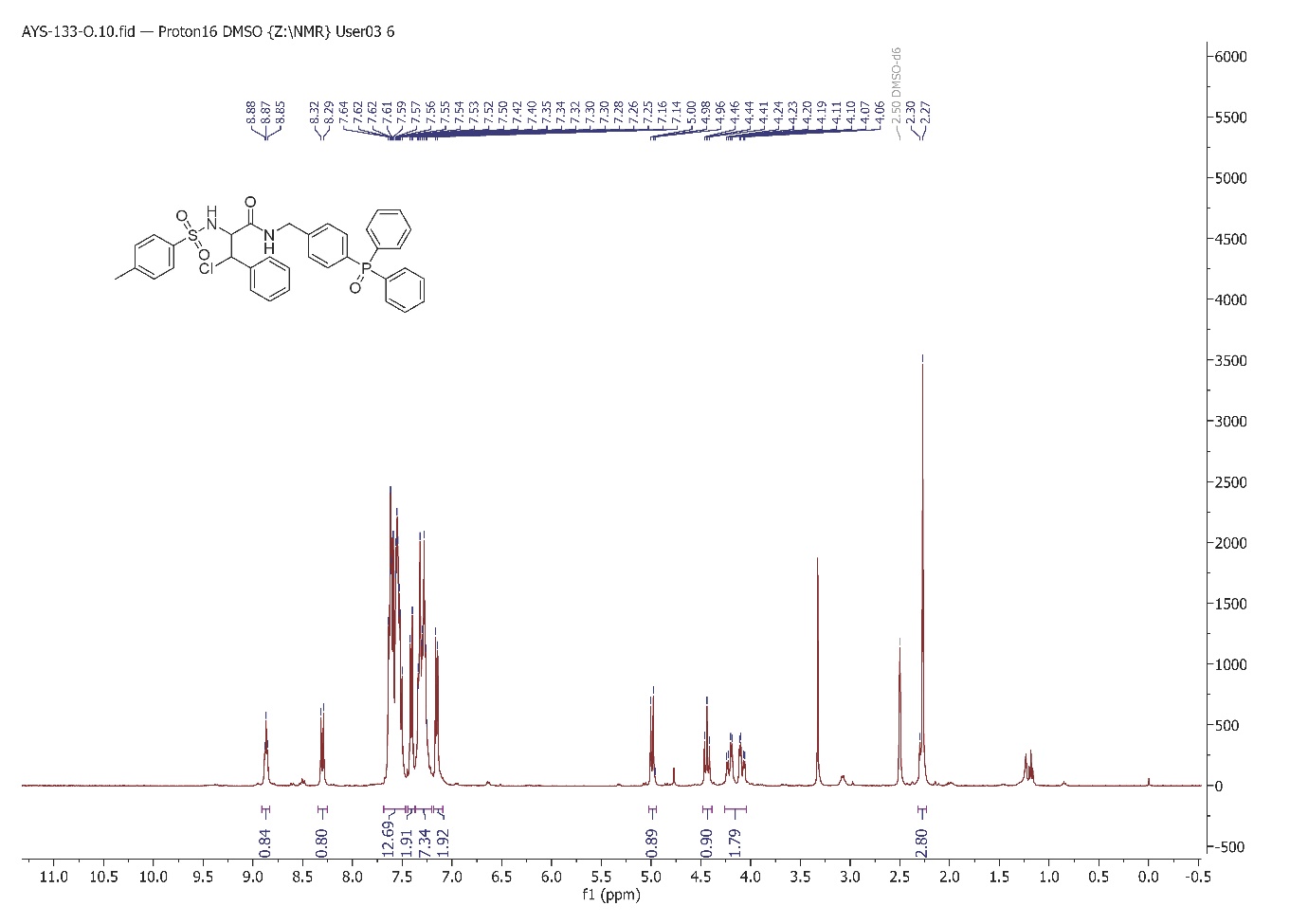

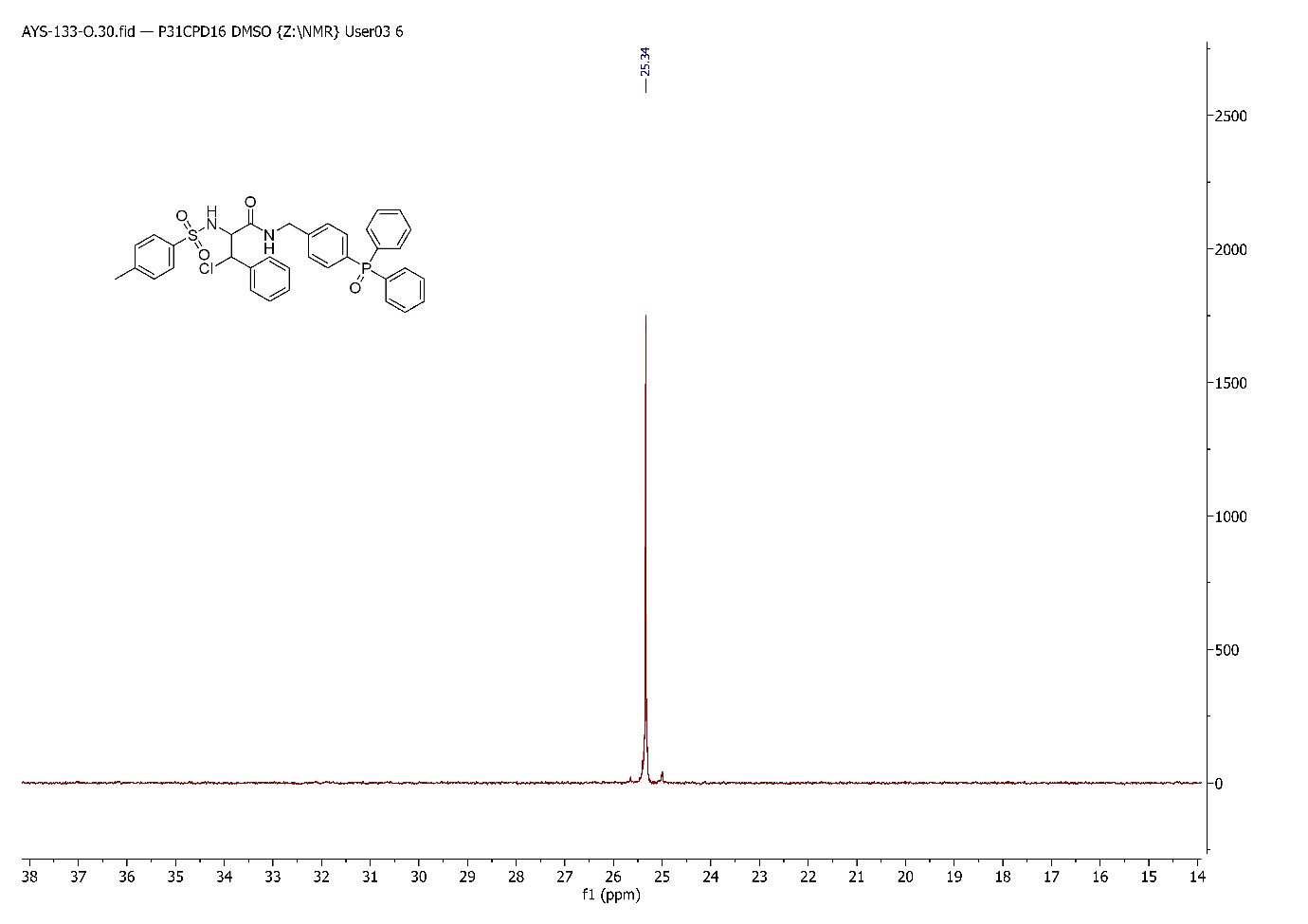

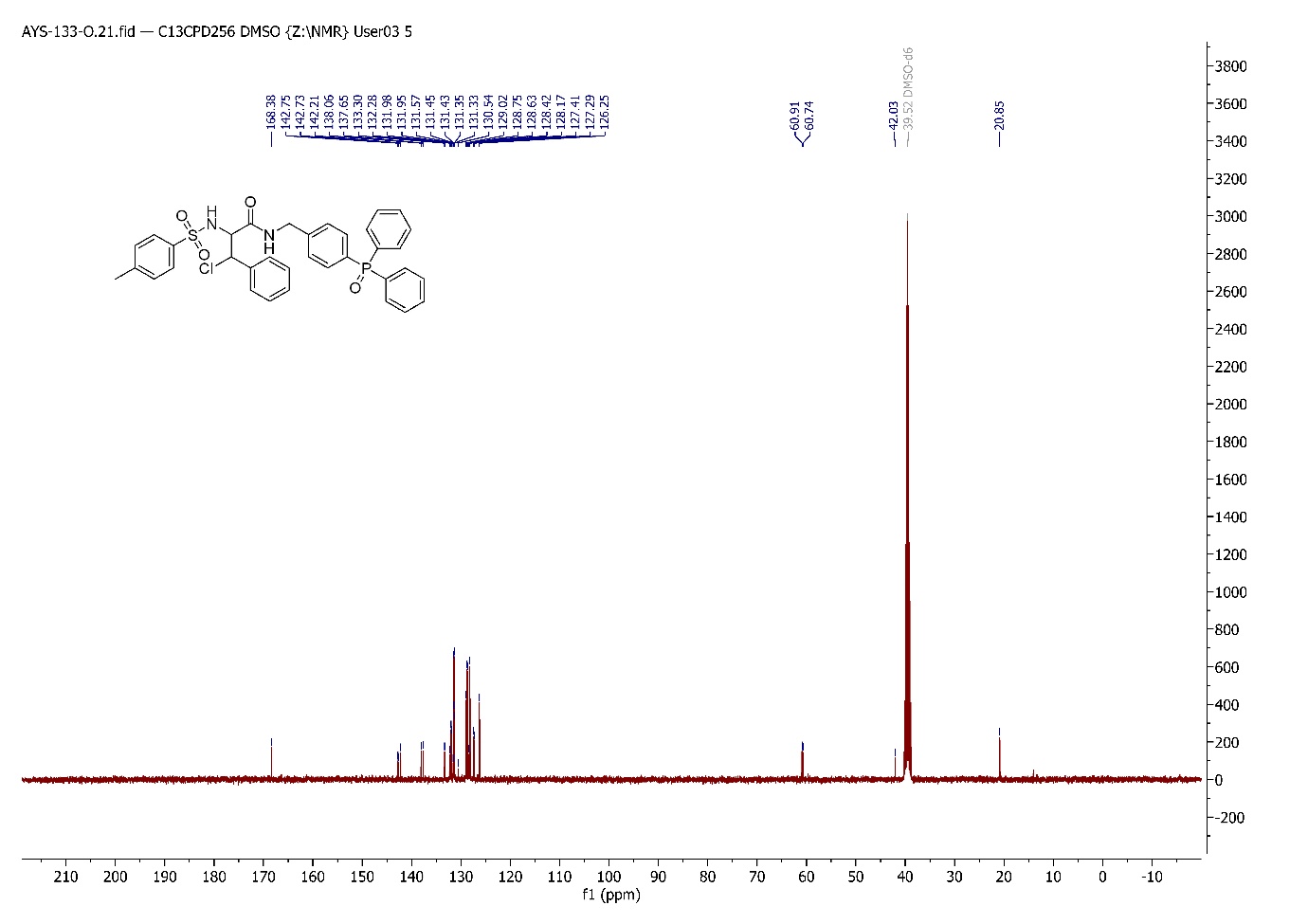


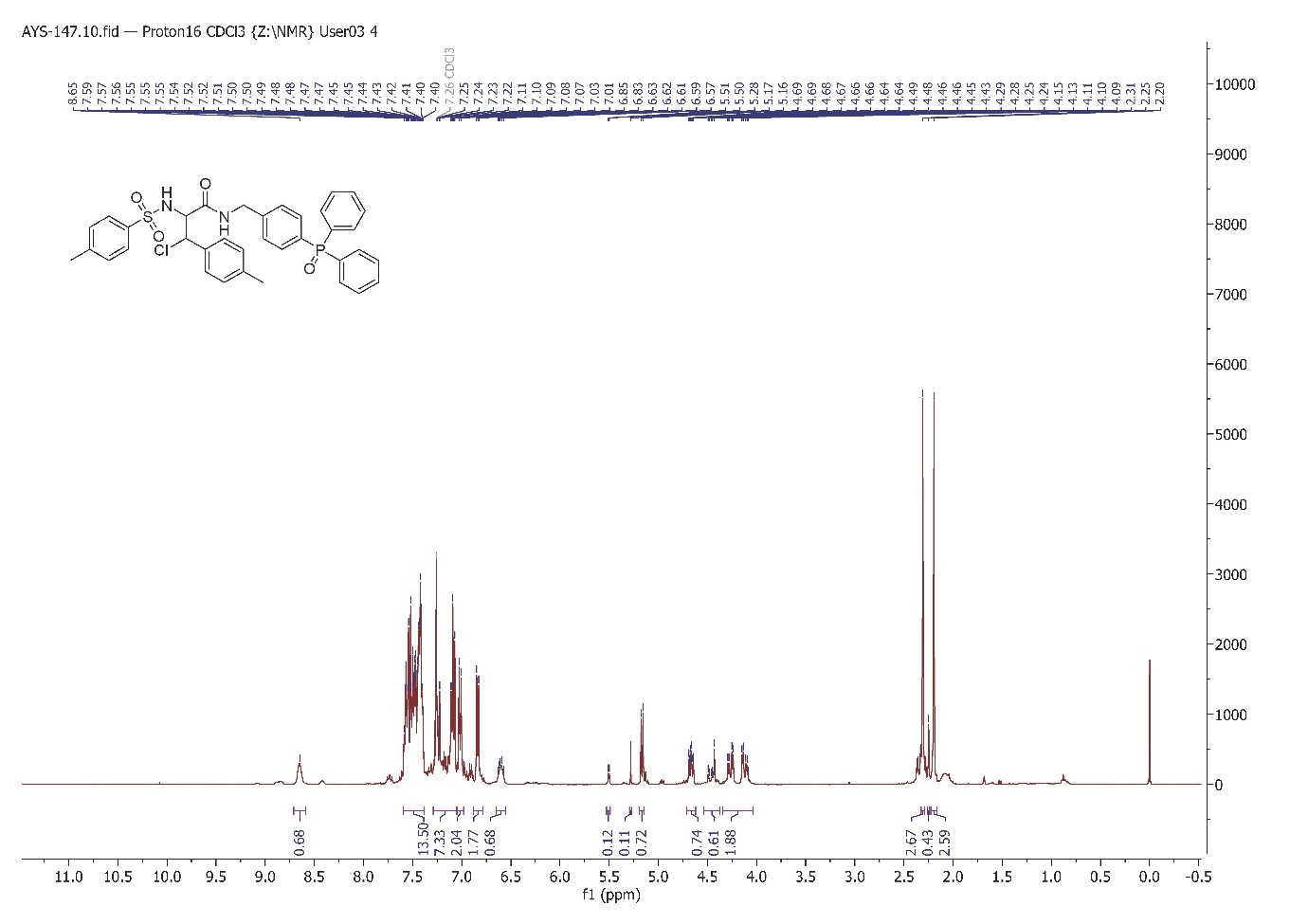

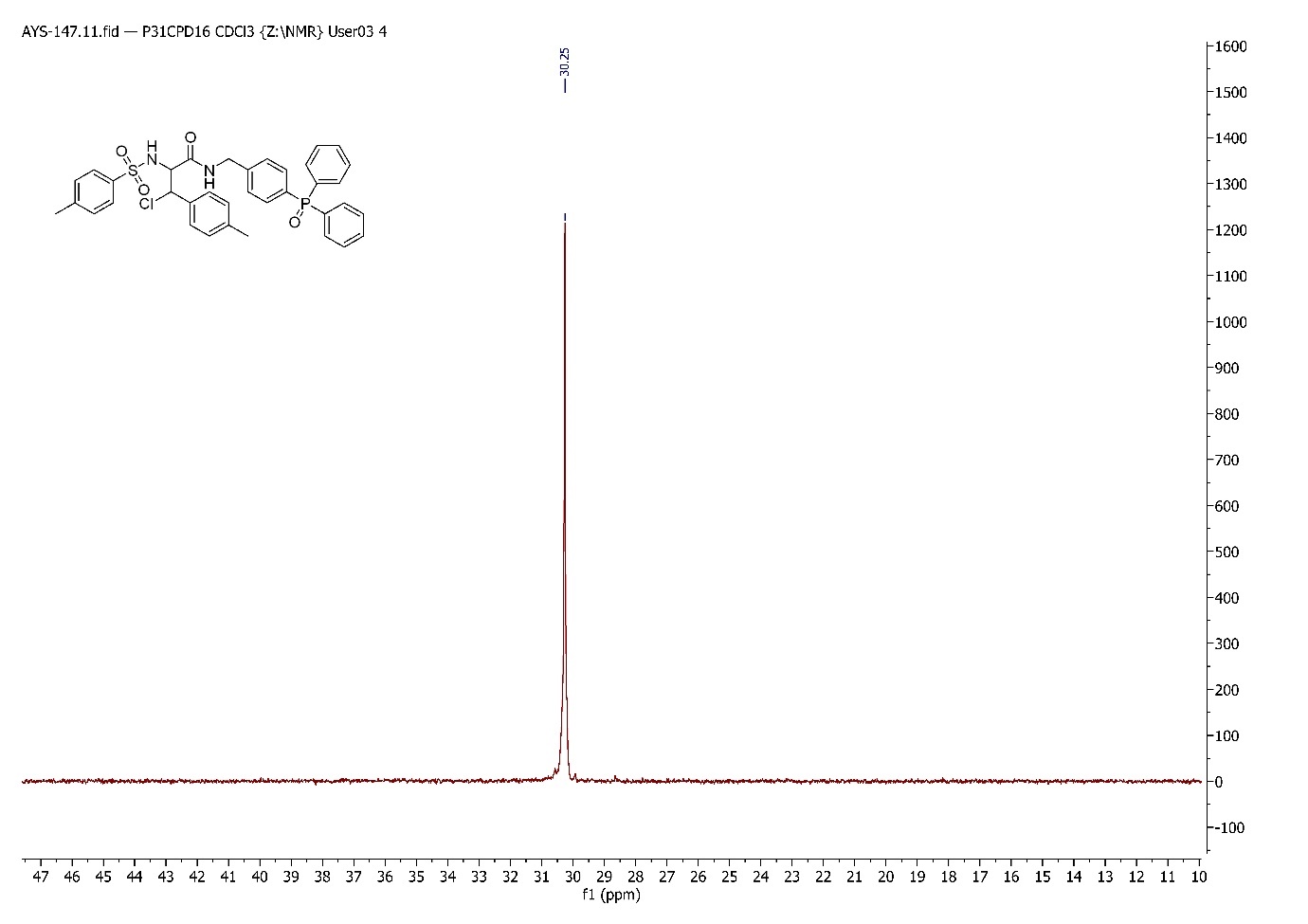

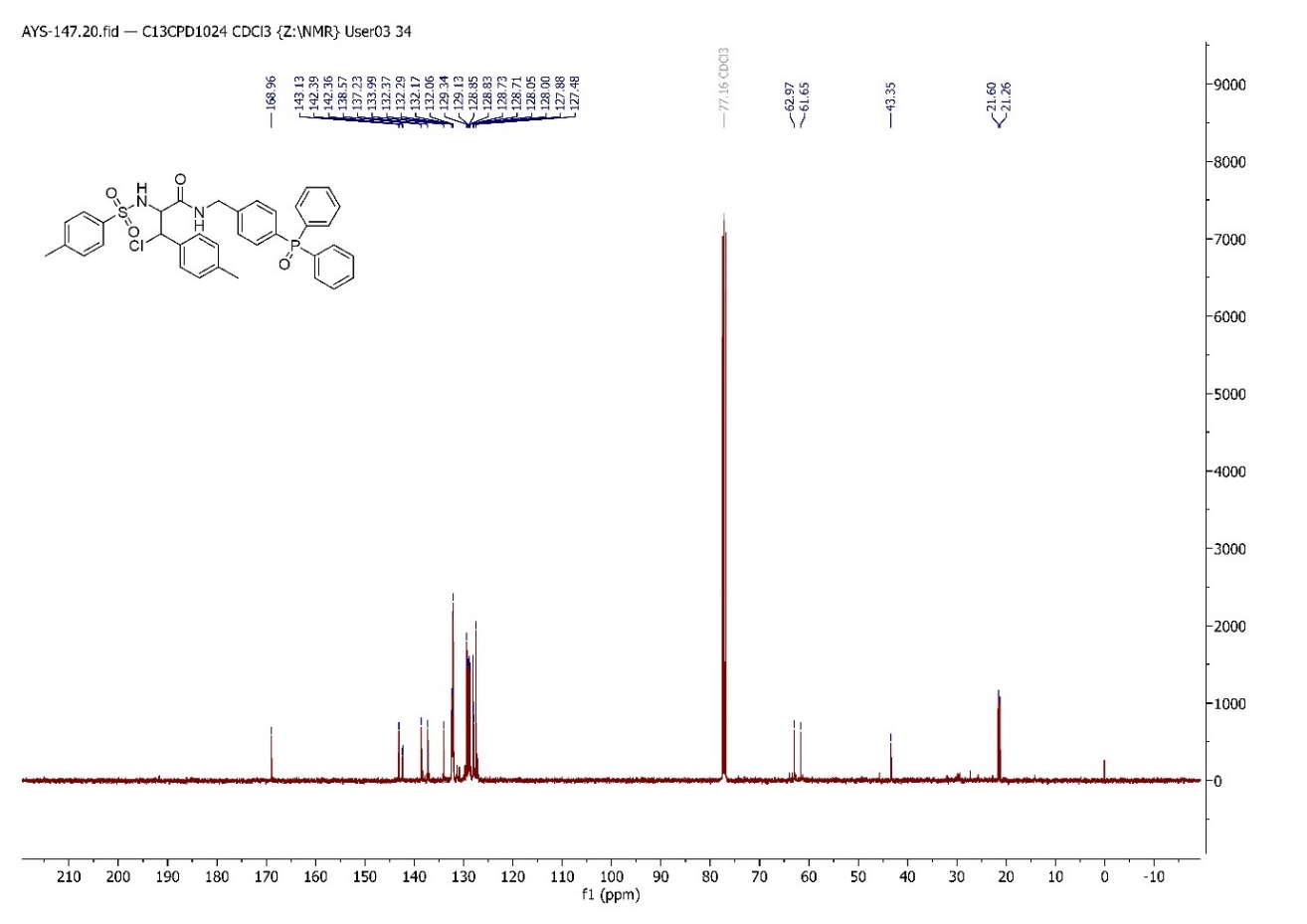

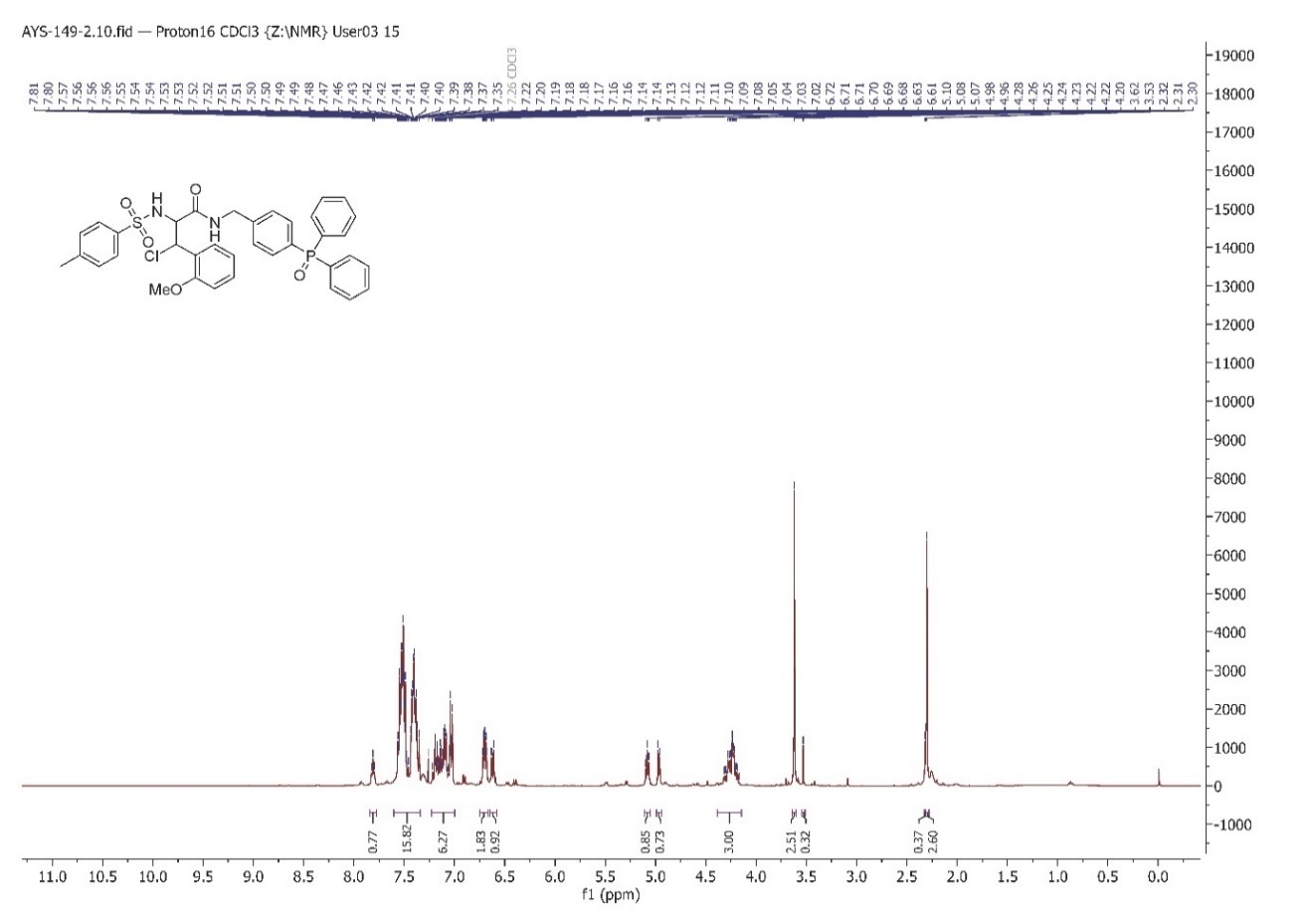

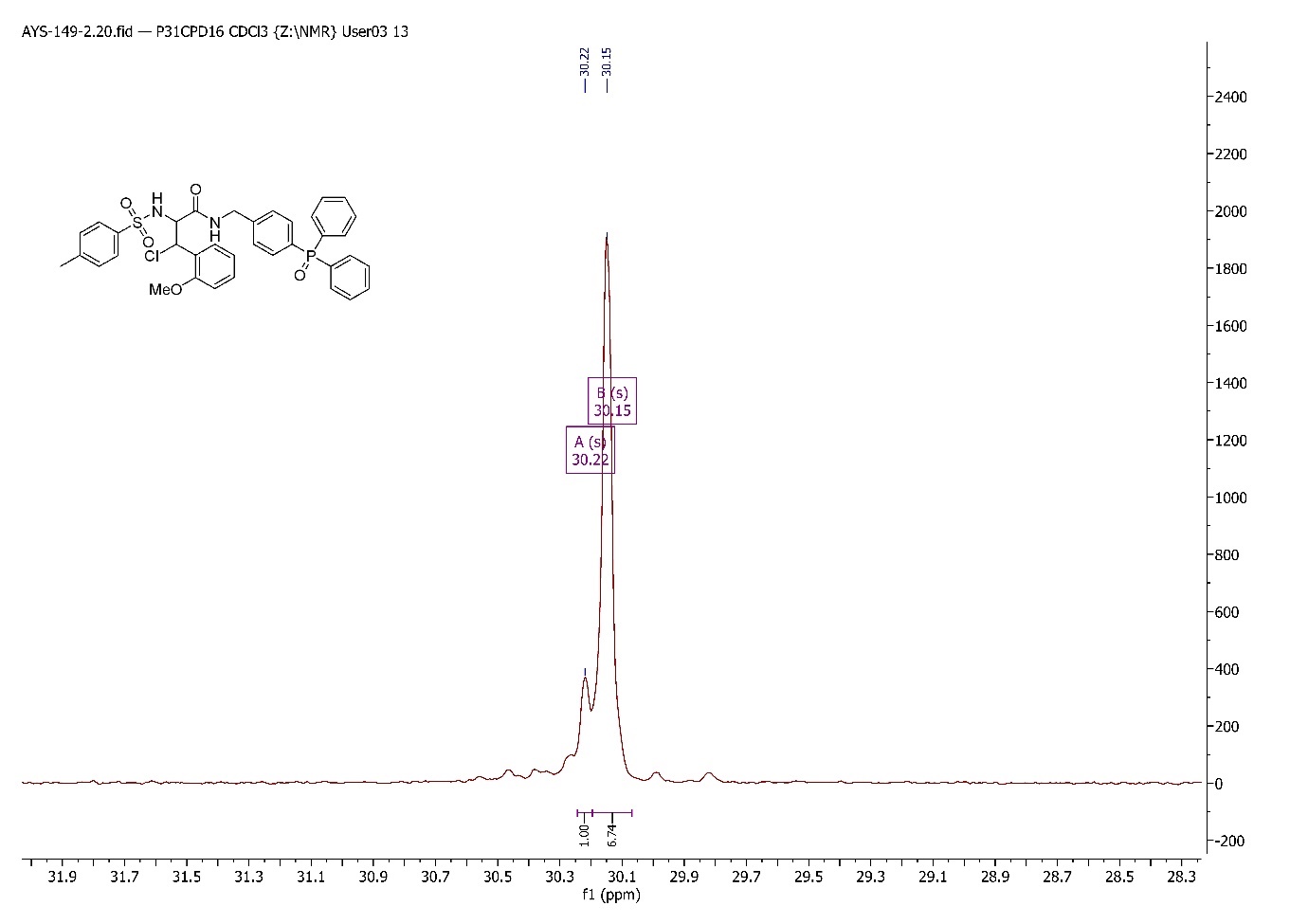

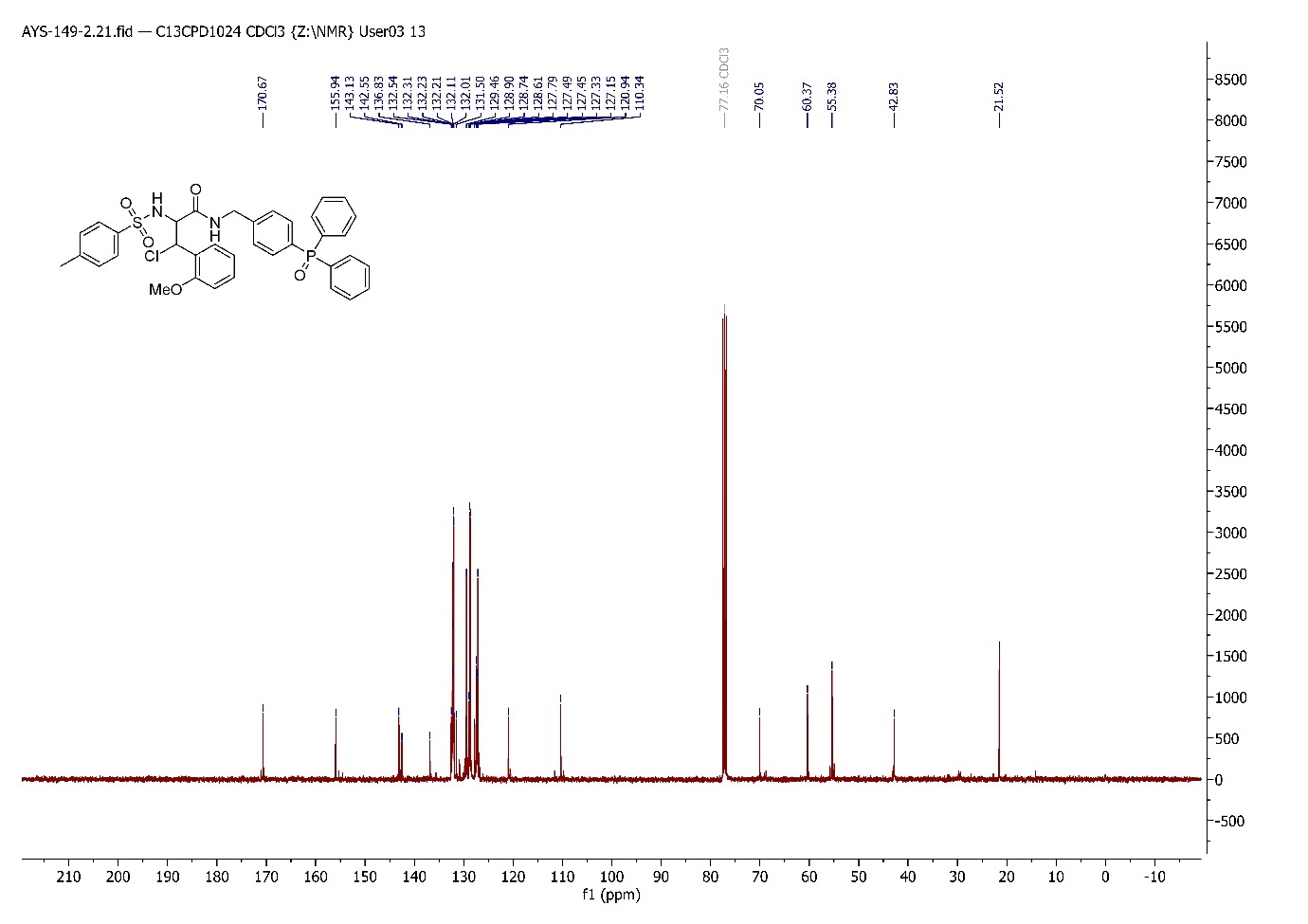

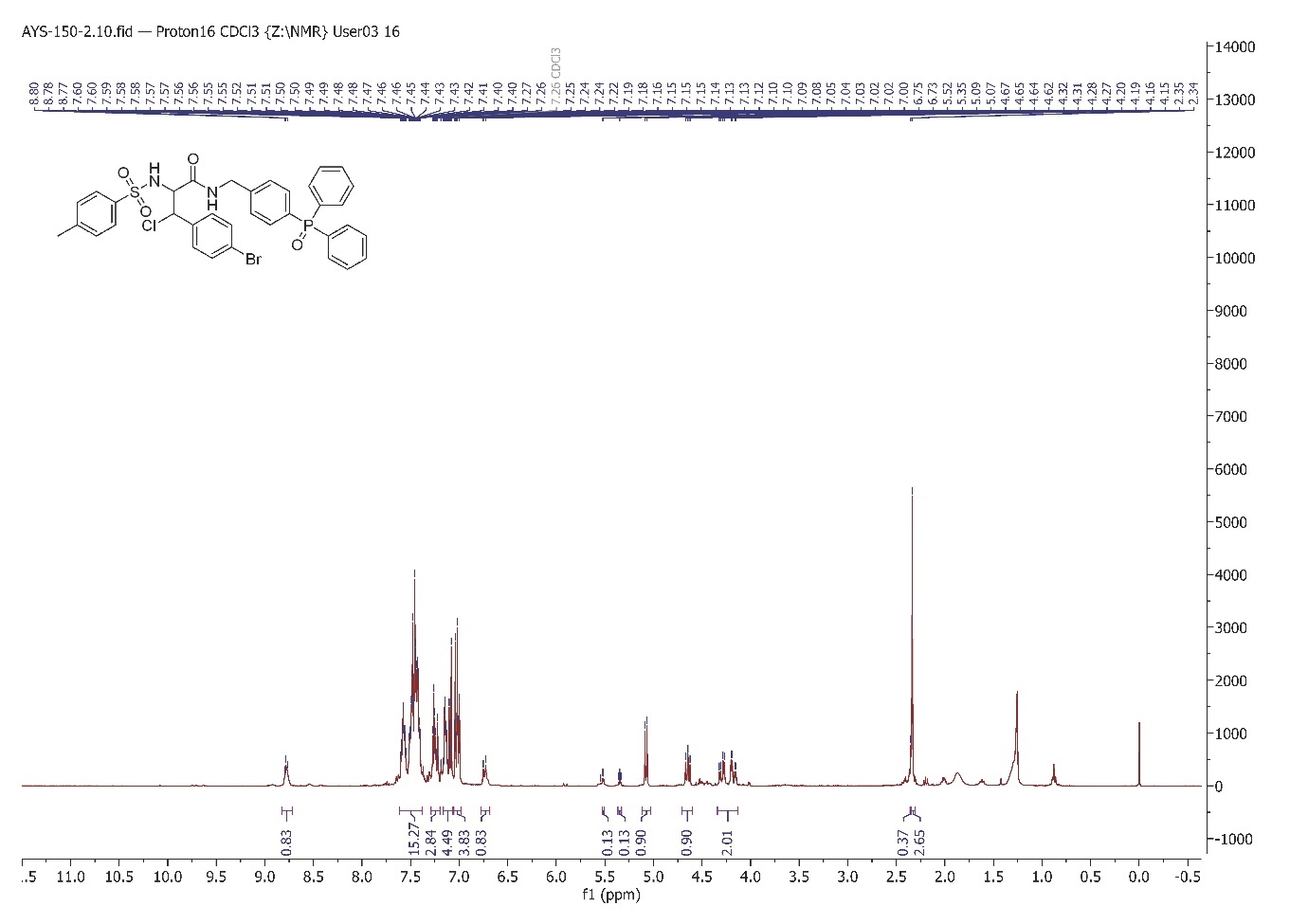

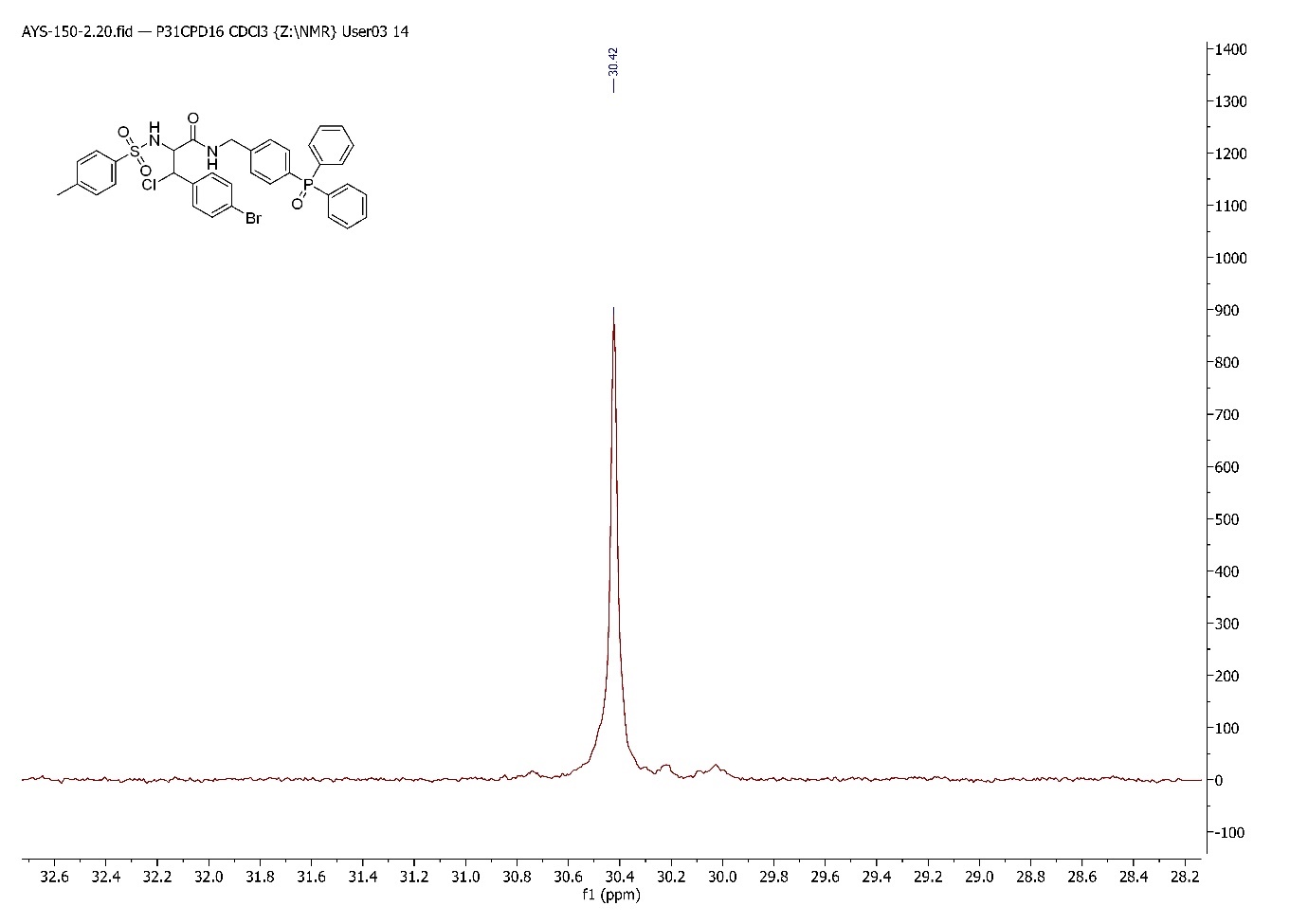

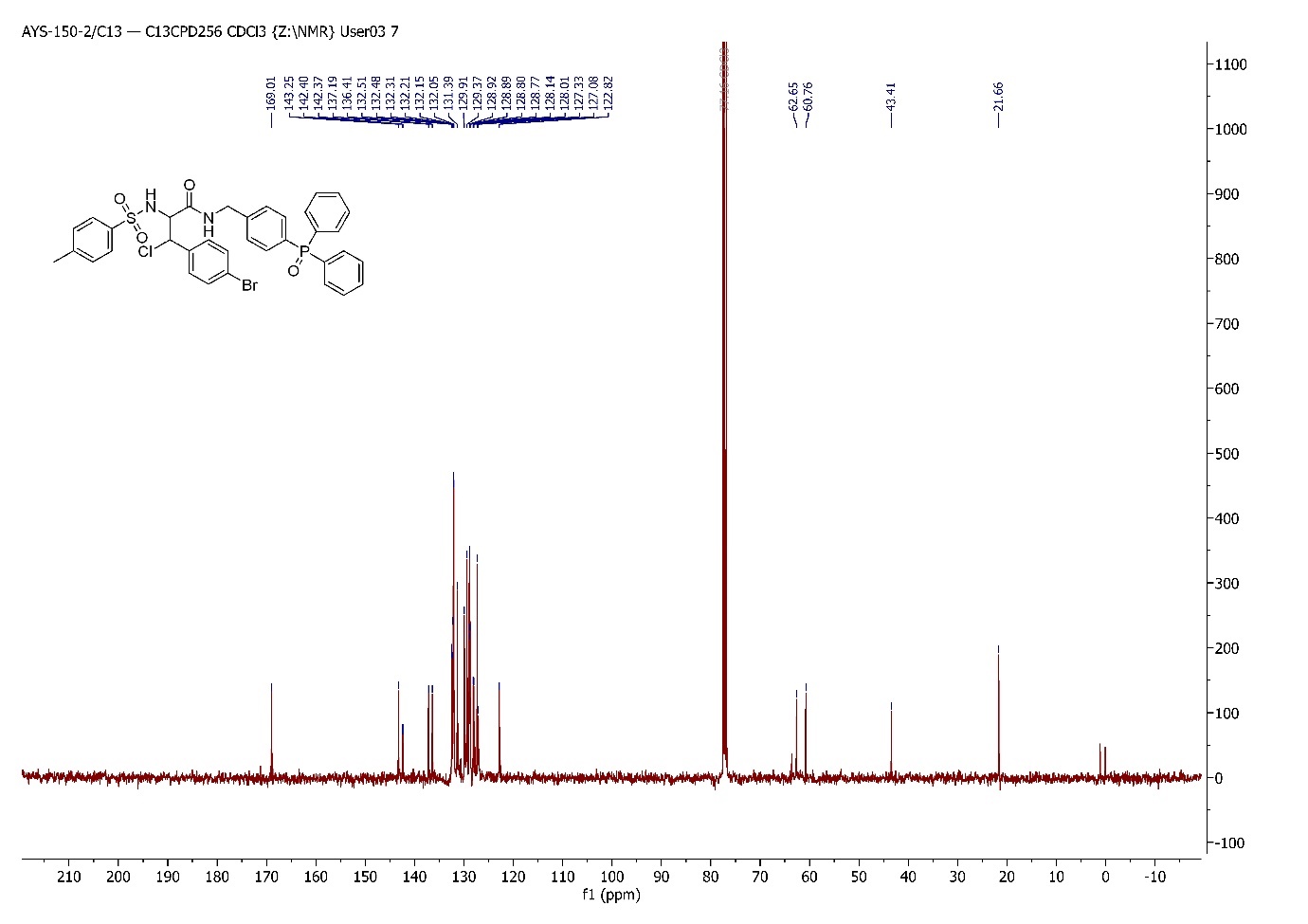

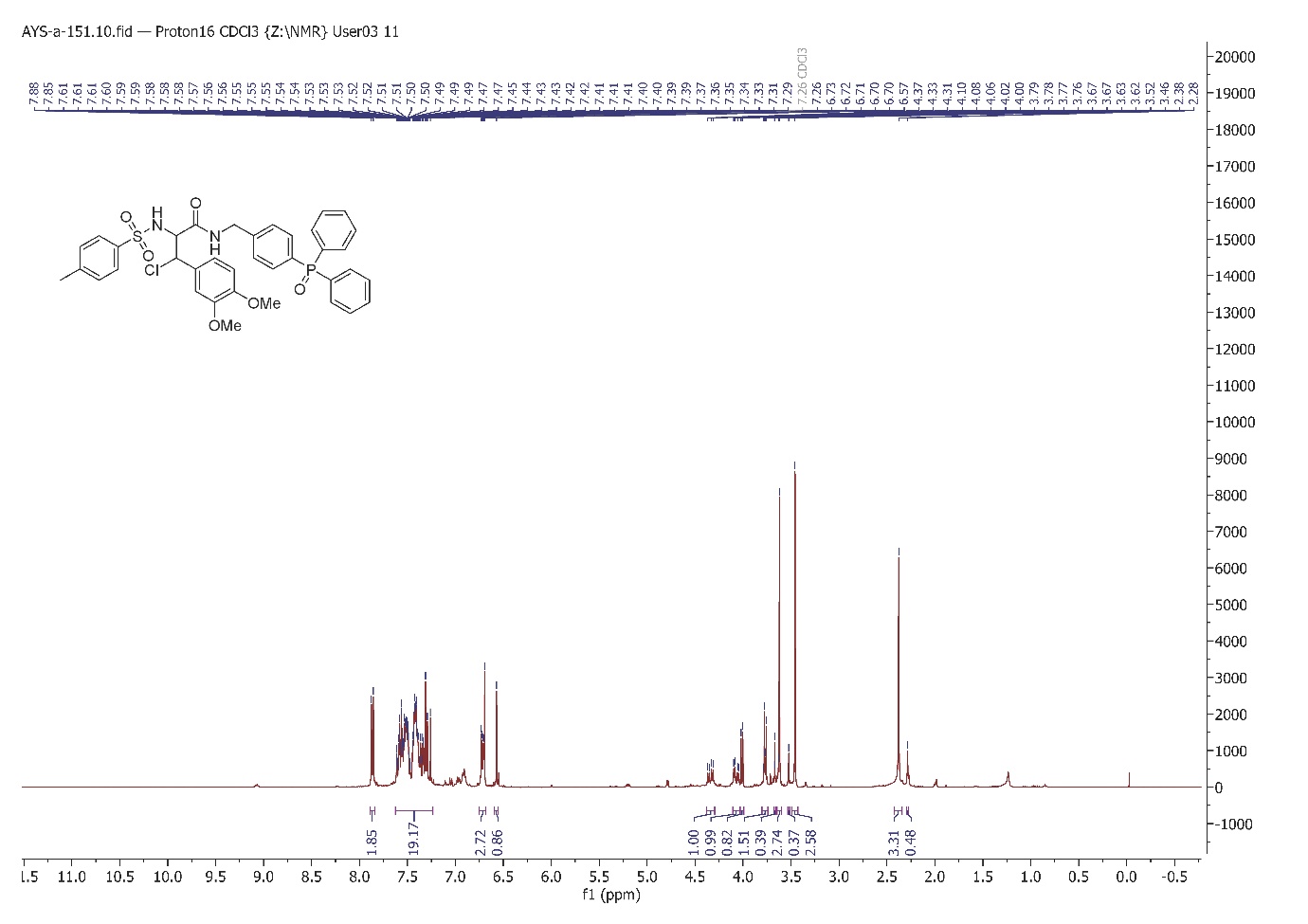

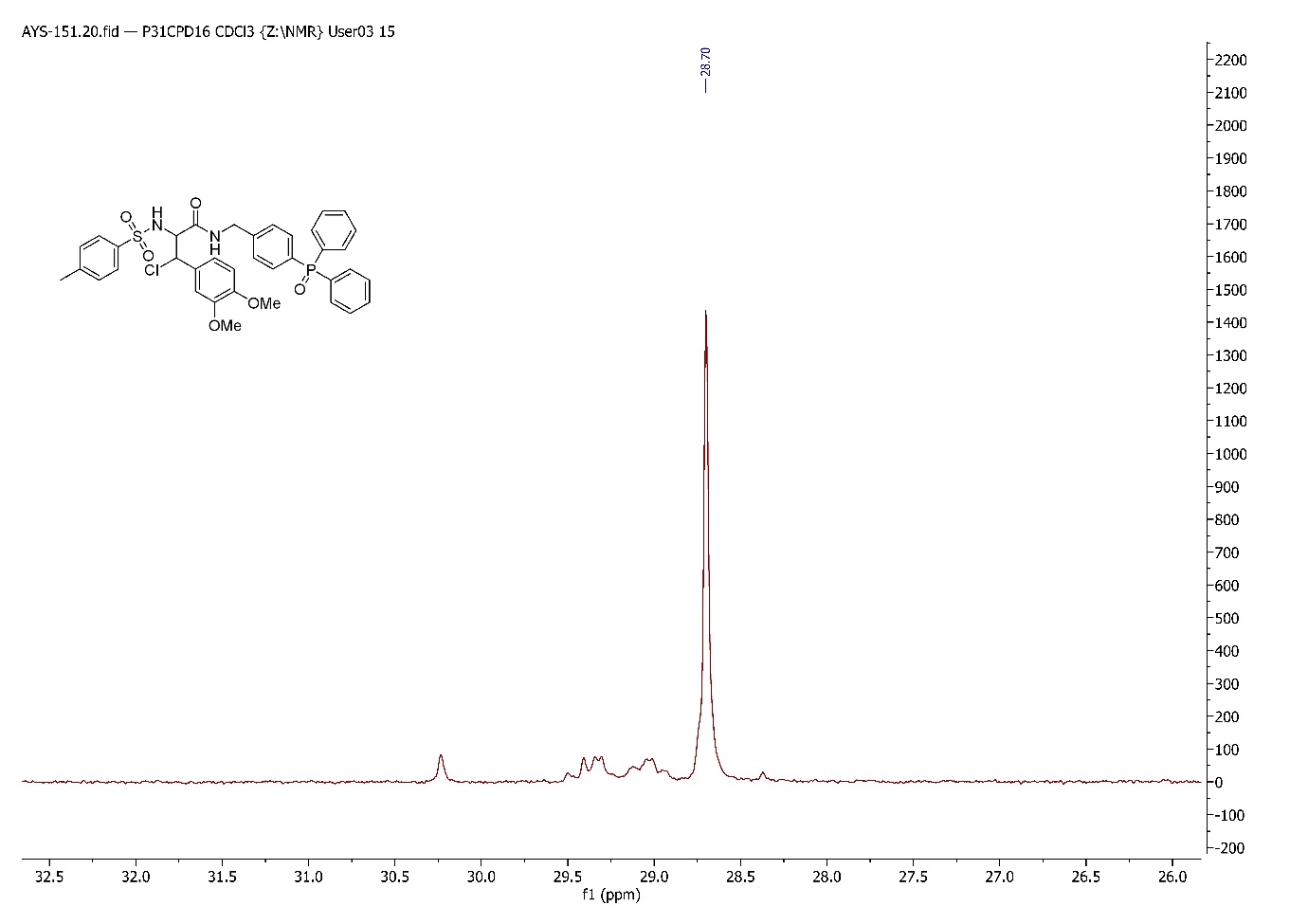

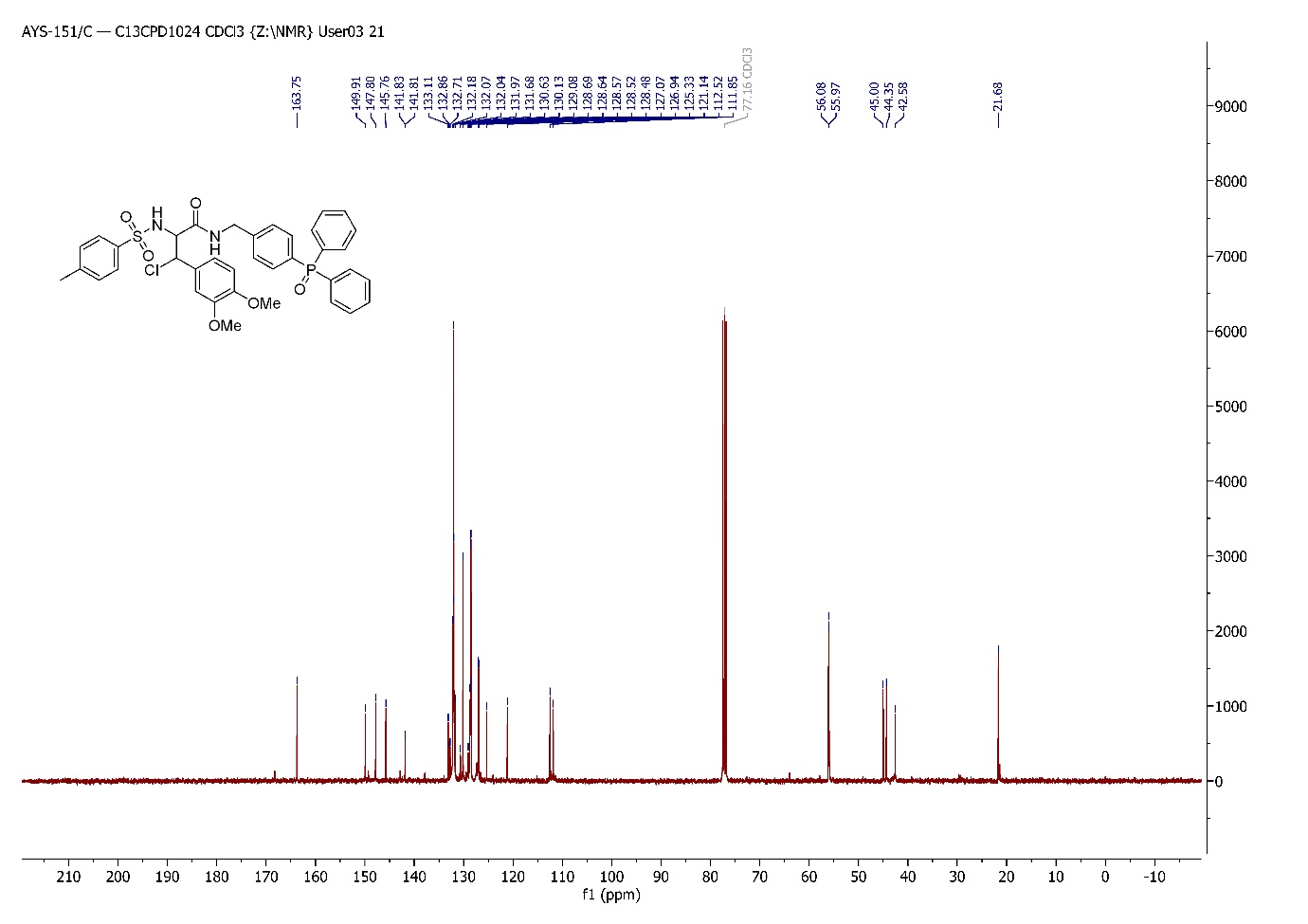

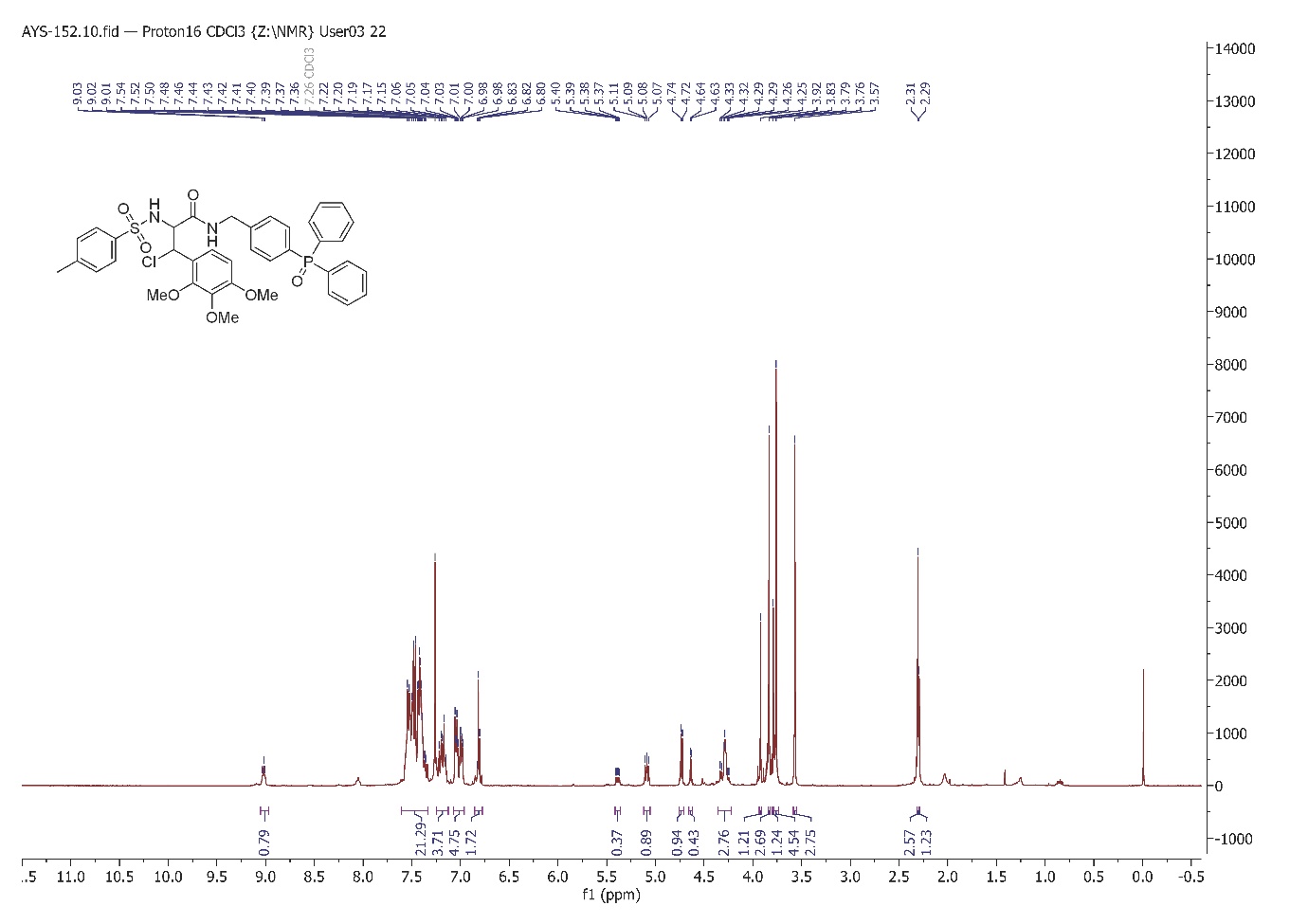

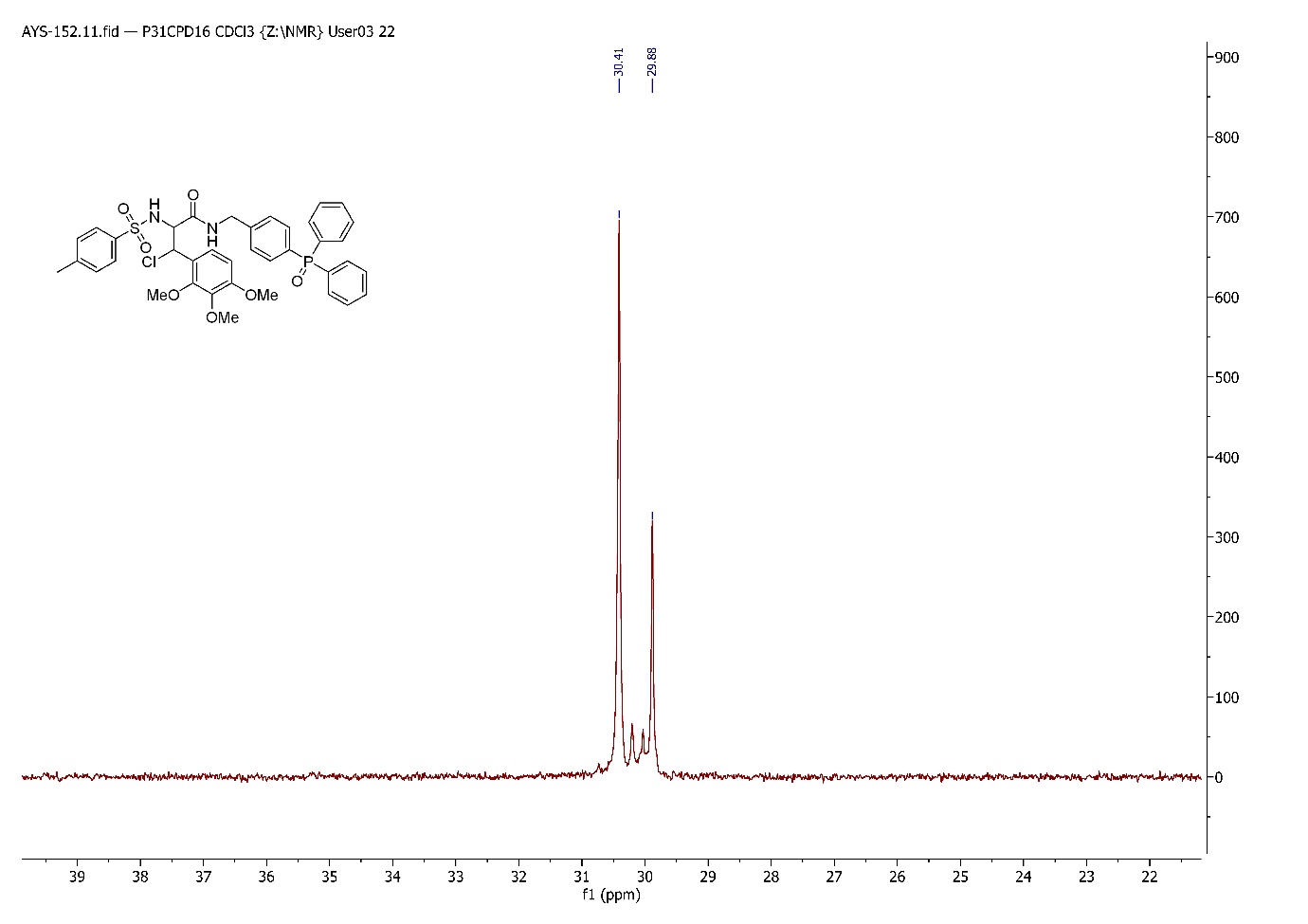

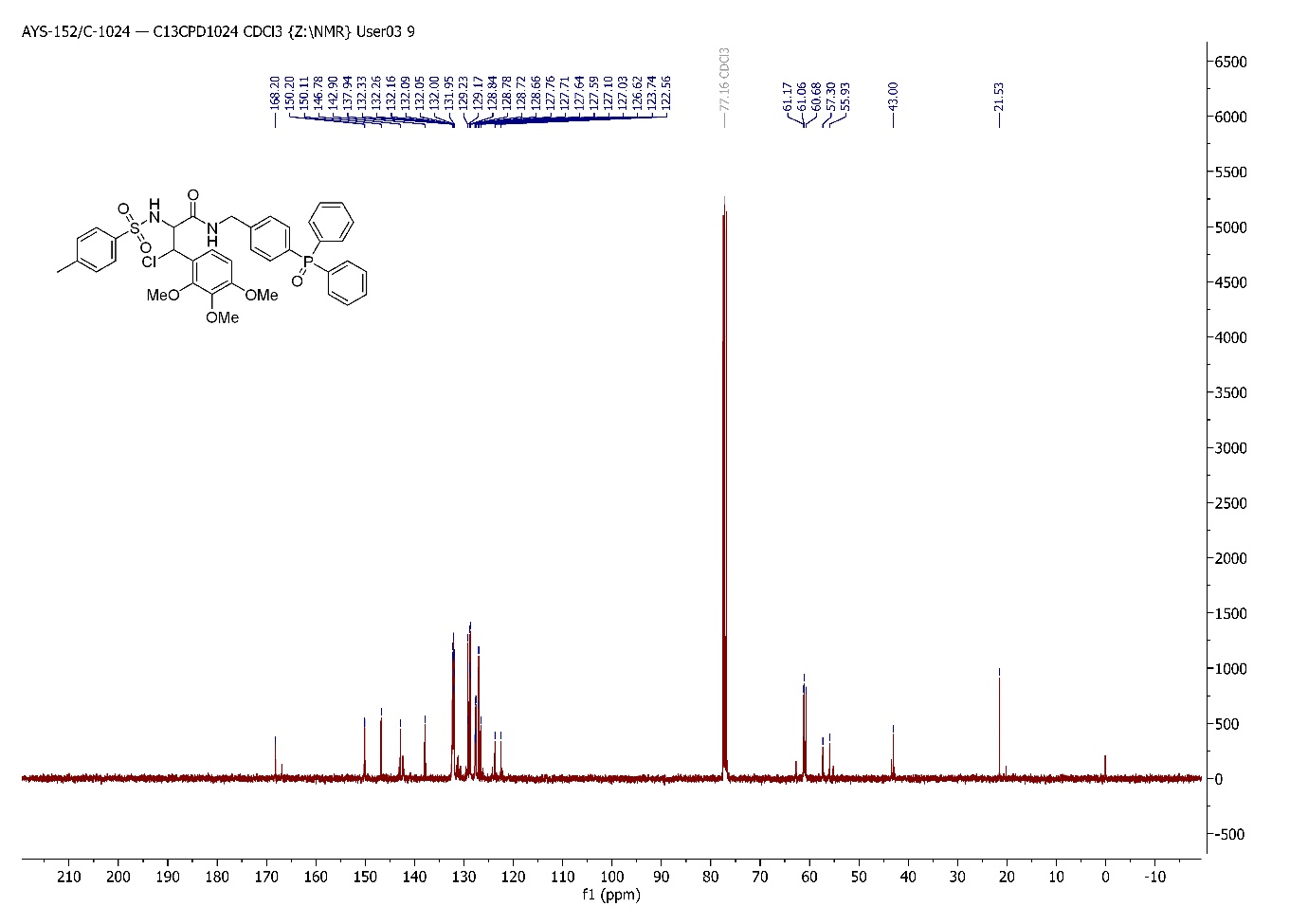

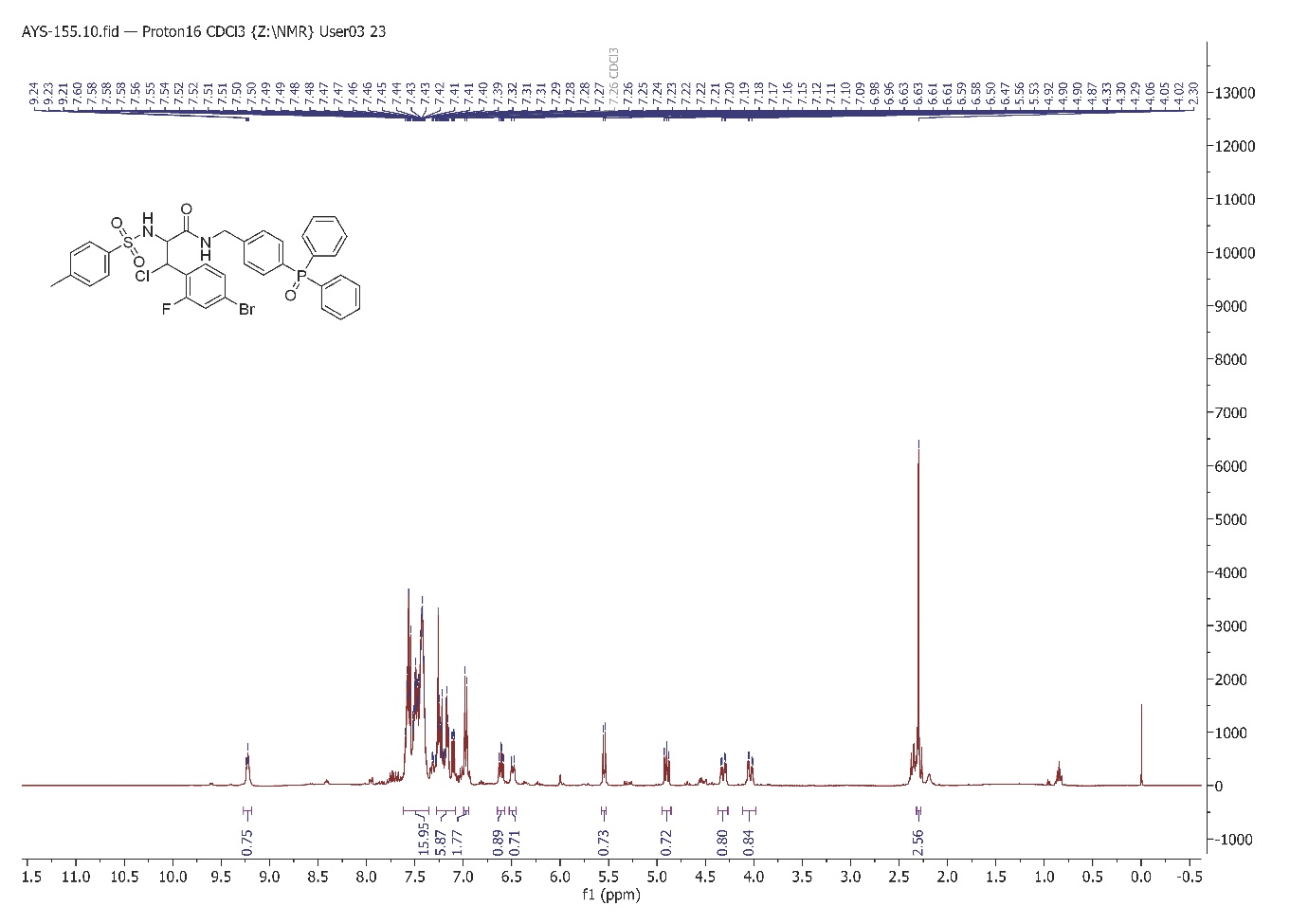

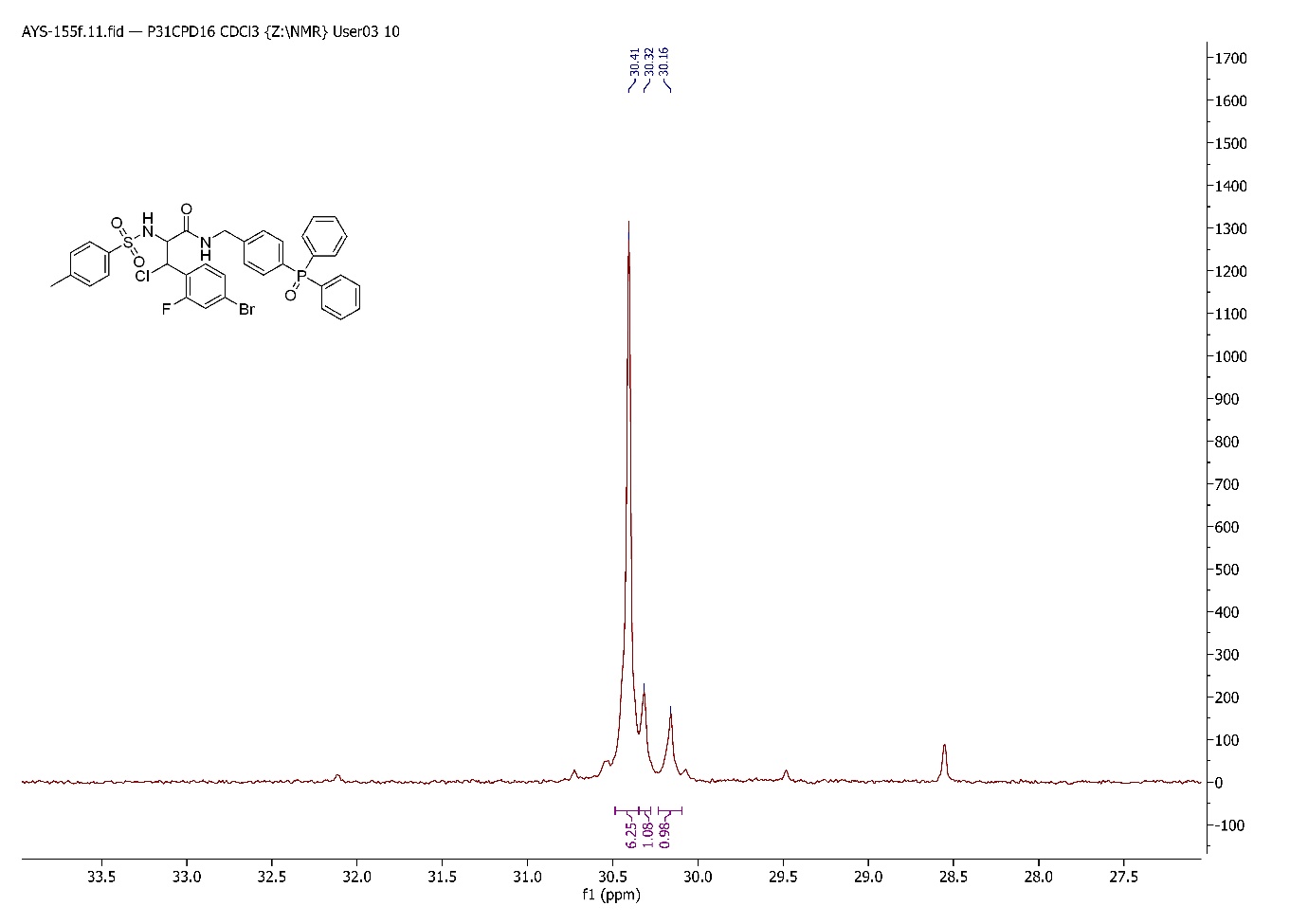

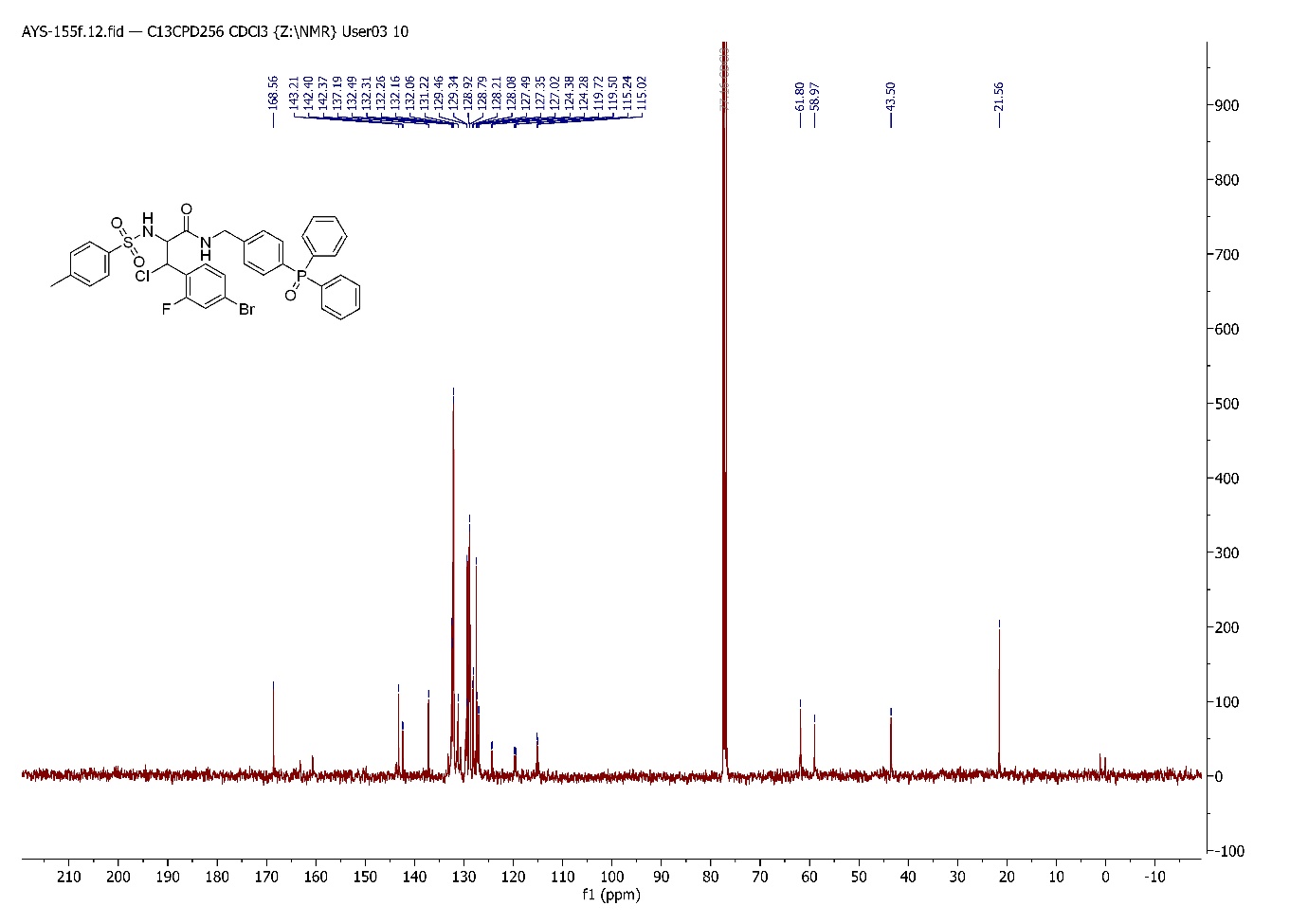

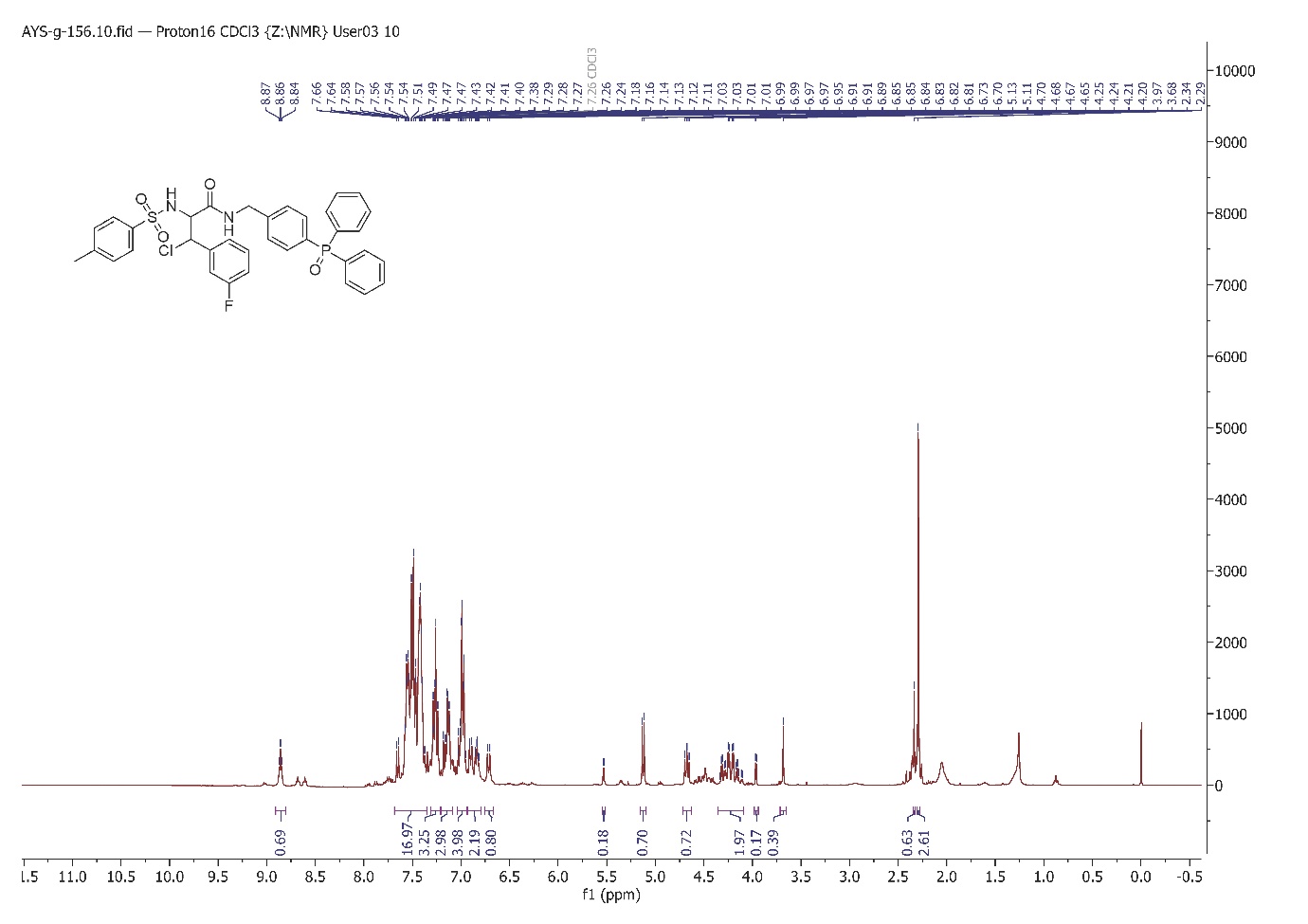

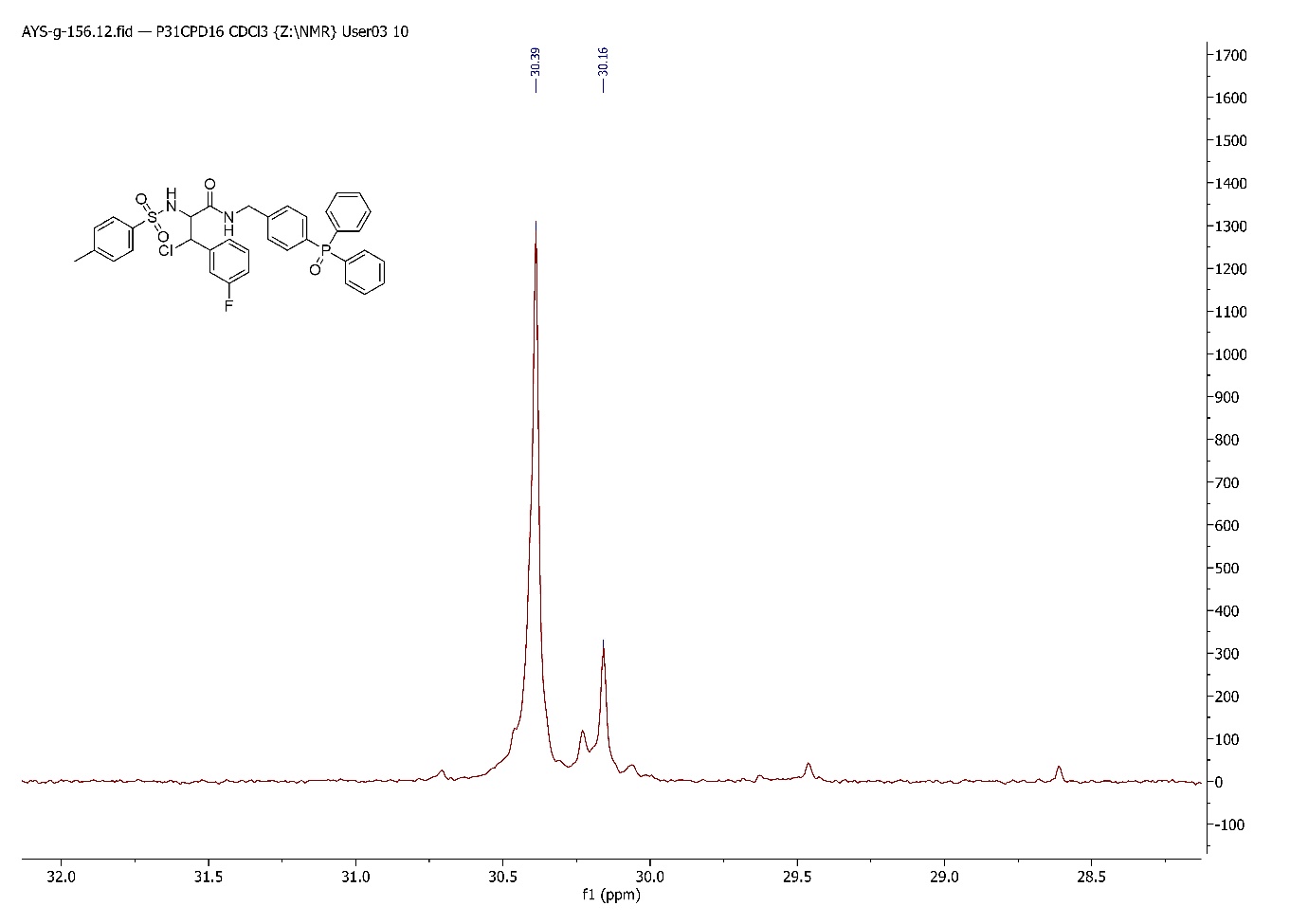

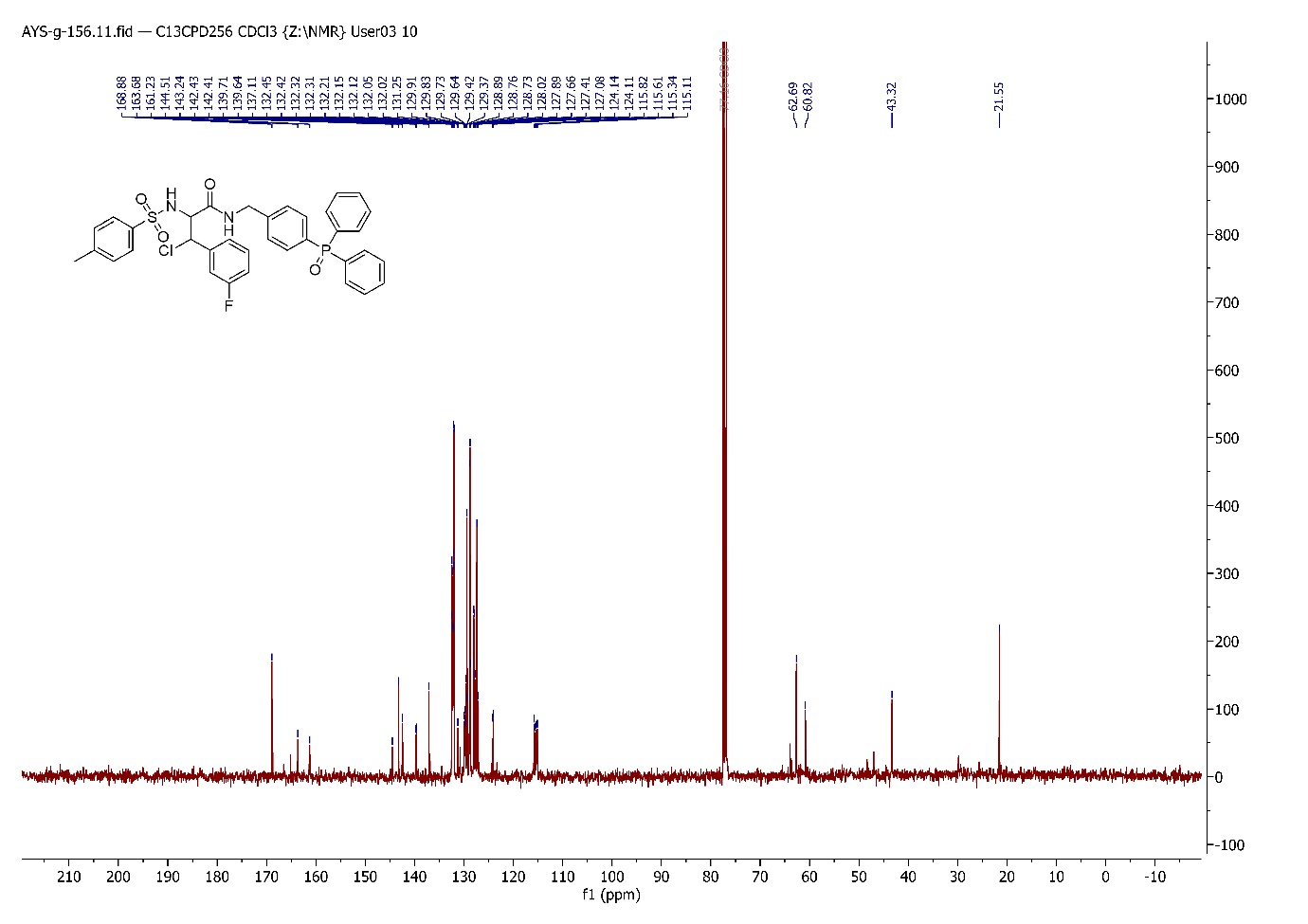

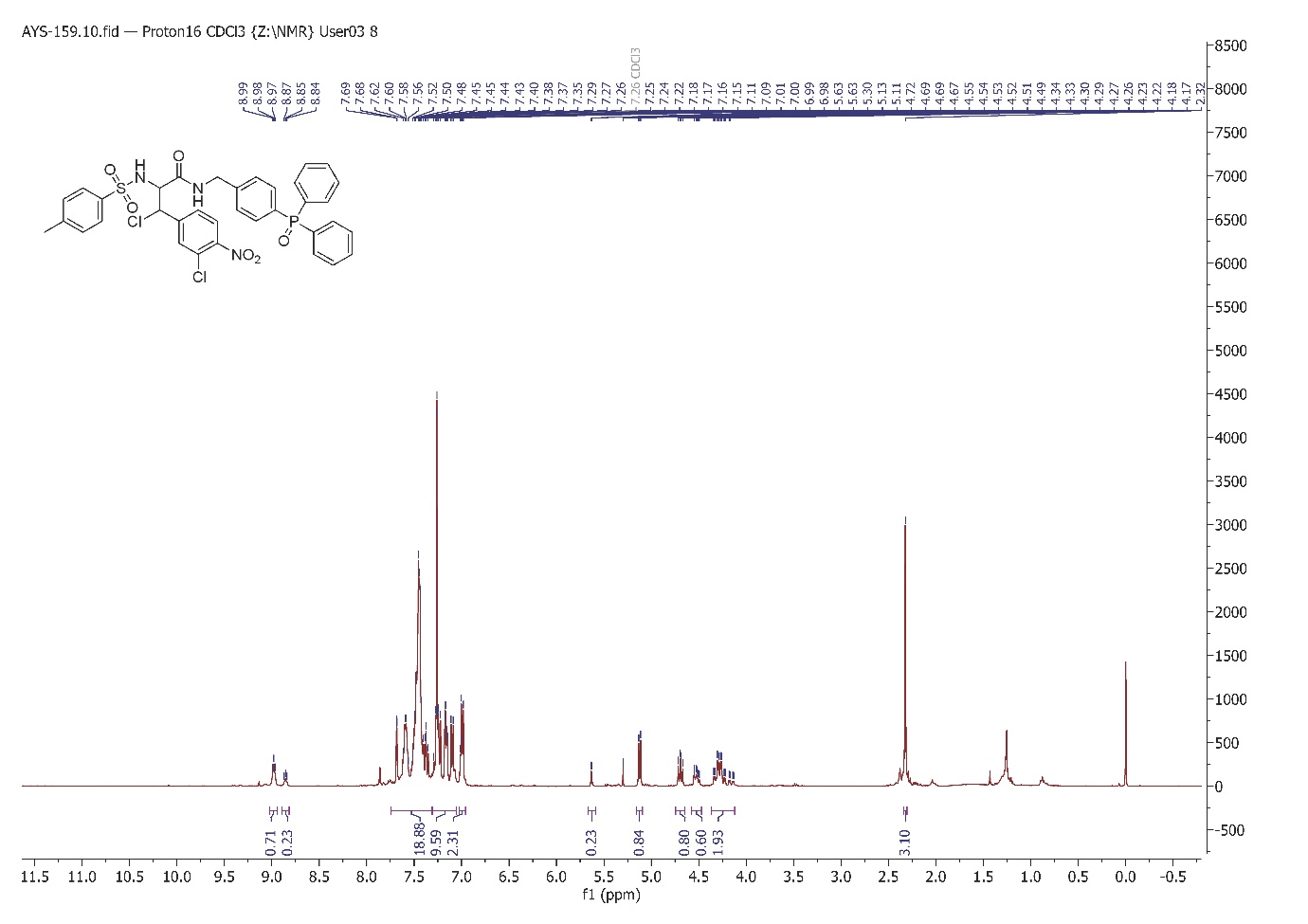

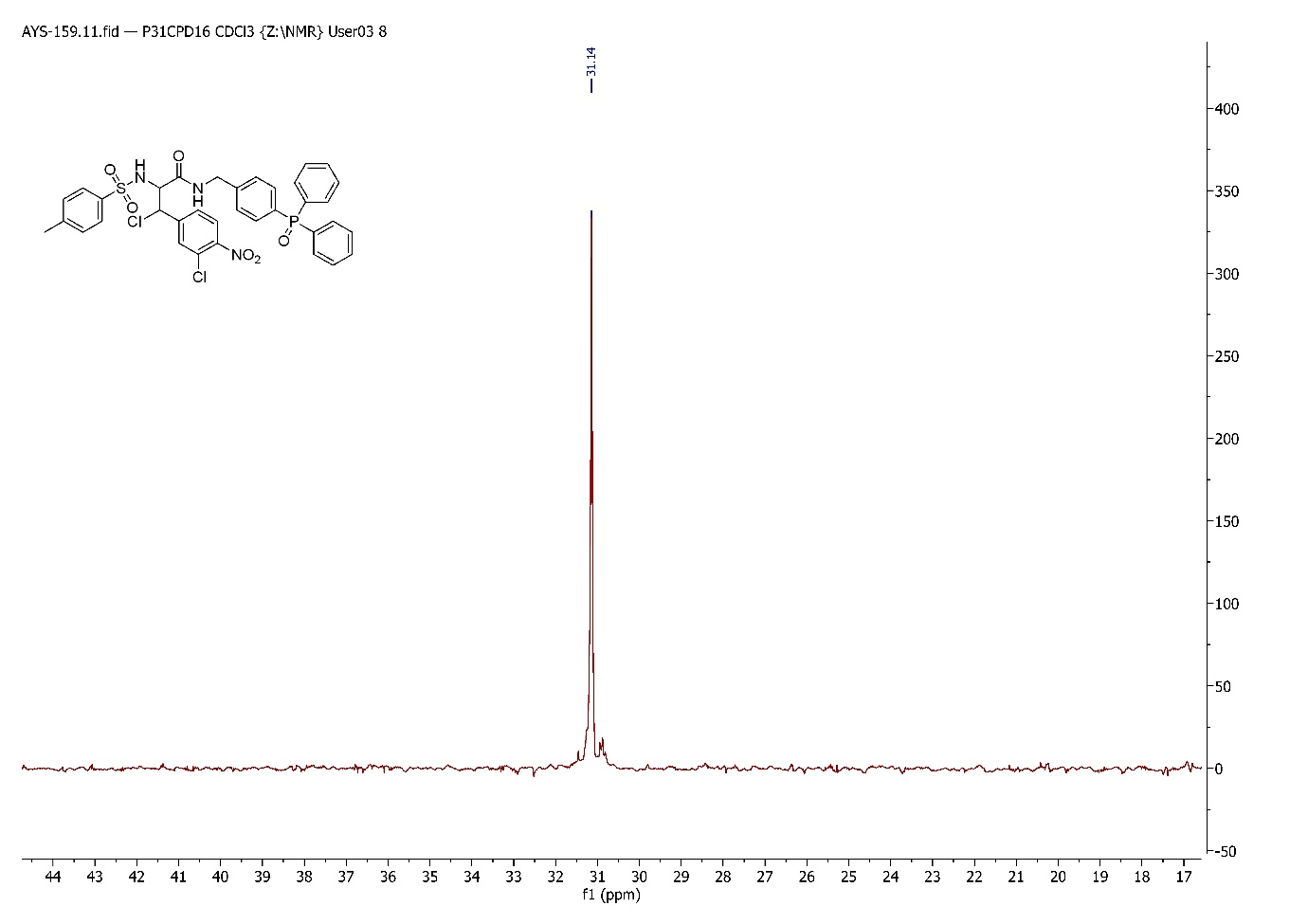

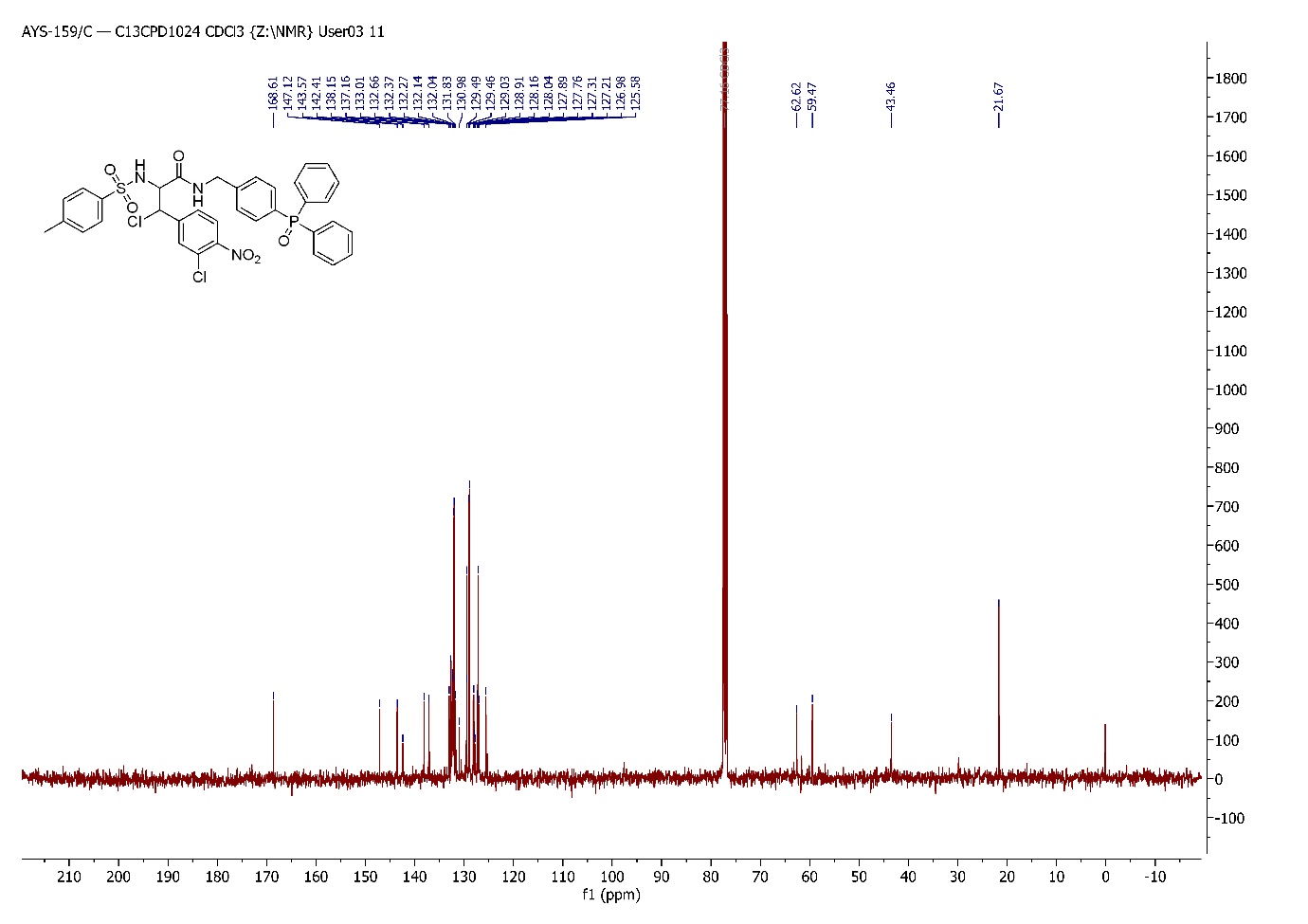


**REFERENCES**

(1) Seifert, C. W.; Paniagua, A.; White, G. A.; Cai, L.; Li, G., GAP Peptide Synthesis through the Design of a GAP Protecting Group: An Fmoc/tBu Synthesis of Thymopentin Free from Polymers, Chromatography and Recrystallization. *Eur. J. Org. Chem.* **2016,** *2016* (9), 1714-1719.

(2) Jablonkai, E.; Keglevich, G., Catalyst-free P–C coupling reactions of halobenzoic acids and secondary phosphine oxides under microwave irradiation in water. *Tetrahedron Lett.* **2015,** *56* (13), 1638-1640.

(3) Hingst, M.; Tepper, M.; Stelzer, O., Nucleophilic Phosphanylation of Fluoroaromatic Compounds with Carboxyl, Carboxymethyl, and Aminomethyl Functionalities − an Efficient Synthetic Route to Amphiphilic Arylphosphanes. *Eur. J. Org. Chem.* **1998,** *1998* (1), 73-82.

(4) Janssen, M.; Müller, C.; Vogt, D., ‘Click’ Dendritic Phosphines: Design, Synthesis, Application in Suzuki Coupling, and Recycling by Nanofiltration. *Adv. Synth. Catal.* **2009,** *351* (3), 313-318.
